# Locked down, locked out: a cross-sectional study on experiences of intimate partner violence (IPV) and barriers to formal and informal support during COVID-19 lockdowns in Ontario

**DOI:** 10.1186/s12889-025-25124-7

**Published:** 2025-11-17

**Authors:** Dina Idriss-Wheeler, Pamela Surkan, Shannon Bainbridge, Ziad El-Khatib

**Affiliations:** 1 Interdisciplinary School of Health Sciences, University of Ottawa, Ottawa, Canada; 2 Bloomberg School of Public Health, Johns Hopkins University, Baltimore, United States of America; 3 Department of Cellular and Molecular Medicine, University of Ottawa, Ottawa, Canada; 4 Department of Global Public Health, Karolinska Institutet, Stockholm, Sweden

**Keywords:** Intimate partner violence (IPV), COVID-19 pandemic, Formal support, Informal support, Barriers, Canada

## Abstract

**Background:**

The COVID-19 pandemic intensified pre-existing social and health inequities, with individuals actively experiencing intimate partner violence (IPV) facing heightened risks and barriers to support. While lockdowns were necessary for public health, they also increased isolation and limited access to essential formal and informal supports. This study explores the association between IPV experienced during Ontario’s COVID-19 lockdowns (March 2020–June 2021) and barriers to both formal (e.g., health, legal, housing) and informal (e.g., friends, family) support systems.

**Methods:**

A cross-sectional online survey was conducted with 1,344 participants, 18 years or older, who were in an adult relationship and residing in Ontario, Canada during the lockdowns. Participants were recruited through non-probability convenience, quota-based sampling from a pre-existing online panel (Leger Opinion, LEO). Data were analyzed using descriptive statistics, chi-square tests, and multivariable logistic regression to examine associations between IPV and support barriers, controlling for demographic, relational, and health-related factors.

**Results:**

Nearly one in four participants (23.4%) self-identified as experiencing IPV (i.e., *emotional*,* sexual*,* physical*,* mental*,* financial*,* coercive*,* spiritual and/or technology-based abuse*) during the lockdowns. IPV survivors had over three times greater odds of facing multiple barriers to formal supports (aOR = 3.4; 95% CI: 2.16–5.38) and 1.6 times higher odds of decreased communication with friends or family (aOR = 1.6; 95% CI: 1.06–2.31). Risk factors for reduced access included low household income, informal caregiving responsibilities, and perceived community violence. Poor physical and mental health were also significant predictors of reduced access to formal and informal supports.

**Conclusions:**

This study highlights how COVID-19 lockdowns compounded access barriers for participants who self-identified as IPV survivors, limiting both formal services and informal networks. Emergency preparedness plans should maintain IPV service capacity during lockdowns through essential service designations, implement technology-based interventions such as discreet online platforms and text-based safety planning, and create targeted outreach for high-risk groups including low-income households and caregivers. More inclusive frameworks are also needed to ensure that supports are responsive to all survivors—particularly as men and women in this study reported similar IPV experiences and outcomes during lockdowns. Future pandemic responses must proactively fund IPV services rather than rely on reactive, underfunded crisis interventions.

**Supplementary Information:**

The online version contains supplementary material available at 10.1186/s12889-025-25124-7.

## Background

Intimate partner violence (IPV) includes behaviours within an intimate relationship that cause physical, sexual, financial or psychological harm, including physical aggression, sexual coercion, psychological abuse, and controlling behaviours [1, 2]. In Canada, 44% of women and 36% of men have experienced some form of IPV in their lifetime [3]. Aptly described by Young-Wolff et al. (2016) and Jack et al. (2023), IPV is a global ‘wicked’ problem that is complex with multiple intersecting social, economic, environmental and political factors that negatively impact the wellbeing of survivors and their families [4, 5]. It can lead to severe short- and long-term consequences, including mental health challenges (e.g., PTSD, depression, anxiety), increased risky behaviours, physical injuries, financial instability, homelessness, and disruptions to academic, professional, and social life, limiting survivors’ ability to reach their full potential [1, 6–11]. Moreover, studies have shown that survivors of IPV encounter significant barriers to accessing health care, contributing to persistent health inequities [11–13].

Several studies have suggested formal and informal (perceived or received) supports are beneficial to mental health, physical health and well-being [14, 15]. For individuals who have experienced IPV, the importance of formal (i.e., health care, mental health, legal counsel, housing, transportation, community-based organizations, child protection) and informal (i.e., friends, family, neighbours) supports for improved health outcomes is well documented in literature [16–18]. Social supports, particularly informal, mean closer relationships (i.e., increased psychological and material resources) that lead to better mental health [19, 20]. For Survivors of IPV, better social supports, and even perception of supports, are a protective factor (i.e., increased safety net) because the emotional and tangible support reduces susceptibility to negative psychological impacts of IPV [16, 18]. Similarly, both formal and informal supports have been shown to protect individuals who experience physical violence [16]. Strong informal support networks have been shown to reduce experience of less severe physical violence over the course of a year [21]. Of note, those who used formal services (i.e., shelters, civil protection, legal advocacy services) were less likely to experience IPV [16]. A systematic review by Ogbe et al. (2020) found that advocacy and case-management interventions with strong community linkages enhanced IPV survivors’ access to social supports (e.g., resources, coping strategies) and improved mental health outcomes for survivors [17].

The 2019 novel coronavirus (COVID-19) disrupted lives globally [22]. In Canada, COVID-19 resulted in over 4.6 million cases and 51,000 deaths, leading provincial governments to implement restrictive public health measures to curb the spread of the virus [23]. These measures, including lockdowns and stay-at-home orders, were critical in protecting public health but had unintended and far-reaching consequences for marginalized populations. Ontario, the province with the highest number of confirmed COVID-19 cases in Canada, implemented three distinct lockdown periods between March 2020 and June 2021 [24, 25]. These lockdowns, guided by the Chief Medical Officer of Health and issued by the Premier of the province, aimed to save lives, prevent health care system collapse, and curb virus transmission [26]. Measures included school closures, bans on social gatherings, restrictions on non-essential health care and social services, and work-from-home mandates [25]. While these actions were necessary for public health, they significantly exacerbated social isolation and limited access to critical supports for IPV survivors, effectively locking them out of essential formal and informal support systems [27].

Stressful life events (SLEs) - such as pandemics - amplify distress, fear, and anxiety among impacted populations and communities [28]. There are sub-groups within SLE affected communities who often face heightened risks and worsening of pre-existing health and social inequities. Globally, and according to the WHO, common groups considered high-risk during emergencies or disasters include people living in poverty, women, children, youth, older people, people with disabilities, people with chronic illness, migrants, ethnic minorities, Indigenous and sexual minorities [29]. Similarly, Public Safety Canada’s National Risk Profile has identified groups known to be vulnerable during disasters, including, Indigenous communities, low-income populations, elderly individuals, persons with disabilities, and remote or northern communities in the Canadian context [30]. Notably, neither one of these documents focusing on health and emergency disaster risk management and public safety explicitly identifies individuals who experience IPV as a key group to consider as a vulnerable or high-risk group [30]. However, several literature reviews have shown that survivors of IPV are at higher risk in terms of limited access to supports, worsening mental health and increased exposure and exacerbation of already existing IPV in contexts of SLEs such as economic instability, climate events, and disasters [31–33]. This omission may lead to gaps in funding, service prioritization, and policy preparedness, further marginalizing IPV survivors during emergencies and excluding them from coordinated public health and safety responses.

SLEs often result in policy shifts and resource reallocation that disproportionately affect marginalized groups. For instance, institutional responses to climate crises can lead to gaps in service delivery, further compounding the mental health and social inequities already experienced by these at-risk populations. A 2024 scoping review, including twelve studies from Canada, reported that SLEs were associated with increased IPV experiences and key barriers to service access in high-income countries, including diverted resources, disrupted social networks, and exacerbated inequities [36]. Lockdown mandates and policies during the COVID-19 pandemic further restricted IPV survivors’ access to essential formal (health and social services) and informal (family and friend) supports [36]. While these studies examined COVID-19’s impact on IPV services, providing valuable insights into survivor and service provider experiences, there remain gaps in understanding the broader scope and patterns of these impacts. Of the twelve Canadian studies identified in the scoping review, only one used a quantitative approach, with the remainder employing qualitative methods that offered rich, in-depth perspectives on lived experiences [36]. Most studies (10 of 12) focused primarily on service provider perspectives or mixed perspectives, with only two specifically examining survivor outcomes. While these qualitative studies have been instrumental in understanding the mechanisms and contexts through which COVID-19 affected IPV services, quantitative research is needed to complement this knowledge by examining patterns of barriers across larger, representative populations. Specifically, no studies have quantitatively examined survivors’ experiences of barriers to both formal and informal support access simultaneously during COVID-19 lockdowns, nor compared these experiences between IPV survivors and non-survivors using a large, population-based sample in Ontario, the most populous province in the country. Exploring the relationship between IPV experiences and barriers to formal and informal supports during COVID-19 is important for understanding the broader implications of lockdown measures on this high-risk population.

This manuscript addresses the research question, *what was the association between experiences of IPV during COVID-19 lockdowns in Ontario and barriers to formal* (i.e., health care, mental health, legal counsel, housing, transportation, community-based organizations, child protection) *and informal supports (e.g.*,* friends and family)?* While lockdowns and restrictive policies affected everyone’s access to health and social supports, we expected that IPV survivors, would face disproportionately greater challenges. First, we hypothesized that IPV survivors experience more barriers to formal supports and reduced communication with friends and family (i.e., informal supports) compared to individuals who did not experience IPV. Second, and based on existing IPV literature in Canada [34], the majority of individuals impacted by IPV would identify as women or gender diverse in this study, making them more likely to encounter barriers to both formal and informal supports during COVID-19. Third, consistent with the complex nature of IPV as a problem with multiple intersecting social, economic, environmental and political factors, we expected that sociodemographic characteristics (i.e., income, caregiving responsibilities, community context) would independently influence support access barriers during crisis periods.

## Methods

### Study design and setting

This study used a cross-sectional cohort design to gather data from individuals aged 18 and above residing in Ontario, focusing on self-identified experience of IPV and its association with access to formal and informal supports during the COVID-19 lockdowns. The survey, available in both English and French, was developed using the SurveyMonkey^®^ platform, a cost-efficient and highly secured tool for online survey design and distribution [35]. The survey was disseminated to participants through Leger’s online market research platform, LEO [36]. Leger integrates SurveyMonkey into its process, enabling efficient distribution of the survey to its panelists via established channels. Panelists were recruited by Leger through random selection facilitated by its call centre. To maintain panel quality, Leger implements a double opt-in process, regularly updates panelist profiles, and uses screening measures to prevent duplicate accounts and fraud. Surveys are distributed to panelists via the LEO platform, accessible through the website or mobile app, with invitations sent via email or push notifications.

### Participant recruitment and sample

Leger distributes surveys to its panelists primarily through email invitations, targeting individuals based on a sampling frame that reflects Ontario’s 18 + population demographics (see Additional file 1 for Leger sampling frame for this study). Panelists receive a unique link to the survey, which can only be used once, preventing multiple responses from the same individual. The surveys are designed to be user-friendly and accessible on various devices, including computers, tablets, and smartphones, making it convenient for participants to complete them. To encourage participation, Leger offers rewards and incentives, which vary depending on the length and complexity of the survey [37]. Only the research team had access to the participants’ anonymized data – no personal identifiers are linked to participants.

The participants in this study were over 18 years of age, resided in Ontario during COVID-19 lockdowns, reported being in an adult romantic/intimate relationship at the time. Individuals who had broken up before or during COVID-19 lockdowns (March 2020) and continued to experience IPV post-break-up were included.

There were a total of 1,362 responses to the survey. A total of 18 responses were excluded due to ineligibility (e.g., screened in by error even though respondents were not in a relationship, or their partners had died before COVID-19). This resulted in a final sample of 1,344 responses.

Data cleaning was performed, with no programmatic imputations applied. However, logical imputations were applied in specific cases. For gender of partner, if the respondent had indicated the type of partner (i.e., wife, husband, boyfriend, girlfriend), the gender was imputed where possible (i.e., woman for wife or girlfriend, man for husband or boyfriend). For missing data on race, gender, partner type, imputed “prefer not to say”. For missing citizenship status, income, employment and partner education, imputed “unknown”. For geography, imputed the corresponding geography only if a postal code was given, otherwise missing. For community violence and information during COVID, imputed “not sure” for missing cases based on comments provided in “other”, otherwise missing.

### Variable selection and measurement

#### Experience of IPV (main independent variable) 

The survey instrument was co-created with IPV survivors and service providers and underwent two rounds of community testing before deployment to ensure cultural appropriateness and comprehensibility.

Survey respondents were initially asked if they were in an adult relationship and experienced IPV during COVID-19 lockdowns in Ontario (March 2020-June 2021). Based on established definitions in literature and the region [1, 2, 38] and consultation with community-based partners, IPV was defined in the survey as follows: “Intimate partner violence includes emotional, sexual, physical, mental, financial, coercive, exploitative, spiritual or technology-based abuse by your intimate partner with whom you are/were in an adult relationship.”

Based on recommendations from community-based partners, hyperlinks to definitional resources were provided within the survey for two emerging forms of violence—technological and spiritual/religious abuse—to ensure participants had clear understanding of these less commonly recognized forms of IPV. This comprehensive definition aligns with contemporary understanding of IPV as encompassing multiple forms of harm beyond physical violence.

Participants who responded affirmatively and self-identified as experiencing IPV were then directed to complete an IPV-specific module based on the validated Composite Abuse Scale (Revised) – Short Form (CASR-SF), used with permission from the developers [39]. The CASR-SF is a psychometrically sound instrument that assesses multiple dimensions of intimate partner abuse through specific behavioral indicators, providing additional validation of participants’ IPV experiences.

Participants were classified as experiencing IPV (yes IPV) if they self-reported abuse either: (1) before COVID-19 lockdowns and continuing during lockdowns, or (2) newly occurring during COVID-19 lockdowns, whether in ongoing or post-separation relationships. All others were coded as “no IPV.”

Lockdowns were described to participants as periods “declared by the Government of Ontario during the pandemic to issue a stay-at-home order to reduce spread of infection in the province, keep people safe and not overwhelm the health care system.”

#### Barriers to accessing formal support services (main outcome/dependant variable #1)

 The survey assessed participants’ access to 12 formal support services during the COVID-19 lockdowns by asking “*Did you experience any barriers trying to access the following services?*” These services included: community-based organizations, counseling, health care, crisis lines, child protection, legal counsel, mental health, emergency shelters, settlement agencies, addiction services, housing, and transportation. Responses were recoded into dummy variables with three categories: “yes”, “no”, and “did not try”. Analyses compared individuals who experienced IPV during the lockdowns with those who did not.

To evaluate overall access challenges, a composite variable was generated by summing the number of “yes” responses across the 12 services. This captured the cumulative barriers faced by respondents who attempted to access support. Centile analysis of the composite variable revealed that the 75th percentile corresponded to fewer than two (2) reported barriers. Consequently, a threshold of > 2 barriers was established to identify individuals with substantial access challenges. Respondents reporting > 2 barriers were classified as experiencing “yes barriers,” indicating significant difficulties in accessing services, while those with *≤* 2 barriers were categorized as having “no barriers,” reflecting minimal to moderate challenges. This cutoff effectively differentiated between high and low levels of service access difficulty.

#### Communication with family and friends – informal supports (main outcome/dependant variable #2)

Participants were asked, “*Did your weekly habits for communicating with family/friends change during COVID-19 lockdowns?*” with response options: “increased”, “decreased”, or “no change”. Responses were recoded into categorical variable for both family and friend communication. Analyses compared individuals who experienced IPV during the lockdowns with those who did not. To assess overall reductions in informal support, a composite binary variable - “decreased communication”- was created. Participants who reported decreased communication with either friends or family were coded as 1, indicating a decline in social interaction; those who reported no decrease in either domain were coded as 0. This variable served as a proxy measure for diminished access to informal support networks during the lockdown period.

#### Potential confounders

To reduce potential confounding and bias in the relationship between IPV and access to formal and informal support, a set of control variable were included in the predictive models. These variables were identified through a scoping review [38], consultation with IPV survivors and violence against women service providers, and the research team’s subject-matter expertise.

Key sociodemographic data were collected, including gender, education, and immigration status guided by Statistics Canada standards [39–41]. Questions on race and Indigenous identity were adapted from the Canadian Institute for Health Research’s (CIHI) [42]. Age was categorized into six groups: 18–24,25–34,35–44,45–54,55–64, *≥* 65. Geographic location was classified according to Ontario’s violence against women (VAW) service regions: Eastern, Central, City of Toronto, Suburban “905” belt, Wester, and Northern. Income was grouped into four categories: <$40,000; $40–69,999; $70–99,999; *≥*$100,000 CAD. Employment status during the pandemic (for both the participant and their partner) was recorded as a binary variable [yes/no]. A measure of perceived community violence was also included, based on a community partner recommendation and supported in the literature as a potential mediator of IPV [43, 44]. Participants were asked “*Do you think community violence was a problem where you lived during the pandemic (March 2020-June 2021)*?” with response options of “No” or “Yes”.

#### Covariates

To account for factors potentially associated with access to formal and informal supports, additional covariates were included. Studies have shown that informal caregiving is linked to reduced health care seeking [45] and increased burden during the COVID-19 pandemic [46]. Accordingly, participants provided information on caregiving demands, including changes in caregiving responsibilities (categorized as “increased”, “decreased”, or “no change”), whether they were the primary caregiver for children or other dependants (“yes”, “no”), and the number of children under their care. Participants were also asked whether they had sufficient information about available services and how to access them during the pandemic (“yes”, “no”). This variable was included to assess knowledge as a facilitator or barrier to support access.

Given the established association between partner substance use and IPV [47, 48], as well as its influence on help seeking behaviours [49, 50], a composite measure was established to capture substance-related relationship strain. Participants responded to statements regarding partner alcohol, cannabis, and illicit drug use (e.g., **“***I wished my partner would not drink so many drinks/engage in substance use*”; “*my partner’s drinking/substance use is a source of strain in our relationship*”, and “*I considered leaving because of drinking/substance use*”). Responses were dichotomized: “Always”, “Often”, and “Sometimes” were coded as 1, indicating a presence of the issue; “Seldom” and “Never” as 0, indicating absence of the issue. These binary variables were summed to generate a ‘Substance Issue Score’ (range 0–6), which was then categorized as “Low Impact” (scores ≤ 2) or “Moderate/High Impact” (scores >2) based on theoretical reasoning that scores above 2 indicated more pervasive (i.e., sometimes, often, always) substance-related relationship strain.

To assess perceived health status, participants were asked how the COVID-19 lockdowns affected their physical and mental health (e.g., “*Thinking about your overall physical health - which includes illness and injury - how did COVID-19 lockdowns (March 2020-June 2021) affect your physical health*?”, and “*Thinking about your overall mental health - which includes stress*,* depression*,* anxiety and problems with emotions - how did COVID-19 lockdowns (March 2020-June 2021) affect your mental health?*”), with response options ranging from “much better” (1), to “much worse” (5).” Binary variables were created for both physical and mental health domains: responses indicating improved or unchanged health status (categories 1–3) were coded as 0, and those indicating “worse” or “much worse” (categories 4–5) were coded as 1. These variables served to identify individuals who experienced decline in health during pandemic period.

Drawing on Berkman and Krishna’s (2014) conceptual framework of how social networks impact health [20], the authors included decreased communication with family/friends and barriers to formal support access as covariates in both models to account for the hypothesized bidirectional relationship between informal and formal support systems. Disruptions to informal networks may increase reliance on and barriers to formal services, while difficulties accessing formal supports may intensify social isolation and reduce communication with informal networks. By including both variables as covariates, the models control for these potential effects.

### Statistical analysis

Descriptive statistics (proportions) and bivariate chi-square tests were used to assess statistical significance between groups (IPV & non-IPV during the COVID-19 lockdowns). Multivariable logistic regression analyses were used to examine the relationship between IPV experience during lockdowns and barriers to accessing formal and informal supports. Statistical significance was set at *p* < 0.05 for all analyses. The data was analyzed using the software package STATA version 18 [51]. Detailed syntax for coding structure and running the analysis is available (see Additional file 2).

### Exploratory mediation and sub-analyses

Exploratory mediation models were used to assess whether poor mental or physical health partially mediated the relationship between IPV and each outcome (i.e., barriers to formal supports and decreased communication with informal supports). These models followed a standard three-step approach: (1) estimating the total effect of IPV on the outcome; (2) assessing the association between IPV and the proposed mediator(s); and (3) examining the effect of IPV on the outcome while adjusting for the mediator(s). Evidence of mediation was inferred when the effect of IPV was attenuated and the mediator remained statistically significant. Second, to assess whether patterns of decreased informal communication varied by relationship type, sub-analyses were conducted examining decreased communication with family and friends separately. These were compared to the composite variable used in the primary analysis. Full results are provided in Additional File 3.

This study adhered to the Strengthening the Reporting of Observational Studies in Epidemiology (STROBE) guidelines for cross-sectional studies, and the completed checklist is included in Additional File 4 [52].

## Results

### Descriptive statistics

The descriptive statistics of the survey respondent are presented in Table 1. Of the 1,344 participants, 23.4% (*n* = 314) self-reported experiencing IPV during COVID-19 lockdowns (Yes-IPV), while 76.6% (*n* = 1,030) reported no IPV experience (No-IPV). A higher proportion of younger participants reported experiencing IPV (18–34 years: 46.3% Yes-IPV vs. 16.4% No-IPV, *p* < 0.001), while older participants were underrepresented in the Yes-IPV group (55 + years: 7.7% Yes-IPV vs. 44.0% No-IPV, *p* < 0.001). Racialized and Indigenous participants (47.5% Yes-IPV vs. 20.8% No-IPV, *p* < 0.001), immigrants and non-permanent residents (15.86% Yes-IPV vs. 5.37% No-IPV, *p* < 0.001), and those in LGBTQ2 + relationships (31.5% Yes-IPV vs. 9.6% No-IPV, *p* < 0.001) were more likely to experience IPV during the pandemic. There was no statistically significant difference in gender distribution between participants who reported experiencing intimate partner violence (IPV) and those who did not, with similar proportions identifying as women (48.1% vs. 49.0%), as men (48.7% vs. 49.6%), and as gender diverse or preferring not to say (3.2% vs. 1.4%), χ²(2) = 4.6, *p* = 0.100.

**Table 1:** Descriptive statistics by experience of IPV during COVID-19 lockdowns in Ontario, Canada

Variable	YES IPV		NO IPV		χ² (df)	*p*-value
	N	%	N	%		
Participant Characteristics						
*Gender*	312		1,026			
Woman	150	48.1	503	49.0	4.6 (2)	0.100
Man	152	48.7	509	49.6		
Gender Diverse & Prefer Not to Say ^#^	10	3.2	14	1.4		
Relationship Type*	311		1,025			
Heterosexual	208	66.9	909	88.7	N/A	0.00
LGBTQ2+^##^	98	31.5	98	9.6		
Prefer not to say	5	1.6	18	1.7		
*Age (in years)*	*312*		*1027*			
18–34	145	46.3	168	16.4	183.9(2)	0.00
35–54	143	46.0	407	39.6		
55 +	24	7.7	452	44.0		
*Race*	314		1,030			
White	157	50.0	783	76.0	87.0(2)	0.00
Racialized and Indigenous	149	47.5	214	20.8		
Prefer not to say	8	2.5	33	3.2		
*Immigrant Status*	309		1025			
Non-immigrant	260	84.14	970	94.63	36.4(1)	0.00
Immigrant & non-permanent resident	49	15.86	55	5.37		
*Education*	312		1,026			
Primary	63	20.2	181	17.6	1.1 (2)	0.57
Trade/Diploma	106	34.0	368	35.9		
University	143	45.8	477	46.5		
*Employment*	313		1030			
Employed	217	69.3	692	67.2	0.50 (1)	0.48
Unemployed	96	30.7	338	32.8		
Partner Characteristics						
Gender*	312		1,029			
Woman	121	38.8	489	47.5	N/A	0.00
Man	180	57.7	526	51.1		
Gender Diverse & Prefer not to say	11	3.5	14	1.4		
*Age (in years)*	305		1,020			
18–34	140	45.9	160	15.7	189.2(2)	0.00
35–54	145	47.5	415	40.7		
55 +	20	6.6	445	43.6		
*Race*	311		1,026			
White	165	53.1	772	75.2	62.6(2)	0.00
Racialized and Indigenous**	137	44.0	220	21.4		
Prefer not to say	9	2.9	34	3.3		
Immigration Status*	309		1024			
Non-immigrant	250	80.91	957	93.46	N/A	0.00
Immigrant & non-permanent resident	59	19.09	67	6.54		
*Education*	306		1,021			
Primary	89	29.1	235	23.0	8.7(2)	0.01
Trade/Diploma	94	30.7	403	39.5		
University	123	40.2	382	37.4		
*Employment Status*	310		1,022			
Employed	203	65.5	653	63.9	0.26(1)	0.61
Unemployed	107	34.5	369	36.1		
Household income	293		950			
<$40,0000	94	32.1	139	14.6	56.2 (2)	0.00
$40,000-$69,999	72	24.5	223	23.5		
$70,0100-$99,999	57	19.5	188	19.8		
$100,000 +	70	23.9	400	42.1		
Geography	313		1,029			
Eastern Ontario	60	19.2	137	13.3	18.7 (4)	0.00
Central Ontario	29	9.3	80	7.8		
Toronto and the GTA (905 Belt)	139	44.4	463	45.0		
Western Ontario	55	17.6	282	27.4		
Northern Ontario	30	9.5	67	6.5		
Community Violence is a problem	314		1,027			
No*	194	61.8	793	77.2	26.5 (1)	0.00
Yes	120	38.2	234	22.8		
Enough Information about Services available during pandemic	312		1,019			
Yes	152	48.7	742	72.8	62.9 (2)	0.00
No	160	51.3	277	27.2		
Impact of Substance Use on relationship	314		1,030			
High/Moderate	193	61.5	92	8.9	397.5 (1)	0.00
Low	121	38.5	938	91.1		
Number of Children	314		1,025			
No children	140	44.6	685	66.8	55.1 (2)	0.00
1–2 children	137	43.7	291	28.5		
3 + children	37	11.7	48	4.7		
Informal Caregiver during COVID-19	314		1,030			
Yes	186	59.2	410	39.81	36.8 (1)	0.00
No	128	40.8	620	60.19		
Physical Health	313		1,027			
Much better/better/no change	185	59.1	762	74.2	26.4 (1)	0.00
Worse/much worse	128	40.9	265	25.8		
Mental Health	313		1,023			
Much better/better/no change	133	42.5	613	60.0	29.5 (1)	0.00
Worse/much worse	180	57.5	410	40.0		

^#^Gender Diverse (n = 12) includes transgender, gender non-conforming, trans woman, trans man, gender fluid. Prefer not to say (*n* = 12). Due to small sample size, we used Fisher’s exact test to determine significance between experience of IPV, and those who did not experience IPV. The small sample size for this category limits the robustness of the results and will be interpreted with caution

^##^LGBTQ2 + Relationship includes Lesbian, Gay, Bisexual, Transgender, Queer, Two-Spirit, and other orientations (pansexual, asexual). *Include entries of “not sure” about community violence being a problem (2 for Yes-IPV, 23 for No-IPV)

*Fisher’s exact test was used due to small cell counts in categorical variables, ensuring appropriate statistical inference when expected frequencies were low

** The term “racialized” refers to individuals who belong to racial minority groups and is standard terminology used in Canadian research and census data to describe non-White, non-Indigenous populations [85]

Household income disparities were also evident, with lower-income participants reporting the highest proportion of IPV (<$40,000: 32.1% Yes-IPV vs. 14.6% No-IPV, *p* < 0.001).

Differences in partner characteristics were notable, with a higher proportion of partners of Yes-IPV participants being racialized or Indigenous (44.0% Yes-IPV vs. 21.4% No-IPV, *p* < 0.001), younger (18–34 years: 45.9% Yes-IPV vs. 15.7% No-IPV, *p* < 0.001), and gender-diverse (3.5% Yes-IPV vs. 1.4% No-IPV, *p* < 0.001).

Significant health disparities emerged between participants who did and did not experience IPV during the pandemic. A substantially greater proportion of Yes-IPV group reported deteriorated mental health (57.5% vs. 40.0%, *p* < 0.001) and physical health (40.9% vs. 25.8%, *p* < 0.001).

Substance use was also more commonly identified as a source of relationship strain among those who experienced IPV (61.5% vs. 8.9%, *p* < 0.001). Participants who experienced IPV were significantly more likely to perceive community violence as a problem compared to those who did not experience IPV.(38.2% vs. 22.8%, *p* < 0.001). Informal caregiving responsibilities were more prevalent among Yes-IPV participants (59.2% vs. 39.8%, *p* < 0.001), as was the reported lack of sufficient information about available services during the lockdown period (51.3% vs. 27.2%, *p* < 0.001). These findings highlight the compounding challenges faced by those experiencing IPV, particularly in relation to health, caregiving burdens, reduced support networks, and informational barriers.

### Barriers to accessing formal support services

Table 2 presents significant disparities in barriers to accessing formal support services between participants who experienced IPV during COVID-19 lockdowns (Yes-IPV) and those who did not (No-IPV). The most frequently accessed services across the sample were health care (68.4%), transportation (37.3%), and mental health services (35.3%). Across all service domains, the Yes-IPV group reported a higher incidence of barriers, whereas the No-IPV group was more likely to indicate no attempt to access these services.

**Table 2:** Comparison of reported barriers to accessing formal support services among individuals with (yes IPV) and without (no IPV) experience of intimate partner violence during COVID-19 lockdowns (March 2020–June 2021)

Pairwise comparison Bonferroni Correction**								
**Variable**	**Total (n = 1344)**		**YES IPV (n = 314)**		**NO IPV (n = 1030)**		*χ²* (df)/Fisher’s Exact test (df)*	**p-value**
	**N**	**%**	**N**	**%**	**N**	**%**		
Health Care	1,342		313	23.3	1,029	76.7		
Tried accessing	918	68.4	243	77.6	675	65.6		**0.00**
Yes barriers			158	50.5	369	35.9	24.6 (2)	**0.00**
No barriers			85	27.2	306	29.7	*p* < 0.00	0.38
Did not try accessing	424	31.6	70	22.4	354	34.4		**0.00**
Transportation	1,339		313	23.4	1,026	76.6		
Tried accessing	499	37.3	186	59.4	313	30.5		**0.00**
Yes barriers			89	28.4	74	7.2	126.4 (2)	**0.00**
No barriers			97	31.0	239	23.3	*p* < 0.00	**0.00**
Did not try accessing	840	62.7	127	40.6	713	69.5		**0.01**
Mental health	1,339		311	23.2	1,028	76.8		
Tried accessing	473	35.3	201	64.6	272	26.5		**0.00**
Yes barriers			110	35.4	80	7.8	194.5 (2)	**0.00**
No barriers			91	29.3	192	18.7	*p* < 0.00	**0.00**
Did not try accessing	866	64.7	110	35.4	756	73.5		**0.00**
Counselling	1,340		312	23.3	1,028	76.7		
Tried accessing	453	33.8	193	61.9	260	25.3		**0.00**
Yes barriers			100	32.1	68	6.6	185.8 (2)	**0.00**
No barriers			93	29.8	192	18.7	*p* < 0.00	**0.00**
Did not try accessing	887	66.2	119	38.1	768	74.7		**0.00**
Community-based Organization	1,342		312	23.3	1,030	76.8		
Tried accessing	419	31.2	178	57.1	241	23.4		**0.00**
Yes barriers			95	30.5	81	7.9	148.8 (2)	**0.00**
No barriers			83	26.6	160	15.5	*p* < 0.00	**0.00**
Did not try accessing	923	68.8	134	43.0	789	76.6		**0.00**
Housing	1,342		313	23.4	1,027	76.6		
Tried accessing	383	28.6	170	54.3	213	20.7		**0.00**
Yes barriers			68	21.7	29	2.8	180.4 (2)	**0.00**
No barriers			102	32.6	184	17.9	*p* < 0.00	**0.00**
Did not try accessing	957	71.4	143	45.7	814	79.3		**0.00**
Crisis Line	1,342		312	23.3	1,029	76.7		
Tried accessing	345	25.7	152	48.6	193	18.8		**0.00**
Yes barriers			62	19.8	22	2.1	166.6 (2)	**0.00**
No barriers			90	28.8	171	16.6	*p* < 0.00	**0.00**
Did not try accessing	997	74.4	161	51.4	836	81.2		**0.00**
Legal Counsel	1,342		313	23.3	1,029	76.7		
Tried accessing	335	25.0	146	46.6	189	18.4		**0.00**
Yes barriers			54	17.3	23	2.2	141.9 (2)	**0.00**
No barriers			92	29.4	166	16.1	*p* < 0.00	**0.01**
Did not try accessing	1,007	75.0	167	53.3	840	81.6		**0.00**
Settlement Agency	1,342		312	23.3	1,029	76.7		
Tried accessing	325	24.2	145	46.5	180	17.5		**0.00**
Yes barriers			37	11.9	12	1.2	140.4 (2)	**0.00**
No barriers			108	34.6	168	16.3	*p* < 0.00	**0.00**
Did not try accessing	1,016	75.8	167	53.5	849	82.5		**0.00**
Addictions Services	1,342		312	23.3	1,029	76.7		
Tried accessing	325	24.2	147	47.1	178	17.3		**0.00**
Yes barriers			48	15.4	14	1.4	160.4 (2)	**0.00**
No barriers			99	31.7	164	15.9	*p* < 0.00	**0.00**
Did not try accessing	1,016	75.8	165	52.9	851	82.7		**0.00**
Emergency Shelter	1,342		313	23.32	1,029	76.68		
Tried accessing	320	23.9	148	47.3	172	16.7		**0.00**
Yes barriers			46	14.	10	01.0	172.4 (2)	**0.00**
No barriers			102	32.6	162	15.7	*p* < 0.00	**0.00**
Did not try accessing	1,022	76.1	165	52.7	857	83.3		**0.00**
Child Protection Services	1,342		312	23.3	1,026	76.7		
Tried accessing	307	22.9	168	44.6	139	16.4	150.5 (2)	**0.00**
Yes barriers			40	12.8	9	0.9	*p* < 0.00	**0.00**
No barriers			99	31.7	159	15.5		**0.00**
Did not try accessing	1,031	77.1	173	55.5	858	83.6		**0.00**

Chi-square tests were used to assess overall differences in access experiences (yes barriers, no barriers, did not try accessing) between Yes IPV and No IPV group for each service type. To explore specific differences, three pairwise two-proportion z-tests were conducted per service

**A Bonferroni correction was applied within each service category to account for multiple comparisons, adjusting the significance threshold to: αadjusted​ = (0.05/3) ​= 0.017

Among those who sought health care, 50.5% of Yes-IPV participants reported encountering barriers, compared to 35.9% of the No-IPV group (*p* < 0.001). Notably, fewer Yes-IPV participants reported seeking health care (22.4%) relative to the No-IPV group (34.4%). For transportation services, 28.4% of the Yes-IPV group experienced barriers, compared to 7.2% of the No-IPV group (*p* < 0.001). Furthermore, while 69.5% of the No-IPV participants did not attempt to access transportation services, only 40.6% of the Yes-IPV group reported the same. Disparities were also evident in access to mental health services. Among those who sought support, 35.4% of the Yes-IPV group reported barriers, in contrast to 7.8% of the No-IPV group (*p* < 0.001). A higher proportion of No-IPV participants (73.5%) did not attempt to access mental health services, while 64.7% of Yes-IPV group did seek such services.

### Informal supports - communication with friends and family during COVID-19 lockdowns

As detailed in Table 3, participants who experienced IPV during the COVID-19 lockdowns (Yes-IPV) were significantly more likely to report decreased communication with both friends and family compared to those who did not experience IPV (No-IPV). Specifically, 50.1% of the Yes-IPV group reported reduced communication with friends, compared to 36.0% of the No-IPV group (*p* < 0.001), while 46.2% reported decreased communication with family, compared to 23.4% of the No-IPV group (*p* < 0.001). In contrast, participants in the No-IPV group were more likely to report no change in communication patterns with friends (40.9% vs. 25.9%) and family (45.8% vs. 21.1%) compared to the Yes-IPV group (*p* < 0.001). Overall, 60.2% of Yes-IPV participants experienced decreased communication with either friends or family, compared to 38.3% of No-IPV participants (*p* < 0.001).

**Table 3:** Changes in communication with friends and family during COVID-19 lockdowns in Ontario by IPV experience

Variable	Total		YES IPV		NO IPV		χ² (df)/Fisher’s Exact test (df)	Pairwise comparison Bonferroni Correction*
	N	%	N	%	N	%		*p*-value
Communication with Friends during COVID-19 lockdowns	1,343		313		1,030			
Increased	313	23.3	75	24.0	238	23.1	26.7 (2)	0.75
Decreased	528	39.3	157	50.1	371	36.0	*p* < 0.00	**0.00**
No change	502	37.4	81	25.9	421	40.9		**0.00**
Communication with Family during COVID-19 lockdowns	1,342		312		1,030			
Increased	418	31.2	102	32.7	316	30.7	79.4 (2)	0.50
Decreased	386	28.8	144	46.2	242	23.4	*p* < 0.00	**0.00**
No change	538	10.1	66	21.1	472	45.8		**0.00**
Composite Communications with family & friends	1,344		314		1,030			
Did not decrease	760	57.6	125	39.8	635	61.7	46.7 (1)	**0.00**
Decreased	584	43.4	189	60.2	395	38.3	*p* < 0.00	**0.00**

*A Bonferroni correction was applied to adjust for multiple comparisons within each behaviour category. The adjusted significance threshold (αadjusted) was calculated as 0.05/number of pairwise tests per behaviour. Since three pairwise comparisons were conducted for each behaviour (Increased vs. No IPV, Decreased vs. No IPV, No Change vs. No IPV), the adjusted significance level was: adjusted​ = (0.05/3) ​= 0.017

### Multivariate logistic regression models

Multivariate logistic regression analyses (Table 4) identified significant predictors of barriers to formal support services and reduced communication with informal support networks during Ontario’s COVID-19 lockdowns. Multicollinearity was assessed using Variance Inflation Factor (VIF), with all predictor variables within acceptable limits (mean VIF = 2.03).

**Table 4:** Logistic regression analysis of predictors of formal and informal supports during COVID-19 lockdowns in Ontario

**Variables (reference group)**	Faced more than two barriers accessing*				Decreased Communication with family & Friends*			
	Formal Supports				Informal Supports			
	**AOR**	**Stand. Error**	**95% CI**	***p*** **-value**	**AOR**	**Stand. Error**	**95% CI**	***p*** **-value**
IPV* (no IPV)	3.41***	(0.793)	2.161–5.378	0.000	1.57*	(0.312)	1.059–2.314	0.025
Relationship Type (Heterosexual)								
LGBTQ2+	0.90	(0.576)	1.510–3.853	0.000	0.96	(0.197)	0.641–1.436	0.841
Participant Characteristics								
Gender (man)								
Women	0.90	(0.216)	0.561–1.441	0.659	1.09	(0.218)	0.732–1.609	0.685
Gender Diverse & Prefer not to say^#^	0.90	(2.873)	1.054–16.15	0.042	0.29	(0.191)	0.0783–1.057	0.061
Age (55+)								
18–34	1.03	(0.504)	0.392–2.689	0.957	0.52	(0.184)	0.259–1.041	0.065
35–54	1.09	(0.434)	0.497–2.378	0.835	0.91	(0.235)	0.550–1.511	0.720
Race (White)								
Racialized/Indigenous	1.32	(0.373)	0.756–2.294	0.331	1.14	(0.246)	0.742–1.735	0.560
Immigrant & non-permanent resident (non-immigrant)	0.89	(0.369)	0.397–2.009	0.785	0.98	(0.320)	0.517–1.857	0.951
Education (Primary/Secondary)								
Trade/Diploma	0.79	(0.227)	0.449–1.389	0.412	1.09	(0.220)	0.733–1.617	0.675
University	0.75	(0.241)	0.400–1.409	0.372	1.37	(0.300)	0.893–2.106	0.149
Participant Unemployed (employed)	0.63	(0.153)	0.391–1.014	0.057	1.13	(0.183)	0.824–1.555	0.443
Partner Characteristics								
Partner Gender (Man)								
Woman	0.95	(0.221)	0.599–1.496	0.813	1.33	(0.261)	0.905–1.955	0.146
Gender Diverse & Prefer not to say	2.61	(2.222)	0.489–13.87	0.262	5.35*	(4.160)	1.168–24.55	0.031
Partner Age(55+)								
18–34	1.99	(0.984)	0.752–5.245	0.166	1.75	(0.624)	0.874–3.521	0.114
35–54	1.44	(0.578)	0.653–3.162	0.367	1.24	(0.311)	0.755–2.024	0.399
Partner Race (White)								
Racialized/Indigenous	0.97	(0.275)	0.554–1.691	0.910	0.75	(0.160)	0.492–1.139	0.176
Immigrant & non-permanent resident (non-immigrant)	1.28	(0.465)	0.627–2.606	0.500	0.89	(0.258)	0.500–1.569	0.678
Partner Education (Primary/Secondary)								
Trade/Diploma	1.06	(0.280)	0.627–1.776	0.840	1.06	(0.191)	0.741–1.507	0.762
University	0.97	(0.306)	0.518–1.796	0.910	0.83	(0.175)	0.548–1.254	0.374
Partner Unemployed (employed)	0.89	(0.202)	0.572–1.391	0.615	1.11	(0.174)	0.817–1.509	0.503
Household Characteristics								
Income (100,000+)								
<$40,0000	2.82***	(0.841)	1.572–5.059	0.001	0.91	(0.198)	0.593–1.394	0.662
$40,000-$69,999	1.26	(0.350)	0.729–2.169	0.411	0.98	(0.182)	0.684–1.415	0.930
$70,0100-$99,999	1.00	(0.286)	0.569–1.752	0.996	0.83	(0.152)	0.576–1.186	0.301
Geography (Toronto/GTA/905^+^)								
Eastern Ontario	1.33	(0.358)	0.787–2.257	0.286	1.04	(0.202)	0.707–1.520	0.854
Central Ontario	1.43	(0.505)	0.718–2.857	0.308	1.07	(0.275)	0.645–1.770	0.798
Western Ontario	0.74	(0.193)	0.444–1.235	0.249	1.17	(0.191)	0.849–1.612	0.337
Northern Ontario	1.23	(0.442)	0.606–2.487	0.569	0.94	(0.253)	0.552–1.589	0.807
Community Violence (no)								
Yes	1.74**	(0.358)	1.165–2.608	0.007	1.29	(0.195)	0.958–1.732	0.094
No information (enough information)	1.21	(0.241)	0.814–1.783	0.352	1.17	(0.170)	0.879–1.556	0.284
High/Moderate Substance Use Impact (Low)	1.24	(0.298)	0.774–1.987	0.372	1.60*	(0.313)	1.091–2.348	0.016
Children (no children)	0.76	(0.194)	0.460–1.254	0.282	1.31	(0.257)	0.888–1.919	0.175
Caregiver (not a caregiver)	2.75***	(0.688)	1.685–4.491	0.000	0.90	(0.163)	0.635–1.288	0.577
Worse Perceived Physical Health (Better/no change)	1.65*	(0.382)	1.052–2.600	0.029	1.96***	(0.318)	1.430–2.698	0.000
Worse Perceived Mental Health (Better/no change)	1.22	(0.292)	0.761–1.949	0.410	1.76***	(0.270)	1.300–2.373	0.000
Faced > 2 barriers accessing formal services (2 or less)			-		1.50*	(0.292)	1.021–2.192	0.039
Decreased Communication (Increased/stayed same)	1.49*	(0.303)	1.001–2.222	0.049			-	
Constant	0.02***	(0.0103)	0.008–0.055	0.000	0.23***	(0.0746)	0.125–0.437	0.000
Observations	1,146				1,146			

* Unadjusted ORs (not shown in table): IPV was significantly associated with ≥ 2 access barriers (OR = 9.07, 95% CI: 6.62–12.42, *p* < 0.001) and with decreased communication (OR = 2.43, 95% CI: 1.88–3.15, *p* < 0.001). Adjusted results are presented above

#Gender Diverse includes transgender, gender non-conforming, trans woman, trans man, gender fluid. Due to small sample size, we used Fisher’s exact test to determine significance between experience of IPV, and those who did not experience IPV. The small sample size for this category limits the robustness of the results and will be interpreted with caution

##LGBTQ2 + Relationship includes Lesbian, Gay, Bisexual, Transgender, Queer, Two-Spirit, and other orientations (pansexual, asexual)

The formal supports model explained approximately 29% of the variance (Pseudo R² = 0.2833), while the informal supports model explained 10% (Pseudo R² = 0.1003). + Toronto/GTA/905 include the downtown Toronto region, along with the Greater Toronto Area

Standard error in parentheses; *** *p* < 0.001, ** *p* < 0.01, * *p* < 0.05

IPV survivors had significantly higher odds (aOR = 3.4, 95% CI 2.16–5.38; *p* < 0.001) of experiencing more than two access barriers. Participants reporting decreased communication with informal supports were 1.5 times more likely to face these barriers. Additional risk factors included household income under $40,000 (aOR = 2.8; 95% CI 1.57–5.06, *p* = 0.001), being an informal caregiver (aOR = 2.8; 95% CI 1.69–4.49, 0.000), perceiving community violence as a problem (aOR = 1.4; 95% CI 1.17–2.61, *p* = 0.007), and reporting worsened physical health (aOR = 1.7; 95% CI 1.05–2.60, *p* = 0.029).

IPV was also associated with reduced communication with family and friends (aOR = 1.6; 95% CI 1.06–2.31, *p* = 0.025). Participants with gender diverse partners or those who preferred not to disclose gender had 5-fold increased odds of decreased communication with informal supports. Further disaggregation (see Additional file 3) revealed the reduced informal support was primarily driven by decreased communication with family (aOR = 7.5, 95% CI 1.66–33.89, *p* = 0.009) rather than with friends (aOR = 1.82, 95% CI 0.45–7.26, *p* = 0.398).

Substance use impact was another predictor, moderate/high levels associated with a 60% increase in odds (*p* = 0.016). Poorer perceived mental health (aOR = 1.76; 95% CI 1.30–2.37, *p* < 0.001) and physical health (aOR = 1.96; 95% CI 1.43–2.70, *p* = 0.000) were also significant predictors., Notably, individuals experiencing more than two barriers to formal supports were 1.5 times (*p* = 0.039) more likely to report decreased informal support communication.

Exploratory mediation analyses revealed that both mental and physical health partially mediated the relationship between IPV and decreased communication with informal supports (see Additional file 3). However, only physical health partially mediated the link between IPV and formal support barriers; mental health was not a significant mediator. IPV remained a robust predictor across all models.

## Discussion

Despite growing evidence of increased intimate partner violence (IPV) during the COVID-19 pandemic, few studies have directly compared the support access experiences of IPV survivors and non-survivors. Nearly one in four participants (23.4%) in this Ontario-based sample self-reported experiencing IPV during the pandemic lockdowns, a higher prevalence than reported in comparable studies, such as Shyrokonis et al. (2024), which found a 14.8% IPV rate in Michigan reported during the initial phase of the pandemic (March to June 2020) [53]. The broader timeframe captured in the current study (March 2020 to June 2021) may account for this difference, as extended lockdown periods and recurring waves of restrictions likely exacerbated conditions that contribute to IPV. While national Canadian surveys have estimated lifetime IPV exposure 44% for women and 36% for men, and 12-month prevalence at 12% and 6%, respectively, these data do not isolate pandemic-specific trends [2]. Police-reported IPV rates in Ontario increased slightly from 242 per 100,000 in 2019 to 249 per 100,000 in 2021 [54]; these reports are widely understood to underestimate the true prevalence due to under-reporting and systemic barriers [55]. While these sources provide valuable insights into IPV prevalence, they do not capture the lived experiences of IPV survivors during the COVID-19 pandemic. By examining participants’ self-reported experiences during the lockdowns and their access to a range of formal and informal services, this study offers new insight into how COVID-19 lockdowns may have shaped IPV survivors’ experiences and their ability to access a range of formal and informal services, regardless of whether those services were IPV-specific.

Findings of this study support the hypothesis that IPV survivors faced disproportionately greater challenges accessing formal and informal supports during COVID-19 lockdowns. Survivors were three times more likely to face barriers to formal support services (e.g., health care, mental health, transportation) and 1.6 times more likely to report reduced communication with friends and family. While limited service access affected many during lockdowns, IPV survivors encountered compounding barriers – such as isolation with the perpetrator and competing survival demands (such as employment, homeschooling, and caregiving) [38]. These findings highlight the need to address the barriers that IPV survivors face in accessing a broad range of services during periods of social disruption and isolation — including, but not limited to, IPV-specific supports. Although service use may be lower among non-IPV individuals, among all those who sought help, IPV survivors faced consistently greater obstacles. These findings align with recent literature demonstrating that crisis-responsive support systems for IPV survivors must go beyond universal access - survivor-centred, trauma-informed, and culturally competent models are essential, especially during emergencies [38].

This study found that men (48.7%) and women (48.1%) reported experiencing IPV in nearly equal proportions. These results do not support our hypothesis that women or gender-diverse individuals were at higher risk of IPV or more likely to face service barriers. Several factors may explain this finding. The nature of our IPV measurement may have influenced these findings, as our broad, behaviour-specific assessment captured multiple forms of IPV (psychological, economic, technological, physical, and sexual) rather than focusing on severity-weighted measures or specific types of violence that may show greater gender disparities. Self-selection bias could have influenced participation, with individuals affected by IPV - regardless of gender - more likely to take part. Men may be more likely to report certain types of IPV (such as psychological or economic abuse), compared to severe physical violence [56], potentially explaining the similar prevalence rates observed with our comprehensive IPV measure. The use of an online panel may have introduced sample-specific characteristics, such as greater representation of bidirectional IPV. Anonymity may also have reduced stigma and enabled men to disclose experiences more openly [57]. These measurement considerations highlight the importance of understanding how different assessment approaches may capture varying thresholds and types of IPV experiences across gender groups.

While these findings do not negate the gendered nature of coercive IPV, they highlight the complexity of measuring and understanding IPV experiences across different populations. Since the 1970s, IPV literature has emphasized patriarchal control of women [58]. Bates and Taylor (2019) highlight persistent academic divisions between feminist and family violence researchers, which they argue detract from evidence-based practice. Hine (2019) similarly critiques the gender paradigm, which reinforces the binary narrative of violent men and victimized women [59]. Our findings, while limited by broad measurement approaches, contribute to the ongoing discussions about how different assessment methods may capture varying experiences and types of IPV across gender groups. Further research using more specific, validated measures is needed to better understand potential gender differences in IPV experiences and access to support services during crisis periods like COVID-19. A nuanced approach to measurement and interpretation is essential to ensure that research findings appropriately inform support services for all survivors [58].

This study also found that a higher proportion of IPV survivors were themselves gender diverse or had partners who identified as gender diverse or preferred not to disclose their gender than individuals who did not experience IPV. After controlling for all variables, these partners were significantly more likely to report reduced communication with informal supports, particularly with family. Literature suggests that transgender individuals face elevated communication apprehension and loneliness, which can reduce social interaction [60]. Many experience limited familial support due to rejection or misunderstanding, negatively impacting mental health [61]. Conversely, peer support – especially from friends and online communities - can foster resilience [62].​ These dynamics highlight the importance of understanding variation in informal support patterns among gender-diverse populations. Due to small sample size in this category, findings should be interpreted with caution but underscore the need for further investigation.

Lower household income (<$40,000CAD) was more common among IPV survivors and independently predicted greater barriers to formal support service access (3X higher odds). Cotter (2021) suggests that IPV may contribute to income reduction, rather than income necessarily predisposing individuals to IPV, particularly for experiences reported within the past 12 months [63]. A 2024 scoping review identified significant access barriers for low-income individuals, including transportation issues, appointment scheduling difficulties, and internalized stigma related to poverty and marginalization [64].

Perceptions of community violence were also associated with reduced access to formal supports. Neighbourhood violence has been linked to chronic stress, depression, poor physical health, fear, isolation, and diminished trust, all of which limit service access [65]. Fear of victimization, reduced mobility, lack of service infrastructure, and avoidance of unsafe areas further compound these barriers [65]. COVID-19 lockdowns amplified these effects through increased isolation and disrupted services [38]. Participants who identified community violence as a concern were twice as likely to face barriers, and a higher proportion of IPV survivors reported such concerns – suggesting layered vulnerabilities and heightened health risks.

Substance use also emerged as a key factor. A much higher proportion of IPV survivors (61.5%) reported moderate to high substance use impact in their relationships, compared to the non-IPV (8.9%). Controlling for other variables, participants with greater substance use-related relationship strain were 60% more likely to report reduced communication with family and friends. Substance use can erode communication, fuel aggression, and restrict survivors’ ability to seek help [66–68]. Survivors may withdraw or be prevented from reaching out, while friends and family may disengage due to frustration or helplessness [69]. This results in deepened isolation and diminished support.

Caregiving responsibilities also influenced access to support for IPV survivors. Participants caring for a child and/or other family member were nearly three times more likely to face formal service access barriers. Caregivers often prioritize others’ needs over their own, struggle with time constraints, and encounter delays due to mental and physical strain, long waitlists, and service costs [70–72]. During COVID-19, these challenges intensified due to reduced service availability and increased caregiving demands [73]. A higher proportion of IPV survivors were caregivers, and recent research suggests that caregiving during lockdowns exacerbated isolation and stress for this group [38].

Participants with worse perceived physical health were more likely to report barriers to formal and informal supports; those with worse mental health were more likely to report reduced informal support only. IPV survivors reported poorer health than non-IPV participants. These findings align with pandemic-era research showing increased caregiving burdens, virtual schooling, employment disruptions, isolation, and limited access to care [38]. For IPV survivors, threats to safety were heightened by lockdowns, perpetrator proximity, and reduced access to resources, compounding the risk of poor physical and mental health outcomes [74]. Exploratory mediation analyses revealed that both mental and physical health partially mediated the relationship between IPV and decreased communication with informal supports, while only physical health served as a partial mediator for formal support barriers. These findings suggest that the health impacts of IPV may represent one pathway through which survivors experience reduced access to support systems. However, IPV remained a robust independent predictor across all models, indicating that IPV’s effects on support access extend beyond health-related mechanisms alone. This highlights the importance of addressing both the direct barriers IPV creates (such as perpetrator control and isolation) and the indirect pathways through compromised health status.

### Implications for policy, interventions, and future research

The COVID-19 pandemic intensified pre-existing barriers to formal and informal supports, underscoring the need to maintain accessible services during crises. As IPV survivors were more likely to encounter service barriers and reduced communication with informal networks, policies should prioritize trauma-informed, accessible services for this population during public health emergencies and disasters. Policies and programs must also consider intersecting factors, including caregiving responsibilities, low-income, and exposure to community violence.

The unintended consequences of COVID-19 lockdowns on IPV survivors exposed the reactive and unsustainable nature of IPV sector funding and resources [38]. Rather than debating the use of lockdowns, future emergency planning must proactively include IPV survivors and ensure sustained funding and resources to guarantee access to supports. Several innovative approaches emerged during COVID-19 to support IPV survivors, including the use of technology to reduce transportation barriers, discreet safety strategies (e.g., code words via text), and safe spaces at banks, pharmacies, and online platforms [38]. If proven effective, these public health strategies should be integrated into future crisis response plans.

Further research should explore how IPV during the pandemic contributed to negative health outcomes and inequities. While this study offers quantitative insights, qualitative research is needed to deepen understanding of IPV survivors’ lived experiences and their perceptions of service access and well-being. Including both survivor and service provider perceptions could reveal structural and personal barriers that impacted access and outcomes. Given that nearly 50% of IPV survivors in this study identified as men, future work should examine their unique experiences, including stigma and service barriers. Although the literature often focuses on men as perpetrators and women as survivors, more research is needed on the specific needs and challenges of male-identified survivors, especially in crisis contexts.

### Strengths

The study’s large sample size (*n* = 1,344) enhances statistical power and strengthens the ability to detect associations that exist. Through multivariate logistic regression, the analysis identified key predictors of barriers to accessing formal supports and decreased communication with informal supports, while controlling for confounding variables. The selection of covariates was informed by a comprehensive literature review and consultations with IPV community experts. This research offers timely and relevant insights into the impact of COVID-19 lockdowns on IPV survivors’ access to support, highlighting crisis-specific challenges. To the authors’ knowledge, this is the first study to examine the impact of IPV on both formal and informal support barriers during the pandemic by comparing data from IPV survivors with those who did not experience IPV.

While the study focused on Ontario-specific data, the findings can offer valuable insights for other provinces or countries that implemented similar COVID-19 lockdown mandates. The unique challenges of pandemic-related isolation, lack of access to services, and increased IPV risk may also inform experiences during other crises, such as environmental disasters or economic downturns, where similar conditions arise [38]. Finally, while this study was pandemic-specific, the insights into barriers to accessing support and the impact of isolation can inform crisis response strategies in diverse emergency contexts.

### Limitations

The study’s cross-sectional design limits the ability to draw causal inferences between IPV and access to services. While significant associations were identified, causality and directionality of these relationships cannot be confirmed [75]. Small sample sizes for certain subgroups, such as gender-diverse individuals and specific racial or ethnic groups, also limit interpretation and highlighting the need for further targeted research. Additionally, while our covariate selection was guided by existing literature and our conceptual framework, we recognize that some variables treated as confounders—such as income, education, and caregiving—may also act as moderators. This possibility reflects the complexity of IPV-related experiences and underscores the need for future research to explore potential interaction effects.

Self-reported data introduce potential biases, including recall bias and social desirability bias [76, 77]. Participants may have underreported sensitive experiences such as IPV, mental health issues, or substance use, due to stigma. To mitigate this, the survey was fully anonymous with no identifying information collected. In addition, the outcome measures for facing barriers to formal supports and decreased communication with family and friends (informal supports) were based on self-reported, dichotomous items rather than validated scales, which does not fully capture the complexity of support access.

Selection bias is another concern. Reliance on online surveys distributed electronically through a research firm may exclude individuals without internet access or digital literacy - disproportionately affecting marginalized groups, such as older adults, low-income households, and rural residents [78]. Survey firms often draw from existing panels, which tend to overrepresent individuals with higher income and education levels [79]. Voluntary participation may have also skewed the sample, with individuals more comfortable sharing their experiences — such as those facing less severe IPV or who had access to support — overrepresented, while those experiencing more severe IPV or resource scarcity remain underrepresented [80].

Non-response bias also likely influenced the results. Those facing severe IPV, lacking digital access, or distrusting formal institutions may have opted out [80]. Marginalized groups, including racialized individuals and immigrants, may have hesitated to participate due to confidentiality concerns or fear of reprisal. Although the survey was available in both English and French, non-official language speakers, such as recent immigrants or non-permanent residents, were likely excluded. Hard-to-reach populations – such as those experiencing homelessness, precarious housing, or substance use issues - were also likely underrepresented. These individuals, often the most impacted by IPV, may have faced confidentiality concerns, discouraging full disclosure or contributing to incomplete responses.

Our sample was comprised of self-identified survivors of IPV, which may have excluded individuals who experienced IPV but do not conceptualize or label their experiences as such. While participants completed behaviorally-specific measures of IPV after initial screening, the initial eligibility criteria required individuals to self-identify as having experienced IPV based on a statement naming types of IPV (emotional, sexual, physical, mental, financial, coercive, exploitative, spiritual and technology-based abuse). Research has shown that some IPV survivors may not self-identify as such, even when their experiences meet objective criteria for IPV [81, 82]. Factors such as normalization of violence, cultural contexts, shame, or unfamiliarity with IPV terminology can influence self-identification [83], potentially introducing selection bias by excluding IPV survivors who do not recognize their experiences using conventional (non-behaviourally specific) IPV terminology.

## Conclusion

This study demonstrates a strong association between IPV experience and barriers to both formal and informal supports during the COVID-19 pandemic in Ontario. Survivors were significantly more likely to face obstacles when accessing essential services and maintaining social support networks. These findings point to critical gaps in public health and social service systems in ensuring equitable support access for IPV survivors. Strengthening system responsiveness - through proactive, trauma-informed, and inclusive strategies - is necessary not only to safeguard immediate safety and well-being but to improve long-term health outcomes. Policymakers, service providers, and health care professionals must integrate these insights into emergency preparedness to promote resilience and health equity for at-risk populations in future crises.

## Supplementary Information

Supplementary Material 1: Additional file 1: Sampling by Gender in Ontario

Supplementary Material 2: Additional file 2: STATA Syntax for data analysis. 

Supplementary Material 3: Additional file 3: Additional analysis (sub-analysis and exploratory mediation analysis). 

Supplementary Material 4: Additional file 4: STROBE Checklist.

## Data Availability

The datasets generated and/or analysed during the current study are not publicly available due to the sensitive nature of the data, which includes information on experiences of intimate partner violence, but are available from the first author and corresponding author upon reasonable request.

## Abbreviations

IPV: Intimate Partner Violence
COVID-19: Coronavirus Disease 2019
WHO: World Health Organization
SLE: Stressful Life Events
VAW: Violence Against Women
CI: Confidence Interval
AOR: Adjusted Odds Ratio
GTA: Greater Toronto Area
LGBTQ2+: Lesbian, Gay, Bisexual, Transgender, Queer, Two-Spirit and other identities
REB: Research Ethics Board
CIHI: Canadian Institute for Health Information
STATA: Statistics/Data Analysis (statistical software)
