# Supplementary material for: Locked down, locked out: a cross-sectional study on experiences of intimate partner violence (IPV) and barriers to formal and informal support during COVID-19 lockdowns in Ontario
Source: BMC Public Health. 2025 Nov 17;25:3962. doi: 10.1186/s12889-025-25124-7 (PMC12621413; doi:10.1186/s12889-025-25124-7)
Supplement: Supplementary file 1 — Supplementary Material 1: Additional file 1: Sampling by Gender in Ontario [file 12889_2025_25124_MOESM1_ESM.pdf]

gender	Sample start	Distribution	NB sent	NB open	% Answer rep	COMPLETE	COMPLETE %	Incomplete	SCREENED	QUOTAS	Incidence
F	13642	50.18%	13434	1600	11.91%	661	49.40%	136 (4.44%)	803		45.15%
M	13542	49.82%	13056	1472	11.27%	677	50.60%	112 (3.95%)	683		49.78%
Total:	27184	100%	26490	3072	11.60%	1338	1338	248	1486	0	47.38%
