# Supplementary material for: Locked down, locked out: a cross-sectional study on experiences of intimate partner violence (IPV) and barriers to formal and informal support during COVID-19 lockdowns in Ontario
Source: BMC Public Health. 2025 Nov 17;25:3962. doi: 10.1186/s12889-025-25124-7 (PMC12621413; doi:10.1186/s12889-025-25124-7)
Supplement: Supplementary file 2 — Supplementary Material 2: Additional file 2: STATA Syntax for data analysis. [file 12889_2025_25124_MOESM2_ESM.pdf]

```
*************************
*CREATING CODEBOOK*
*************************

*DROPPING CASES*
label variable DROP "Cases to Drop"
label define casestodrop 1 "DROP" 0 "Keep"
label values DROP casestodrop
codebook DROP

*Language of Survey*
label variable LANG "Language the Survey was taken"
label define langtaken 1 "English" 2 "French"
label values LANG langtaken
codebook LANG

*EXPERIENCE OF IPV*
label variable IPV_EXP "Experience of IPV During COVID"
label define IPV 1 "YesIPV" 2 "NoIPV"
label values IPV_EXP IPV
codebook IPV_EXP

*HEALTH BEFORE*
label variable PHYS_B4_COVID "Physical_Health_B4_COVID"
label define PHYSB4 1 "Excellent" 2 "Very good" 3 "Good" 4 "Fair" 5 "Poor" 0 "Missing"
label values PHYS_B4_COVID PHYSB4
codebook PHYS_B4_COVID

label variable MENTAL_B4_COVID "Mental_Health_B4_COVID"
label define MENTB4 1 "Excellent" 2 "Very good" 3 "Good" 4 "Fair" 5 "Poor" 0 "Missing"
label values MENTAL_B4_COVID MENTB4
codebook MENTAL_B4_COVID

*HEALTH DURING*
label variable PHYS_IN_COVID "Physical_Health_IN_COVID"
label define PHYSIN 1 "MuchBetter" 2 "Better" 3 "About the Same" 4 "Worse" 5 "MuchWorse" 0 "Missing"
label values PHYS_IN_COVID PHYSIN
codebook PHYS_IN_COVID

label variable MENTAL_IN_COVID "Mental_Health_IN_COVID"
label define MENTIN 1 "MuchBetter" 2 "Better" 3 "About the Same" 4 "Worse" 5 "MuchWorse" 0 "Missing"
label values MENTAL_IN_COVID MENTIN
codebook MENTAL_IN_COVID

```

```
*HEALTH POST*
label variable PHYSICAL_POST "Physical_Health_POST_COVID"
label define PHYSPOST 1 "MuchBetter" 2 "Better" 3 "About the Same" 4 "Worse" 5 "MuchWorse" 0 "Missing"
label values PHYSICAL_POST PHYSPOST
codebook PHYSICAL_POST

label variable MENTAL_POST "Mental_Health_POST_COVID"
label define MENTPOST 1 "MuchBetter" 2 "Better" 3 "About the Same" 4 "Worse" 5 "MuchWorse" 0 "Missing"
label values MENTAL_POST MENTPOST
codebook MENTAL_POST


*HEALTH BEHAVIOURS DURING*
label variable ALCOHOL_DURING "ConsumingAlcoholDuringCOVID"
label define ALCOHinCOV 1 "increased" 2 "decreased" 3 "no change" 0 "Missing"
label values ALCOHOL_DURING ALCOHinCOV
codebook ALCOHOL_DURING

label variable TOBAC_DRUING "ConsumingTobaccoDuringCOVID"
label define TOBACinCOV 1 "increased" 2 "decreased" 3 "no change" 0 "Missing"
label values TOBAC_DRUING TOBACinCOV
codebook TOBAC_DRUING

label variable CANNAB_DURING "ConsumingCannabisDuringCOVID"
label define CANNABinCOV 1 "increased" 2 "decreased" 3 "no change" 0 "Missing"
label values CANNAB_DURING CANNABinCOV
codebook CANNAB_DURING

label variable SUBSTANCE_DURING "ConsumingSubstancesDuringCOVID"
label define SUBSTinCOV 1 "increased" 2 "decreased" 3 "no change" 0 "Missing"
label values SUBSTANCE_DURING SUBSTinCOV
codebook SUBSTANCE_DURING

label variable TV_DURING "WatchingTVDuringCOVID"
label define TVinCOV 1 "increased" 2 "decreased" 3 "no change" 0 "Missing"
label values TV_DURING TVinCOV
codebook TV_DURING

label variable INTERNET_DURING "TimeOnInternetDuringCOVID"
label define INTERinCOV 1 "increased" 2 "decreased" 3 "no change" 0 "Missing"
```

```
label values INTERNET_DURING INTERinCOV
codebook INTERNET_DURING

label variable EXERCISING_DURING "ExercisingDuringCOVID"
label define EXERCinCOV 1 "increased" 2 "decreased" 3 "no change" 0 "Missing"
label values EXERCISING_DURING EXERCinCOV
codebook EXERCISING_DURING

label variable EATJUNK_DURING "EatingJunkDuringCOVID"
label define EATJUNKinCOV 1 "increased" 2 "decreased" 3 "no change" 0 "Missing"
label values EATJUNK_DURING EATJUNKinCOV
codebook EATJUNK_DURING

label variable COMM_FRND_DURING "CommunicatingFriendsDuringCOVID"
label define COMMFRNDinCOV 1 "increased" 2 "decreased" 3 "no change" 0 "Missing"
label values COMM_FRND_DURING COMMFRNDinCOV
codebook COMM_FRND_DURING

label variable COMM_FAM_DURING "CommunicatingFamilyDuringCOVID"
label define COMMFAMinCOV 1 "increased" 2 "decreased" 3 "no change" 0 "Missing"
label values COMM_FAM_DURING COMMFAMinCOV
codebook COMM_FAM_DURING

label variable INFORMAL_DURING "SeekingINFORMALSupportsDuringCOVID"
label define INFORSUPinCOVID 1 "increased" 2 "decreased" 3 "no change" 0 "Missing"
label values INFORMAL_DURING INFORSUPinCOVID
codebook INFORMAL_DURING

label variable FORMAL_DURING "SeekingFORMALSupportsDuringCOVID"
label define FORMSUPinCOVID 1 "increased" 2 "decreased" 3 "no change" 0 "Missing"
label values FORMAL_DURING FORMSUPinCOVID
codebook FORMAL_DURING

label variable SLEEP_DURING "QualityOfSleepDuringCOVID"
label define SLEEPCOVID 1 "increased" 2 "decreased" 3 "no change" 0 "Missing"
label values SLEEP_DURING SLEEPCOVID
codebook SLEEP_DURING

*CAREGIVING AND CHILDREN*

label variable PRIMARY_CAREGIVER_DURING "Primary_CaregiverDuringCOVID"
```

```
label variable PRIMARY_CAREGIVER_DURING "Primary_CaregiverDuringCOVID"
label define PRIMARY_CAREGIVER_DURING 1 "Yes" 2 "No" 0 "Missing"
label values PRIMARY_CAREGIVER_DURING CAREGIVINGCOV
codebook PRIMARY_CAREGIVER_DURING

label variable NUM_CHILD "NumberOfChildren_under_17"
label define NumCHILD17 1 "None" 2 "1" 3 "2" 4 "3" 5 "4" 6 ">4"
label values NUM_CHILD NumCHILD17
codebook NUM_CHILD

label variable CAREGIVING_nonCHILD "Primary_CaregiverNon_Child_DuringCOVID"
label define CAREGIVINGNONCHILD 1 "Yes" 2 "No" 0 "Missing"
label values CAREGIVING_nonCHILD CAREGIVINGNONCHILD
codebook CAREGIVING_nonCHILD

label variable CHANGE_CAREGIVING_DURING "ChangeInCaregivingDuringCOVID"
label define CAREGIVCHANGEINCOV 1 "No_stayed_same" 2 "Yes_increased" 3 "Yes_decreased" 0 "Missing"
label values CHANGE_CAREGIVING_DURING CAREGIVCHANGEINCOV
codebook CHANGE_CAREGIVING_DURING

*INCOME/JOBSTATUS CHANGE DURING COVID*

label variable JOB_STATUS_DURING  "Job_status_changeINCOVID"
label define JOBSTATUSCOV 1 "Yes_no_longer_job" 2 "No_kept_job" 0 "Missing"
label values JOB_STATUS_DURING JOBSTATUSCOV
codebook JOB_STATUS_DURING

label variable INCOME_DURING  "ChangIncomeDuringCOVID"
label define INCOMECHANGE 1 "Yes_decreased" 2 "No_stayed_same" 3 "Yes_increased" 0 "Missing"
label values INCOME_DURING INCOMECHANGE
codebook INCOME_DURING

*FACE TO FACE CONTACT VS VIRTUAL*

label variable F2F_CONTACT_DURING  "Face_to_Face_Contact_during_COVID"
label define F2FinCOV 1 "No_Contact_outside_HH" 2 "Yes_1to3_Individ" 3 "Yes_4to8_Individ" 4 "Yes_>8" 5 "Unknown"
label values F2F_CONTACT_DURING F2FinCOV
codebook F2F_CONTACT_DURING

label variable VIRT_CONTACT_DURING  "Virtual_Contact_during_COVID"
label define VIRTinCOV 1 "No_Contact_outside_HH" 2 "Yes_1to3_Individ" 3 "Yes_4to8_Individ" 4 "Yes_>8" 5 "Unknown"
```

```
label define VIRTinCOV 1 "No_Contact_outside_HH" 2 "Yes_1to3_Individ" 3 "Yes_4to8_Individ" 4 "Yes_>8" 5 "Unknown"
label values VIRT_CONTACT_DURING VIRTinCOV
codebook VIRT_CONTACT_DURING

*SERVICE IN PERSON OR VIRTUAL OR BOTH*

label variable SERVICES_VIRT_INPERS  "Services accessed in person, virtual or both"
label define SERVICEPERSONVIRT 1 "in-person" 2 "Virtual" 3 "both" 4 "DidNotAccessDuringLockdown"
label values SERVICES_VIRT_INPERS SERVICEPERSONVIRT
codebook SERVICES_VIRT_INPERS

*BARRIERS DURING COVID*

label variable BARR_COMMBASE_DURING "Barr_CommunityBasedOrg_DURING_COVID"
label define BARRCBOCOV 1 "Yes" 2 "No" 3 "Did_not_try" 0 "Missing"
label values BARR_COMMBASE_DURING BARRCBOCOV
codebook BARR_COMMBASE_DURING

label variable BARR_COUNS_DURING  "Barr_Counselling_DURING_COVID"
label define BARRCOUNSCOV 1 "Yes" 2 "No" 3 "Did_not_try" 0 "Missing"
label values BARR_COUNS_DURING BARRCOUNSCOV
codebook BARR_COUNS_DURING

label variable BARR_CRISISLINE_DURING  "Barr_CrisisLine_DURING_COVID"
label define BARRCRLineCOV 1 "Yes" 2 "No" 3 "Did_not_try" 0 "Missing"
label values BARR_CRISISLINE_DURING BARRCRLineCOV
codebook BARR_CRISISLINE_DURING

label variable BARR_HEALTHCR_DURING  "Barr_Healthcare_DURING_COVID"
label define BARRHCCOV 1 "Yes" 2 "No" 3 "Did_not_try" 0 "Missing"
label values BARR_HEALTHCR_DURING BARRHCCOV
codebook BARR_HEALTHCR_DURING

label variable BARR_EMER_SHELT_DURING "Barr_Emergency_Shelter_DURING_COVID"
label define BARRSHELTERCOV 1 "Yes" 2 "No" 3 "Did_not_try" 0 "Missing"
label values BARR_EMER_SHELT_DURING BARRSHELTERCOV
codebook BARR_EMER_SHELT_DURING

label variable BARR_SETT_DURING  "Barr_Settlement_Agency_DURING_COVID"
label define BARRSETTAGCOV 1 "Yes" 2 "No" 3 "Did_not_try" 0 "Missing"
```

```
label define BARRSETTAGCOV 1 "Yes" 2 "No" 3 "Did_not_try" 0 "Missing"
label values BARR_SETT_DURING BARRSETTAGCOV
codebook BARR_SETT_DURING

label variable BARR_CHILDPROT_DURING "Barr_ChildProtection_DURING_COVID"
label define BARRCHILDPROCOV 1 "Yes" 2 "No" 3 "Did_not_try" 0 "Missing"
label values BARR_CHILDPROT_DURING BARRCHILDPROCOV
codebook BARR_CHILDPROT_DURING

label variable BARR_LEGAL_DURING  "Barr_Legal_DURING_COVID"
label define BARRLEGCOV 1 "Yes" 2 "No" 3 "Did_not_try" 0 "Missing"
label values BARR_LEGAL_DURING BARRLEGCOV
codebook BARR_LEGAL_DURING

label variable BARR_MENT_HC_DURING "Barr_MentalHealth_DURING_COVID"
label define BARRMENTHCOV 1 "Yes" 2 "No" 3 "Did_not_try" 0 "Missing"
label values BARR_MENT_HC_DURING BARRMENTHCOV
codebook BARR_MENT_HC_DURING

label variable BARR_ADDICT_DURING "Barr_AddictionServices_DURING_COVID"
label define BARRADDICTCOV 1 "Yes" 2 "No" 3 "Did_not_try" 0 "Missing"
label values BARR_ADDICT_DURING BARRADDICTCOV
codebook BARR_ADDICT_DURING

label variable BARR_HOUSING_DURING "Barr_HOUSING_DURING_COVID"
label define BARRHOUSCOV 1 "Yes" 2 "No" 3 "Did_not_try" 0 "Missing"
label values BARR_HOUSING_DURING BARRHOUSCOV
codebook BARR_HOUSING_DURING

label variable BARR_TRANS_DURING "Barr_Transportation_DURING_COVID"
label define BARRTRANSPCOV 1 "Yes" 2 "No" 3 "Did_not_try" 0 "Missing"
label values BARR_TRANS_DURING BARRTRANSPCOV
codebook BARR_TRANS_DURING

*Barriers POST COVID*

*label variable BARR_COMMBASE_POST "Barr_CommunityBasedOrg_POST_COVID"
*label define BARRCBOPOST 1 "Yes" 2 "No" 3 "Did_not_try" 0 "Missing"
*label values BARR_COMMBASE_POST BARRCBOPOST
*codebook BARR_COMMBASE_POST
```

```
*codebook BARR_COMMBASE_POST

*label variable BARR_COUNS_POST  "Barr_Counselling_POST_COVID"
*label define BARRCOUNSPOST 1 "Yes" 2 "No" 3 "Did_not_try" 0 "Missing"
*label values BARR_COUNS_POST BARRCOUNSPOST
*codebook BARR_COUNS_POST

*label variable BARR_CRISISLIN_POST  "Barr_CrisisLine_POST_COVID"
*label define BARRCRLinePOST 1 "Yes" 2 "No" 3 "Did_not_try" 0 "Missing"
*label values BARR_CRISISLIN_POST BARRCRLinePOST
*codebook BARR_CRISISLIN_POST

*label variable BARR_HEALTHC_POST  "Barr_Healthcare_POST_COVID"
*label define BARRHCPOST 1 "Yes" 2 "No" 3 "Did_not_try" 0 "Missing"
*label values BARR_HEALTHC_POST BARRHCPOST
*codebook BARR_HEALTHC_POST

*label variable BARR_EMER_SHELT_POST "Barr_Emergency_Shelter_POST_COVID"
*label define BARRSHELTERPOST 1 "Yes" 2 "No" 3 "Did_not_try" 0 "Missing"
*label values BARR_EMER_SHELT_POST BARRSHELTERPOST
*codebook BARR_EMER_SHELT_POST

*label variable BARR_SETT_POST  "Barr_Settlement_Agency_POST_COVID"
*label define BARRSETTAGPOST 1 "Yes" 2 "No" 3 "Did_not_try" 0 "Missing"
*label values BARR_SETT_POST BARRSETTAGPOST
*codebook BARR_SETT_POST

*label variable BARR_CHILDPROT_POST  "Barr_ChildProtection_POST_COVID"
*label define BARRCHILDPROPOST 1 "Yes" 2 "No" 3 "Did_not_try" 0 "Missing"
*label values BARR_CHILDPROT_POST BARRCHILDPROPOST
*codebook BARR_CHILDPROT_POST

*label variable BARR_LEGAL_POST  "Barr_Legal_POST_COVID"
*label define BARRLEGPOST 1 "Yes" 2 "No" 3 "Did_not_try" 0 "Missing"
*label values BARR_LEGAL_POST BARRLEGPOST
*codebook BARR_LEGAL_POST

*label variable BARR_MENTAHC_POST "Barr_MentalHealth_POST_COVID"
*label define BARRMENTHPOST 1 "Yes" 2 "No" 3 "Did_not_try" 0 "Missing"
*label values BARR_MENTAHC_POST BARRMENTHPOST
```

```
*label values BARR_MENTAHC_POST BARRMENTHPOST
*codebook BARR_MENTAHC_POST

*label variable BARR_ADDICT_POST "Barr_AddictionServices_POST_COVID"
*label define BARRADDICTPOST 1 "Yes" 2 "No" 3 "Did_not_try" 0 "Missing"
*label values BARR_ADDICT_POST BARRADDICTPOST
*codebook BARR_ADDICT_POST

*label variable BARR_HOUS_POST "Barr_HOUSING_POST_COVID"
*label define BARRHOUSPOST 1 "Yes" 2 "No" 3 "Did_not_try" 0 "Missing"
*label values BARR_HOUS_POST BARRHOUSPOST
*codebook BARR_HOUS_POST

*label variable BARR_TRANSP_COVID "Barr_Transportation_POST_COVID"
*label define BARRTRANSPOST 1 "Yes" 2 "No" 3 "Did_not_try" 0 "Missing"
*label values BARR_TRANSP_COVID BARRTRANSPOST
*codebook BARR_TRANSP_COVID


*WERE SERVICES HELPFUL*

label variable HELPFUL_DURING "Were_services_Helpful"
label define SERVHELPCOV 1 "Yes" 2 "No" 3 "Did_not_try" 0 "Missing"
label values HELPFUL_DURING SERVHELPCOV
codebook HELPFUL_DURING

*Information during COVID*

label variable INFORMATION_DURING "Information_during_COVID"
label define INFORMinCOVID 1 "Yes" 2 "No" 0 "Missing"
label values INFORMATION_DURING INFORMinCOVID
codebook INFORMATION_DURING

*Accessed BENEFITS DURING COVID**

label variable ACCESS_BENEFIT_DURING "ACCESSED_Financial_Benefits_during_COVID"
label define BENEFITSinCOVID 1 "Yes_applied_unsuccessfull" 2 "Yes_applied_successful" 3 "No_did_not_apply" 0 "Missing"
label values ACCESS_BENEFIT_DURING BENEFITSinCOVID
codebook ACCESS_BENEFIT_DURING

**DEMOGRPHAIC DATA**

*Gender*
label variable GENDER_PARTICIPANT "Participant_Gender"
```

```
label variable GENDER_PARTICIPANT "Participant_Gender"
label define GENDERPART 1 "Woman" 2 "Man" 3 "Gender_Non_Conforming" 4 "Transgender" 5 "Trans Woman" 6 "Trans Man" 7 "Gender Fluid" 8 "PrefNotSay" 0 "Missing"
label values GENDER_PARTICIPANT GENDERPART
codebook GENDER_PARTICIPANT

label variable GENDER_PARTNER "Partner_Gender"
label define GENDERPARTNER 1 "Man" 2 "Woman" 3 "Gender_Non_Conforming" 4 "Transgender" 5 "Trans Woman" 6 "Trans Man" 7 "Gender Fluid" 8 "PrefNotSay" 0 "Missing"
label values GENDER_PARTNER GENDERPARTNER
codebook GENDER_PARTNER

*Age*

label variable AGE_PART "Age_Participant"
label define AGEPARTICIPANT 1 "18-24" 2 "25-34" 3 "35-44" 4 "45-54" 5 "55-64" 6 "65+" 0 "Missing"
label values AGE_PART AGEPARTICIPANT
codebook AGE_PART

label variable AGE_PARTNER "Age_Partner"
label define AGEPARTNER 1 "18-24" 2 "25-34" 3 "35-44" 4 "45-54" 5 "55-64" 6 "65+" 7 "Don't_know" 0 "Missing"
label values AGE_PARTNER AGEPARTNER
codebook AGE_PARTNER

*Citizenship Status*

label variable CITIZEN_STATUS "Participant_citizenship_status"
label define CITIZENPARTICIPANT 1 "Canadian citizen" 2 "landed_imm_Perm_Res" 3 "Refugee_Protected_Person" 4 "Refugee claimnant" 5 "Temporary_worker" 6 "Int_Student" 7 "Don't_know" 0 "Missing"
label values CITIZEN_STATUS CITIZENPARTICIPANT
codebook CITIZEN_STATUS

label variable STATUS_PARTNER "Partner_citizenship_status"
label define CITIZENPARTNER 1 "Canadian citizen" 2 "landed_imm_Perm_Res" 3 "Refugee_Protected_Person" 4 "Refugee claimnant" 5 "Temporary_worker" 6 "Int_Student" 7 "Don't_know" 0 "Missing"
label values STATUS_PARTNER CITIZENPARTNER
codebook STATUS_PARTNER

*Race1*
label variable RACE_PARTICIPANT "Participant_Race"
```

```
label define RACEPARTICIPANT 1 "Racialized" 2 "Caucasian/White" 3 "Indigenous" 4 "PrefNotSay" 0 "Missing"
label values RACE_PARTICIPANT RACEPARTICIPANT
codebook RACE_PARTICIPANT

label variable RACE_PARTNER "Participant_Race"
label define RACEPARTNER 1 "Racialized" 2 "Caucasian/White" 3 "Indigenous" 4 "PrefNotSay" 0 "Missing"
label values RACE_PARTNER RACEPARTNER
codebook RACE_PARTNER

label variable RACE_PARTICIPANT_2 "Participant Race"
label define RACEPARTICIPANT2 1 "Black" 2 "Caucasian/White" 3 "East Asian" 4 "Indigenous" 5 "Latin American" 6 "Middle East" 7 "North African" 8 "Southeast Asian" 9 "South Asian" 10 "Don't Know" 11 "Prefer Not to Say" 13 "Mixed Race"
label values RACE_PARTICIPANT_2 RACEPARTICIPANT2
codebook RACE_PARTICIPANT_2

label variable RACE_PARTNER_2 "Partner Race OG"
label define RACE_PARTNER2 1 "Black" 2 "Caucasian/White" 3 "East Asian" 4 "Indigenous" 5 "Latin American" 6 "Middle East" 7 "South Asian" 8 "Southeast Asian" 9 "North African" 10 "Don't Know" 11 "Prefer Not to Say" 13 "Mixed Race"
label values RACE_PARTNER_2 RACE_PARTNER2
codebook RACE_PARTNER_2

*Education*

label variable EDUC_PART "Participant_Education"
label define EDUCPART 1 "NorCertificate" 2 "HS_Diploma" 3 "TradeCert/Diploma" 4 "College/CEGEP" 5 "UnivCert/Diploma" 6 "Bachelors" 7 "Masters" 8 "PHD" 0 "Missing"
label values EDUC_PART EDUCPART
codebook EDUC_PART

label variable EDUC_PARTNER "Partner_Education"
label define EDUCPARTNER 1 "NoCertificate" 2 "HS_Diploma" 3 "TradeCert/Diploma" 4 "College/CEGEP" 5 "UnivCert/Diploma" 6 "Bachelors" 7 "Masters" 8 "PHD" 9 "Don't_Know" 0 "Missing"
label values EDUC_PARTNER EDUCPARTNER
codebook EDUC_PARTNER

*Employment Status*

*During COVID*

label variable EMPL_PART_DURING
```

```
"Participant_Employment_Status_during_COVID"
label define EMPPARTICIPANTCOV 1 "Yes" 2 "No" 3 "Retired" 0 "Missing"
label values EMPL_PART_DURING EMPPARTICIPANTCOV
codebook EMPL_PART_DURING

label variable EMP_PARTNER_DURING "Partner_Employment_Status_during_COVID"
label define EMPPARTNERCOV 1 "Yes" 2 "No" 3 "Retired" 4 "Uknown"
label values EMP_PARTNER_DURING EMPPARTNERCOV
codebook EMP_PARTNER_DURING

*Geography*

label variable GEOGRAPHY "Geography"
label define GEO 1 "EASTERNOnt" 2 "CENTRALOnt" 3 "TORONTO" 4 "GTA" 5 "WESTERNOnt" 6 "NORTHERNOnt" 0 "Missing"
label values GEOGRAPHY GEO
codebook GEOGRAPHY

label variable RURALURBAN "Rural or Urban"
label define RU_URB 1 "Rural" 2 "Urban" 0 "Missing"
label values RURALURBAN RU_URB
codebook RURALURBAN

*Household Income*

label variable HH_INCOME "Household_Income"
label define HH 1 "<30,000" 2 "30-39,999" 3 "40-49,999" 4 "50-69,999" 5 "70-99,999" 6 ">100,000" 7 "Don't_Know" 8 "PrefNotSay" 0 "Missing"
label values HH_INCOME HH
codebook HH_INCOME

*Owned Residence During COVID*

label variable OWN_RES_DURING "Own Residence During Pandemic"
label define OWNRES 1 "Yes, I co-own/co-owned my residence with my partner" 2 "Yes, I own/owned the residence_it is under my name only" 3 "No_Hous is/was under partnre's name only" 4 "No, neither own residence we live in" 5 "No_I live/lived with family" 0 "Missing"
label values OWN_RES_DURING OWNRES
codebook OWN_RES_DURING

label variable TYPE_RES_DURING "ResidenceType_During_Pandemic"
label define TYPERES 1 "Single detached" 2 "Apartment_Lowrise" 3 "Apartment_highrise" 4 "TownHome" 5 "Semi-detached" 6"Shelter" 0 "Missing"
label values TYPE_RES_DURING TYPERES
```

```
label values TYPE_RES_DURING TYPERES
codebook TYPE_RES_DURING

**Community Violence**

label variable COMM_VIOL_DURING "Community_Violence_Problem_During COVID"
label define COMVIOL 1 "No" 2 "Yes" 0 "Missing"
label values COMM_VIOL_DURING COMVIOL
codebook COMM_VIOL_DURING

*Partner Type*

label variable PARTNER_TYPE "PARTNER TYPE"
label define PARTTYPE 1 "Wife" 2 "Husband" 3 "ComLawWife" 4 "ComLawHus" 5 "Boyfriend" 6 "Girlfriend" 7 "PrefNotSay" 0 "Missing"
label values PARTNER_TYPE PARTTYPE
codebook PARTNER_TYPE

*length of relationship*

label variable LENGTH_COMBINED "Length of relationship"
label define LENGTH 1 "1-6 months" 2 "7-11 months" 3 "1-2 yrs" 4 ">2yrs" 0 "Missing"
label values LENGTH_COMBINED LENGTH
codebook LENGTH_COMBINED

*PARTNER DRINKING
label variable WISH_DRINK_COMBINED "Wish_did_not_DRINK"
label define DRINK 5 "Almost Always" 4 "Often" 3 "Sometimes" 2 "Seldom" 1 "Never" 0 "Missing"
label values WISH_DRINK_COMBINED DRINK
codebook WISH_DRINK_COMBINED

label variable STRAIN_DRINK_COMBINED "DrinkingSourceOfStrain"
label define STRAINALC 5 "Almost Always" 4 "Often" 3 "Sometimes" 2 "Seldom" 1 "Never" 0 "Missing"
label values STRAIN_DRINK_COMBINED STRAINALC
codebook STRAIN_DRINK_COMBINED

label variable CON_LEAVE_DRINK_COMBINED "ConsideredLeavingBCDRINK"
label define LEAVEALC 5 "Almost Always" 4 "Often" 3 "Sometimes" 2 "Seldom" 1 "Never" 0 "Missing"
label values CON_LEAVE_DRINK_COMBINED LEAVEALC
codebook CON_LEAVE_DRINK_COMBINED

label variable WISH_DRUGS_COMBINED "Wish_did_not_DRUGS"
label define DRUGS 5 "Almost Always" 4 "Often" 3 "Sometimes" 2
```

```
"Seldom" 1 "Never" 0 "Missing"
label values WISH_DRUGS_COMBINED DRUGS
codebook WISH_DRUGS_COMBINED

label variable STRAIN_DRUGS_COMBINED "DRUGSSourceOfStrain"
label define STRAINDRUGS 5 "Almost Always" 4 "Often" 3 "Sometimes" 2
"Seldom" 1 "Never" 0 "Missing"
label values STRAIN_DRUGS_COMBINED STRAINDRUGS
codebook STRAIN_DRUGS_COMBINED

label variable CON_LEAVE_DRUGS_COMBINED "ConsideredLeavingBCDRUGS"
label define LEAVEDRUGS 5 "Almost Always" 4 "Often" 3 "Sometimes" 2
"Seldom" 1 "Never" 0 "Missing"
label values CON_LEAVE_DRUGS_COMBINED LEAVEDRUGS
codebook CON_LEAVE_DRUGS_COMBINED

*EXPERIENCE OF IPV*
*BEFORE COVID*

label variable PHYS_B4 "Physically Hurt You B4 COVID"
label define PHYSB4COV 1 "Never" 2 "Once" 3 "A few times" 4 "monthly"
5 "weekly" 6 "almost dialy" 0 "Missing"
label values PHYS_B4 PHYSB4COV
codebook PHYS_B4

label variable INSULT_B4 "Insult or talk down to you_B4_COVID"
label define INSULTB4COV 1 "Never" 2 "Once" 3 "A few times" 4
"monthly" 5 "weekly" 6 "almost dialy" 0 "Missing"
label values INSULT_B4 INSULTB4COV
codebook INSULT_B4

label variable THREAT_HARM_B4 "Threaten with Harm B4 COVID"
label define HARMB4COVID 1 "Never" 2 "Once" 3 "A few times" 4
"monthly" 5 "weekly" 6 "almost dialy" 0 "Missing"
label values THREAT_HARM_B4 HARMB4COVID
codebook THREAT_HARM_B4

label variable SCREAM_CURSE_B4 "Scream_Curse_at You B4 COVID"
label define SCREAMB4COVID 1 "Never" 2 "Once" 3 "A few times" 4
"monthly" 5 "weekly" 6 "almost dialy" 0 "Missing"
label values SCREAM_CURSE_B4 SCREAMB4COVID
codebook SCREAM_CURSE_B4

label variable SEXUAL_B4 "Force You to have sexual activities B4
COVID"
label define SEXUALB4COVID 1 "Never" 2 "Once" 3 "A few times" 4
"monthly" 5 "weekly" 6 "almost dialy" 0 "Missing"
label values SEXUAL_B4 SEXUALB4COVID
```

```
codebook SEXUAL_B4

label variable EMP_MON_B4 "Keep you from access to job/employ_B4_COVID"
label define KEEPEMPMONB4COVID 1 "Never" 2 "Once" 3 "A few times" 4 "monthly" 5 "weekly" 6 "almost dialy" 0 "Missing"
label values EMP_MON_B4 KEEPEMPMONB4COVID
codebook EMP_MON_B4

label variable FAM_FRIEND_B4 "Keep_from seeing_FAM_FRNDS_B4_COVID"
label define KEEPSEEFAMFRNDB4COVID 1 "Never" 2 "Once" 3 "A few times" 4 "monthly" 5 "weekly" 6 "almost dialy" 0 "Missing"
label values FAM_FRIEND_B4 KEEPSEEFAMFRNDB4COVID
codebook FAM_FRIEND_B4

label variable TECH_DIGIT_B4 "Tech/Digital_Violence_B4_COVID"
label define TECHVIOLB4COVID 1 "Never" 2 "Once" 3 "A few times" 4 "monthly" 5 "weekly" 6 "almost dialy" 0 "Missing"
label values TECH_DIGIT_B4 TECHVIOLB4COVID
codebook TECH_DIGIT_B4

label variable REL_SPIRT_B4 "Religious_Spirit_VIOL_B4_COVID"
label define RELIGVIOLB4COVID 1 "Never" 2 "Once" 3 "A few times" 4 "monthly" 5 "weekly" 6 "almost dialy" 0 "Missing"
label values REL_SPIRT_B4 RELIGVIOLB4COVID
codebook REL_SPIRT_B4

*DURING COVID*

label variable PHYS_DURING "Physically Hurt You DURING COVID"
label define PHYSinCOV 1 "Did not happen" 2 "MuchBetter" 3 "SomewhatBetter" 4 "StayedtheSAME" 5 "SOmwhatWorse" 6 "MuchWorse"
label values PHYS_DURING PHYSinCOV
codebook PHYS_DURING

label variable INSULT_DURING "Insult or talk down to you_DURING_COVID"
label define INSULTinCOV 1 "Did not happen" 2 "MuchBetter" 3 "SomewhatBetter" 4 "StayedtheSAME" 5 "SOmwhatWorse" 6 "MuchWorse"
label values INSULT_DURING INSULTinCOV
codebook INSULT_DURING

label variable THREAT_HARM_DURING "Threaten with Harm DURING COVID"
label define HARMinCOVID 1 "Did not happen" 2 "MuchBetter" 3 "SomewhatBetter" 4 "StayedtheSAME" 5 "SOmwhatWorse" 6 "MuchWorse"
label values THREAT_HARM_DURING HARMinCOVID
codebook THREAT_HARM_DURING

label variable SCREAM_CURSE_DURING "Scream_Curse_at You DURING COVID"
```

```
label variable SCREAM_CURSE_DURING "Scream_Curse_at You DURING COVID"
label define SCREAMinCOVID 1 "Did not happen" 2 "MuchBetter" 3 "SomewhatBetter" 4 "StayedtheSAME" 5 "SOmwhatWorse" 6 "MuchWorse"
label values SCREAM_CURSE_DURING SCREAMinCOVID
codebook SCREAM_CURSE_DURING

label variable SEXUAL_DURING "Force You to have sexual activities DURING COVID"
label define SEXUALinCOVID 1 "Did not happen" 2 "MuchBetter" 3 "SomewhatBetter" 4 "StayedtheSAME" 5 "SOmwhatWorse" 6 "MuchWorse"
label values SEXUAL_DURING SEXUALinCOVID
codebook SEXUAL_DURING

label variable EMP_MON_DURING "Keep you from access to job/employ_DURING_COVID"
label define KEEPEMPMONinCOVID 1 "Did not happen" 2 "MuchBetter" 3 "SomewhatBetter" 4 "StayedtheSAME" 5 "SOmwhatWorse" 6 "MuchWorse"
label values EMP_MON_DURING KEEPEMPMONinCOVID
codebook EMP_MON_DURING

label variable FAM_FRIEND_DURING "Keep_from seeing_FAM_FRNDS_DURING_COVID"
label define KEEPSEEFAMFRNDinCOVID 1 "Did not happen" 2 "MuchBetter" 3  "SomewhatBetter" 4 "StayedtheSAME" 5 "SOmwhatWorse" 6 "MuchWorse"
label values FAM_FRIEND_DURING KEEPSEEFAMFRNDinCOVID
codebook FAM_FRIEND_DURING

label variable TECH_DIGIT_DURING "Tech/Digital_Violence_DURING_COVID"
label define TECHVIOLinCOVID 1 "Did not happen" 2 "MuchBetter" 3 "SomewhatBetter" 4 "StayedtheSAME" 5 "SOmwhatWorse" 6 "MuchWorse"
label values TECH_DIGIT_DURING TECHVIOLinCOVID
codebook TECH_DIGIT_DURING

label variable RELI_SPRIT_DURING "Religious_Spirit_VIOL_DURING_COVID"
label define RELIGVIOLinCOVID 1 "Did not happen" 2 "MuchBetter" 3 "SomewhatBetter" 4 "StayedtheSAME" 5 "SOmwhatWorse" 6 "MuchWorse"
label values RELI_SPRIT_DURING RELIGVIOLinCOVID
codebook RELI_SPRIT_DURING

*POST_1 COVID*

label variable PHYS_POST_1 "Physically Hurt You POST COVID"
label define PHYSPOSTCOV 1 "Did not happen" 2 "MuchBetter" 3 "SomewhatBetter" 4 "StayedtheSAME" 5 "SOmwhatWorse" 6 "MuchWorse"
label values PHYS_POST_1 PHYSPOSTCOV
codebook PHYS_POST_1
```

```
codebook PHYS_POST_1

label variable INSULT_POST_1 "Insult or talk down to you_POST_COVID"
label define INSULTPOSTCOV 1 "Did not happen" 2 "MuchBetter" 3 "SomewhatBetter" 4 "StayedtheSAME" 5 "SOmwhatWorse" 6 "MuchWorse"
label values INSULT_POST_1 INSULTPOSTCOV
codebook INSULT_POST_1

label variable THREAT_HARM_POST_1 "Threaten with Harm POST COVID"
label define THREAT_HARM_POST_BOTH 1 "Did not happen" 2 "MuchBetter" 3 "SomewhatBetter" 4 "StayedtheSAME" 5 "SOmwhatWorse" 6 "MuchWorse"
label values THREAT_HARM_POST_1 HARMPOSTCOVID
codebook THREAT_HARM_POST_1

label variable SCREAM_CURSE_POST_1 "Scream_Curse_at You POST COVID"
label define SCREAMPOSTCOVID 1 "Did not happen" 2 "MuchBetter" 3 "SomewhatBetter" 4 "StayedtheSAME" 5 "SOmwhatWorse" 6 "MuchWorse"
label values SCREAM_CURSE_POST_1 SCREAMPOSTCOVID
codebook SCREAM_CURSE_POST_1

label variable SEXUAL_POST_1 "Force You to have sexual activities POST COVID"
label define SEXUALPOSTCOVID 1 "Did not happen" 2 "MuchBetter" 3 "SomewhatBetter" 4 "StayedtheSAME" 5 "SOmwhatWorse" 6 "MuchWorse"
label values SEXUAL_POST_1 SEXUALPOSTCOVID
codebook SEXUAL_POST_1

label variable EMP_MON_POST_1 "Keep you frmo access to job/employ_POST_COVID"
label define KEEPEMPMONPOSTCOVID 1 "Did not happen" 2 "MuchBetter" 3 "SomewhatBetter" 4 "StayedtheSAME" 5 "SOmwhatWorse" 6 "MuchWorse"
label values EMP_MON_POST_1 KEEPEMPMONPOSTCOVID
codebook EMP_MON_POST_1

label variable FAM_FRIEND_POST_1 "Keep_from seeing_FAM_FRNDS_POST_COVID"
label define KEEPSEEFAMFRNDPOSTCOVID 1 "Did not happen" 2 "MuchBetter" 3 "SomewhatBetter" 4 "StayedtheSAME" 5 "SOmwhatWorse" 6 "MuchWorse"
label values FAM_FRIEND_POST_1 KEEPSEEFAMFRNDPOSTCOVID
codebook FAM_FRIEND_POST_1

label variable TECH_DIGIT_POST_1 "Tech/Digital_Violence_POST_COVID"
label define TECHVIOLPOSTCOVID 1 "Did not happen" 2 "MuchBetter" 3 "SomewhatBetter" 4 "StayedtheSAME" 5 "SOmwhatWorse" 6 "MuchWorse"
label values TECH_DIGIT_POST_1 TECHVIOLPOSTCOVID
codebook TECH_DIGIT_POST_1

label variable REL_SPIRT_POST_1 "Religious_Spirit_VIOL_POST_COVID"
```

```
label variable REL_SPIRT_POST_1 "Religious_Spirit_VIOL_POST_COVID"
label define RELIGVIOLPOSTCOVID 1 "Did not happen" 2 "MuchBetter" 3 "SomewhatBetter" 4 "StayedtheSAME" 5 "SOmwhatWorse" 6 "MuchWorse"
label values REL_SPIRT_POST_1 RELIGVIOPOSTCOVID
codebook REL_SPIRT_POST_1


*POST_2 COVID*

label variable PHYS_POST_2 "Physically Hurt You POST COVID"
label define PHYSPOSTCOV2 1 "Did not happen" 2 "MuchBetter" 3 "SomewhatBetter" 4 "StayedtheSAME" 5 "SOmwhatWorse" 6 "MuchWorse"
label values PHYS_POST_2 PHYSPOSTCOV
codebook PHYS_POST_2

label variable INSULT_POST_2 "Insult or talk down to you_POST_COVID"
label define INSULTPOSTCOV2 1 "Did not happen" 2 "MuchBetter" 3 "SomewhatBetter" 4 "StayedtheSAME" 5 "SOmwhatWorse" 6 "MuchWorse"
label values INSULT_POST_2 INSULTPOSTCOV
codebook INSULT_POST_2

label variable THREAT_HARM_POST_2 "Threaten with Harm POST COVID"
label define THREAT_HARM_POSTCOV2 1 "Did not happen" 2 "MuchBetter" 3 "SomewhatBetter" 4 "StayedtheSAME" 5 "SOmwhatWorse" 6 "MuchWorse"
label values THREAT_HARM_POST_2 HARMPOSTCOVID
codebook THREAT_HARM_POST_2

label variable SCREAM_CURSE_POST_2 "Scream_Curse_at You POST COVID"
label define SCREAMPOSTCOVID2 1 "Did not happen" 2 "MuchBetter" 3 "SomewhatBetter" 4 "StayedtheSAME" 5 "SOmwhatWorse" 6 "MuchWorse"
label values SCREAM_CURSE_POST_2 SCREAMPOSTCOVID
codebook SCREAM_CURSE_POST_2

label variable SEXUAL_POST_2 "Force You to have sexual activities POST COVID"
label define SEXUALPOSTCOVID2 1 "Did not happen" 2 "MuchBetter" 3 "SomewhatBetter" 4 "StayedtheSAME" 5 "SOmwhatWorse" 6 "MuchWorse"
label values SEXUAL_POST_2 SEXUALPOSTCOVID
codebook SEXUAL_POST_2

label variable EMP_MON_POST_2 "Keep you frmo access to job/employ_POST_COVID"
label define KEEPEMPMONPOSTCOVID2 1 "Did not happen" 2 "MuchBetter" 3 "SomewhatBetter" 4 "StayedtheSAME" 5 "SOmwhatWorse" 6 "MuchWorse"
label values EMP_MON_POST_2 KEEPEMPMONPOSTCOVID
codebook EMP_MON_POST_2

label variable FAM_FRIEND_POST_2 "Keep_from
```

```
seeing_FAM_FRNDS_POST_COVID"
label define KEEPSEEFAMFRNDPOSTCOVID2 1 "Did not happen" 2 "MuchBetter" 3 "SomewhatBetter" 4 "StayedtheSAME" 5 "SOmwhatWorse" 6 "MuchWorse"
label values FAM_FRIEND_POST_2 KEEPSEEFAMFRNDPOSTCOVID
codebook FAM_FRIEND_POST_2

label variable TECH_DIGIT_POST_2 "Tech/Digital_Violence_POST_COVID"
label define TECHVIOLPOSTCOVID2 1 "Did not happen" 2 "MuchBetter" 3 "SomewhatBetter" 4 "StayedtheSAME" 5 "SOmwhatWorse" 6 "MuchWorse"
label values TECH_DIGIT_POST_2 TECHVIOLPOSTCOVID
codebook TECH_DIGIT_POST_2

label variable REL_SPIRT_POST_2 "Religious_Spirit_VIOL_POST_COVID"
label define RELIGVIOLPOSTCOVID2 1 "Did not happen" 2 "MuchBetter" 3 "SomewhatBetter" 4 "StayedtheSAME" 5 "SOmwhatWorse" 6 "MuchWorse"
label values REL_SPIRT_POST_2 RELIGVIOPOSTCOVID
codebook REL_SPIRT_POST_2

**********************
*RECODING***
*********************

***DROPPING CASES****

drop if DROP==1
tab DROP, m


*********************************
*   RECODING VARIABLES  *
*********************************

********************************************************************************
*INDEPENDANT_EXPLANATORY VARIABLES: Q1. EXPERIENCE OF IPV DURING COVID
********************************************************************************

recode IPV_EXP (1=1 "Yes IPV") (2=0 "No IPV") (.=.), gen (dumYES_IPV)
tab IPV_EXP dumYES_IPV, m

recode IPV_EXP (2=1 "No IPV") (1=0 "Yes IPV") (.=.), gen (dumNo_IPV)
tab IPV_EXP dumNo_IPV, m

**************************************************************************
```

```
**************************************************************************

*OUTCOME VARIABLE #1 :Accessibility to Services ****Barriers to Supports during COVID19

*************************************************************************

*Community Based Organization*
recode BARR_COMMBASE_DURING (1=1 "yes") (2 3=0 "other") (0=.) (.=.), gen (dumBarrCOMMBASW)
tab BARR_COMMBASE_DURING dumBarrCOMMBASW, m

recode BARR_COMMBASE_DURING (2=1 "No") (1 3=0 "other") (0=.) (.=.), gen (dumNO_BarrCOMMBASW)
tab BARR_COMMBASE_DURING dumNO_BarrCOMMBASW, m

recode BARR_COMMBASE_DURING (3=1 "Did Not Try") (1 2=0 "other") (0=.) (.=.), gen (dumNO_TRY_COMMBASW)
tab BARR_COMMBASE_DURING dumNO_TRY_COMMBASW, m

*Counseling*
recode BARR_COUNS_DURING (1=1 "yes") (2 3=0 "other") (0=.) (.=.), gen (dumBarrCOUNSELING)
tab BARR_COUNS_DURING dumBarrCOUNSELING, m

recode BARR_COUNS_DURING (2=1 "No") (1 3=0 "other") (0=.) (.=.), gen ( dumNO_BarrCOUNSELING)
tab BARR_COUNS_DURING dumNO_BarrCOUNSELING, m

recode BARR_COUNS_DURING (3=1 "Did Not Try") (1 2=0 "other") (0=.) (.=.), gen (dumNO_TRY_COUNSELING)
tab BARR_COUNS_DURING dumNO_TRY_COUNSELING, m

*HealthCare*
recode BARR_HEALTHCR_DURING (1=1 "yes") (2 3=0 "other") (0=.) (.=.), gen (dumBarrHEALTHCARE)
tab BARR_HEALTHCR_DURING dumBarrHEALTHCARE, m

recode BARR_HEALTHCR_DURING (2=1 "No") (1 3=0 "other") (0=.) (.=.), gen (dumNO_BarrHEALTHCARE)
tab BARR_HEALTHCR_DURING dumNO_BarrHEALTHCARE, m

recode BARR_HEALTHCR_DURING (3=1 "Did Not Try") (1 2=0 "other") (0=.) (.=.), gen (dumNO_TRY_HEALTHCARE)
tab BARR_HEALTHCR_DURING dumNO_TRY_HEALTHCARE, m
```

```
recode BARR_SETT_DURING (1=1 "yes") (2 3=0 "other") (0=.) (.=.), gen (
dumBarrSETTLEMENTAG)
tab BARR_SETT_DURING dumBarrSETTLEMENTAG, m

recode BARR_SETT_DURING (2=1 "No") (1 3=0 "other") (0=.) (.=.), gen (
dumNO_BarrSETTLEMENTAG)
tab BARR_SETT_DURING dumNO_BarrSETTLEMENTAG, m

recode BARR_SETT_DURING (3=1 "Did Not Try") (1 2=0 "other") (0=.)
(.=.), gen (dumNO_TRY_SETTLEMENTAG)
tab BARR_SETT_DURING dumNO_TRY_SETTLEMENTAG, m

*Shelter*
recode BARR_EMER_SHELT_DURING (1=1 "yes") (2 3=0 "other") (0=.) (.=.),
 gen (dumBarrEMERSHELTER)
tab BARR_EMER_SHELT_DURING dumBarrEMERSHELTER, m

recode BARR_EMER_SHELT_DURING (2=1 "No") (1 3=0 "other") (0=.) (.=.),
gen (dumNO_BarrEMERSHELTER)
tab BARR_EMER_SHELT_DURING dumNO_BarrEMERSHELTER, m

recode BARR_EMER_SHELT_DURING (3=1 "Did Not Try") (1 2=0 "other") (0
=.) (.=.), gen (dumNO_TRY_EMERSHELTER)
tab BARR_EMER_SHELT_DURING dumNO_TRY_EMERSHELTER, m

*Crisis Line*
recode BARR_CRISISLINE_DURING (1=1 "yes") (2 3=0 "other") (0=.) (.=.),
 gen (dumBarrCRISISLINE)
tab BARR_CRISISLINE_DURING dumBarrCRISISLINE, m

recode BARR_CRISISLINE_DURING (2=1 "No") (1 3=0 "other") (0=.) (.=.),
gen (dumNO_BarrCRISISLINE)
tab BARR_CRISISLINE_DURING dumNO_BarrCRISISLINE, m

recode BARR_CRISISLINE_DURING (3=1 "Did Not Try") (1 2=0 "other") (0
=.) (.=.), gen (dumNO_TRY_CRISISLINE)
tab BARR_CRISISLINE_DURING dumNO_TRY_CRISISLINE, m

*Child Protection
recode BARR_CHILDPROT_DURING (1=1 "yes") (2 3=0 "other") (0=.) (.=.),
gen (dumBarrCHILDPROTECT)
tab BARR_CHILDPROT_DURING dumBarrCHILDPROTECT, m

recode BARR_CHILDPROT_DURING (2=1 "No") (1 3=0 "other") (0=.) (.=.),
gen (dumNO_BarrCHILDPROTECT)
tab BARR_CHILDPROT_DURING dumNO_BarrCHILDPROTECT, m

recode BARR_CHILDPROT_DURING (3=1 "Did Not Try") (1 2=0 "other") (0=.)
```

```
recode BARR_CHILDPROT_DURING (3=1 "Did Not Try") (1 2=0 "other") (0=.) (.=.), gen (dumNO_TRY_CHILDPROTECT)
tab BARR_CHILDPROT_DURING dumNO_TRY_CHILDPROTECT, m

*Legal Counsel*
recode BARR_LEGAL_DURING (1=1 "yes") (2 3=0 "other") (0=.) (.=.), gen (dumBarrLEGAL)
tab BARR_LEGAL_DURING dumBarrLEGAL, m

recode BARR_LEGAL_DURING (2=1 "No") (1 3=0 "other") (0=.) (.=.), gen (dumNO_BarrLEGAL)
tab BARR_LEGAL_DURING dumNO_BarrLEGAL, m

recode BARR_LEGAL_DURING (3=1 "Did Not Try") (1 2=0 "other") (0=.) (.=.), gen (dumNO_TRY_LEGAL)
tab BARR_LEGAL_DURING dumNO_TRY_LEGAL, m

*Mental Health*
recode BARR_MENT_HC_DURING (1=1 "yes") (2 3=0 "other") (0=.) (.=.), gen (dumBarrMENTALH)
tab BARR_MENT_HC_DURING dumBarrMENTALH, m

recode BARR_MENT_HC_DURING (2=1 "No") (1 3=0 "other") (0=.) (.=.), gen (dumNO_BarrMENTALH)
tab BARR_MENT_HC_DURING dumNO_BarrMENTALH, m

recode BARR_MENT_HC_DURING (3=1 "Did Not Try") (1 2=0 "other") (0=.) (.=.), gen (dumNO_TRY_MENTALH)
tab BARR_MENT_HC_DURING dumNO_TRY_MENTALH, m

*Addiction Services*
recode BARR_ADDICT_DURING (1=1 "yes") (2 3=0 "other") (0=.) (.=.), gen (dumBarrADDICT)
tab BARR_ADDICT_DURING dumBarrADDICT, m

recode BARR_ADDICT_DURING (2=1 "No") (1 3=0 "other") (0=.) (.=.), gen (dumNO_BarrADDICT)
tab BARR_ADDICT_DURING dumNO_BarrADDICT, m

recode BARR_ADDICT_DURING (3=1 "Did Not Try") (1 2=0 "other") (0=.) (.=.), gen (dumNO_TRY_ADDICT)
tab BARR_ADDICT_DURING dumNO_TRY_ADDICT, m

*Housing*
recode BARR_HOUSING_DURING (1=1 "yes") (2 3=0 "other") (0=.) (.=.), gen (dumBarrHOUSING)
tab BARR_HOUSING_DURING dumBarrHOUSING, m
```

```
recode BARR_HOUSING_DURING (2=1 "No") (1 3=0 "other") (0=.) (.=.), gen (dumNO_BarrHOUSING)
tab BARR_HOUSING_DURING dumNO_BarrHOUSING, m

recode BARR_HOUSING_DURING (3=1 "Did Not Try") (1 2=0 "other") (0=.) (.=.), gen (dumNO_TRY_HOUSING)
tab BARR_HOUSING_DURING dumNO_TRY_HOUSING, m

*Transportation*

recode BARR_TRANS_DURING (1=1 "yes") (2 3=0 "other") (0=.) (.=.), gen (dumBarrTRANSPORT)
tab BARR_TRANS_DURING dumBarrTRANSPORT, m

recode BARR_TRANS_DURING (2=1 "No") (1 3=0 "other") (0=.) (.=.), gen ( dumNO_BarrTRANSPORT)
tab BARR_TRANS_DURING dumNO_BarrTRANSPORT, m

recode BARR_TRANS_DURING (3=1 "Did Not Try") (1 2=0 "other") (0=.) (.=.), gen (dumNO_TRY_TRANSPORT)
tab BARR_TRANS_DURING dumNO_TRY_TRANSPORT, m

**NOTES JUSTIFICATION FOR THRESHOLD: In determining the threshold for 'yes barriers,' we utilized the distribution of barriers faced by respondents as indicated by the centile analysis. The analysis revealed that the 75th percentile of respondents experienced up to 2 barriers, while those above this threshold, at the 90th percentile, faced 4 or more barriers. To ensure that our indicator captures significant access challenges, we defined 'yes barriers' as facing more than 2 barriers. This threshold is appropriate because it distinguishes respondents who encounter moderate barriers from those who experience a higher level of difficulty, reflecting a more substantial impediment to accessing essential services*

gen sumdumBarriers = dumBarrTRANSPORT + dumBarrHOUSING + dumBarrADDICT + dumBarrMENTALH +dumBarrLEGAL + dumBarrCHILDPROTECT + dumBarrSETTLEMENTAG + dumBarrEMERSHELTER + dumBarrHEALTHCARE + dumBarrCRISISLINE + dumBarrCOUNSELING + dumBarrCOMMBASW
tab sumdumBarriers, m

mean sumdumBarriers
sum sumdumBarriers, d

centile sumdumBarriers, centile(50 75 80 85 90)

*Facing barriers to ACCESSING FORMAL SUPPORT SERVICES

*75th percentile*
```

```
recode sumdumBarriers (0 1 2=0 "No Barriers") (3/12=1 "Yes Barriers") (.=.), gen (dumBarriersAccess75)
tab sumdumBarriers dumBarriersAccess75, m

recode sumdumBarriers (0 1 2=1 "No Barriers") (3/12=0 "Yes Barriers") (.=.), gen (dumNOBarriersAccess75)
tab sumdumBarriers dumNOBarriersAccess75, m

*85th percentile*

recode sumdumBarriers (0 1 2 3 =0 "No Barriers") (4/12=1 "Yes Barriers") (.=.), gen (dumBarriersAccess85)
tab sumdumBarriers dumBarriersAccess85, m

recode sumdumBarriers (0 1 2 3=1 "No Barriers") (4/12=0 "Yes Barriers") (.=.), gen (dumNOBarriersAccess85)
tab sumdumBarriers dumNOBarriersAccess85, m

*90th percentile*

recode sumdumBarriers (0 1 2 3 4=0 "No Barriers") (5/12=1 "Yes Barriers") (.=.), gen (dumBarriersAccess90)
tab sumdumBarriers dumBarriersAccess85, m

recode sumdumBarriers (0 1 2 3 4=1 "No Barriers") (5/12=0 "Yes Barriers") (.=.), gen (dumNOBarriersAccess90)
tab sumdumBarriers dumNOBarriersAccess85, m


***Tried to access vs Did not try***

********************************************************
*   RECODING INTO TRIED/DID NOT TRY TO ACCESS SERVICES  *
********************************************************

* Community Based Organization
recode BARR_COMMBASE_DURING (1 2=1 "Tried") (3=0 "Did Not Try") (0=.) (.=.), gen(dumTried_COMMBASW)
tab BARR_COMMBASE_DURING dumTried_COMMBASW, m

* Counseling
recode BARR_COUNS_DURING (1 2=1 "Tried") (3=0 "Did Not Try") (0=.) (.=.), gen(dumTried_COUNSELING)
tab BARR_COUNS_DURING dumTried_COUNSELING, m

* HealthCare
recode BARR_HEALTHCR_DURING (1 2=1 "Tried") (3=0 "Did Not Try") (0=.)
```

```
recode BARR_HEALTHCR_DURING (1 2=1 "Tried") (3=0 "Did Not Try") (0=.)
(.=.), gen(dumTried_HEALTHCARE)
tab BARR_HEALTHCR_DURING dumTried_HEALTHCARE, m

* Settlement
recode BARR_SETT_DURING (1 2=1 "Tried") (3=0 "Did Not Try") (0=.)
(.=.), gen(dumTried_SETTLEMENT)
tab BARR_SETT_DURING dumTried_SETTLEMENT, m

* Shelter
recode BARR_EMER_SHELT_DURING (1 2=1 "Tried") (3=0 "Did Not Try") (0
=.) (.=.), gen(dumTried_EMERSHELTER)
tab BARR_EMER_SHELT_DURING dumTried_EMERSHELTER, m

* Crisis Line
recode BARR_CRISISLINE_DURING (1 2=1 "Tried") (3=0 "Did Not Try") (0
=.) (.=.), gen(dumTried_CRISISLINE)
tab BARR_CRISISLINE_DURING dumTried_CRISISLINE, m

* Child Protection
recode BARR_CHILDPROT_DURING (1 2=1 "Tried") (3=0 "Did Not Try") (0=.)
 (.=.), gen(dumTried_CHILDPROTECT)
tab BARR_CHILDPROT_DURING dumTried_CHILDPROTECT, m

* Legal Counsel
recode BARR_LEGAL_DURING (1 2=1 "Tried") (3=0 "Did Not Try") (0=.)
(.=.), gen(dumTried_LEGAL)
tab BARR_LEGAL_DURING dumTried_LEGAL, m

* Mental Health
recode BARR_MENT_HC_DURING (1 2=1 "Tried") (3=0 "Did Not Try") (0=.)
(.=.), gen(dumTried_MENTALH)
tab BARR_MENT_HC_DURING dumTried_MENTALH, m

* Addiction Services
recode BARR_ADDICT_DURING (1 2=1 "Tried") (3=0 "Did Not Try") (0=.)
(.=.), gen(dumTried_ADDICT)
tab BARR_ADDICT_DURING dumTried_ADDICT, m

* Housing
recode BARR_HOUSING_DURING (1 2=1 "Tried") (3=0 "Did Not Try") (0=.)
(.=.), gen(dumTried_HOUSING)
tab BARR_HOUSING_DURING dumTried_HOUSING, m

* Transportation
recode BARR_TRANS_DURING (1 2=1 "Tried") (3=0 "Did Not Try") (0=.)
(.=.), gen(dumTried_TRANSPORT)
tab BARR_TRANS_DURING dumTried_TRANSPORT, m
```

```
************************************************
*Psychological and Physiological health pathways
************************************************

**CREATING A BINARY VARIABLE**

*MENTAL HEALTH*

* Step 1: Generate a new binary variable for mental health outcomes
gen MENTAL_HEALTH_OUTCOME = .

* Step 2: Assign "Bad Mental Health" (1) to those who:
* - Said "About the Same" during COVID-19 but were "Poor" before COVID-19
* - Said "Worse" or "Much Worse" during COVID-19
replace MENTAL_HEALTH_OUTCOME = 1 if MENTAL_IN_COVID == 3 & MENTAL_B4_COVID == 5
replace MENTAL_HEALTH_OUTCOME = 1 if inlist(MENTAL_IN_COVID, 4, 5)

* Step 3: Assign "Good Mental Health" (0) to those who:
* - Said "About the Same" during COVID-19 but had better mental health (Excellent, Very Good, Good, or Fair) before COVID-19
* - Said "Better" or "Much Better" during COVID-19
replace MENTAL_HEALTH_OUTCOME = 0 if MENTAL_IN_COVID == 3 & inlist(MENTAL_B4_COVID, 1, 2, 3, 4)
replace MENTAL_HEALTH_OUTCOME = 0 if inlist(MENTAL_IN_COVID, 1, 2)

* Step 4: Assign "Missing" to other cases (e.g., missing values in the original variables)
replace MENTAL_HEALTH_OUTCOME = . if MENTAL_IN_COVID == 0 | MENTAL_B4_COVID == 0

* Step 5: Label the new variable and values for clarity
label variable MENTAL_HEALTH_OUTCOME "Mental Health Outcome During COVID-19"
label define MENTOUTCOME 0 "Good Mental Health" 1 "Bad Mental Health"
label values MENTAL_HEALTH_OUTCOME MENTOUTCOME

*PHYSICAL HEALTH*
* Step 1: Generate a new binary variable for physical health outcomes
gen PHYSICAL_HEALTH_OUTCOME = .

* Step 2: Assign "Bad Physical Health" (1) to those who:
* - Said "About the Same" during COVID-19 but were "Poor" before COVID-19
* - Said "Worse" or "Much Worse" during COVID-19
replace PHYSICAL_HEALTH_OUTCOME = 1 if PHYS_IN_COVID == 3 & PHYS_B4_COVID == 5
```

```
replace PHYSICAL_HEALTH_OUTCOME = 1 if PHYS_IN_COVID == 3 & PHYS_B4_COVID == 5
replace PHYSICAL_HEALTH_OUTCOME = 1 if inlist(PHYS_IN_COVID, 4, 5)

* Step 3: Assign "Good Physical Health" (0) to those who:
* - Said "About the Same" during COVID-19 but had better physical health (Excellent, Very Good, Good, or Fair) before COVID-19
* - Said "Better" or "Much Better" during COVID-19
replace PHYSICAL_HEALTH_OUTCOME = 0 if PHYS_IN_COVID == 3 & inlist(PHYS_B4_COVID, 1, 2, 3, 4)
replace PHYSICAL_HEALTH_OUTCOME = 0 if inlist(PHYS_IN_COVID, 1, 2)

* Step 4: Assign "Missing" to other cases (e.g., missing values in the original variables)
replace PHYSICAL_HEALTH_OUTCOME = . if PHYS_IN_COVID == 0 | PHYS_B4_COVID == 0

* Step 5: Label the new variable and values for clarity
label variable PHYSICAL_HEALTH_OUTCOME "Physical Health Outcome During COVID-19"
label define PHYSOUTCOME 0 "Good Physical Health" 1 "Bad Physical Health"
label values PHYSICAL_HEALTH_OUTCOME PHYSOUTCOME

recode MENTAL_HEALTH_OUTCOME (1 = 1 "BadMental") (0=0 "GoodMental"), gen (BadMentHealth)
tab MENTAL_HEALTH_OUTCOME BadMentHealth, m

recode MENTAL_HEALTH_OUTCOME (1 = 0 "BadMental") (0=1 "GoodMental"), gen (GoodMentHealth)
tab MENTAL_HEALTH_OUTCOME GoodMentHealth, m

recode PHYSICAL_HEALTH_OUTCOME (1 = 1 "BadPhys") (0=0 "GoodPhys"), gen (BadPhysHealth)
tab PHYSICAL_HEALTH_OUTCOME BadPhysHealth, m

recode PHYSICAL_HEALTH_OUTCOME (1 = 0 "BadPhys") (0=1 "GoodPhys"), gen (GoodPhysHealth)
tab PHYSICAL_HEALTH_OUTCOME GoodPhysHealth, m

************************************************************
*BEHAVIOURS* COVARIATES
************************************************************


**Change in seeking informal supports during COVID19*

recode INFORMAL_DURING (1=1 "increased") (2 3 =0 "Other") (0=.) (.=.),
```

```
recode INFORMAL_DURING (1=1 "increased") (2 3 =0 "Other") (0=.) (.=.), gen (SeekingINFIncreased)
tab INFORMAL_DURING SeekingINFIncreased, m

recode INFORMAL_DURING (2=1 "decreased") (1 3 =0 "Other") (0=.) (.=.), gen (SeekingINFdecreased)
tab INFORMAL_DURING SeekingINFdecreased, m

recode INFORMAL_DURING (3 =1 "No Change") (1 2 =0 "Other") (0=.) (.=.), gen (NoChangeInSeekingINF)
tab INFORMAL_DURING NoChangeInSeekingINF, m

***Informal Seeking2***
recode INFORMAL_DURING (1 3=1 "increased/nochange") (2=0 "decreased") (0=.) (.=.), gen (SeekingINFIncreased_NC2)
tab INFORMAL_DURING SeekingINFIncreased_NC2, m

recode INFORMAL_DURING (2=1 "decreased") (1 3 =0 "increased/nochange") (0=.) (.=.), gen (SeekingINFdecreased2)
tab INFORMAL_DURING SeekingINFdecreased2, m

**Change in seeking formal supports during COVID19*

recode FORMAL_DURING (1=1 "increased") (2 3 =0 "Other") (0=.) (.=.), gen (SeekingFORMIncreased)
tab FORMAL_DURING SeekingFORMIncreased, m

recode FORMAL_DURING (2=1 "decreased") (1 3 =0 "Other") (0=.) (.=.), gen (SeekingFORMdecreased)
tab FORMAL_DURING SeekingFORMdecreased, m

recode FORMAL_DURING (3 =1 "No Change") (1 2 =0 "Other") (0=.) (.=.), gen (NoChangeInSeekingFORM)
tab FORMAL_DURING NoChangeInSeekingFORM, m

**Formal seeking 2**

recode FORMAL_DURING (1 3=1 "increased/nochange") (2=0 "decreased") (0=.) (.=.), gen (SeekingFORMIncreased_NC2)
tab FORMAL_DURING SeekingFORMIncreased_NC2, m

recode FORMAL_DURING (2=1 "decreased") (1 3 =0 "increased/nochange") (0=.) (.=.), gen (SeekingFORMdecreased2)
tab FORMAL_DURING SeekingFORMdecreased2, m

****Change in communicating with Friends during COVID19*

recode COMM_FRND_DURING (1=1 "increased") (2 3 =0 "Other") (0=.)
```

```
tab COMM_FRND_DURING CommFRNDSIncreased, m

recode COMM_FRND_DURING (2=1 "decreased") (1 3 =0 "Other") (0=.) (.=.), gen (CommFRNDSdecreased)
tab COMM_FRND_DURING CommFRNDSdecreased, m

recode COMM_FRND_DURING (3 =1 "No Change") (1 2 =0 "Other") (0=.) (.=.), gen (NoChangeCommFRNDS)
tab COMM_FRND_DURING NoChangeCommFRNDS, m

***Communicating with friends 2*

recode COMM_FRND_DURING (1 3=1 "increased/nochange") (2=0 "decreased") (0=.) (.=.), gen (CommFRNDSIncreased_NC2)
tab COMM_FRND_DURING CommFRNDSIncreased_NC2, m

recode COMM_FRND_DURING (2=1 "decreased") (1 3 =0 "increased/nochange") (0=.) (.=.), gen (CommFRNDSdecreased2)
tab COMM_FRND_DURING CommFRNDSdecreased2, m

****Change in communicating with family during COVID19*

recode COMM_FAM_DURING (1=1 "increased") (2 3 =0 "Other") (0=.) (.=.), gen (CommFAMILYSIncreased)
tab COMM_FAM_DURING CommFAMILYSIncreased, m

recode COMM_FAM_DURING (2=1 "decreased") (1 3 =0 "Other") (0=.) (.=.), gen (CommFAMILYdecreased)
tab COMM_FAM_DURING CommFAMILYdecreased, m

recode COMM_FAM_DURING (3 =1 "No Change") (1 2 =0 "Other") (0=.) (.=.), gen (NoChangeFAMILYCommFRNDS)
tab COMM_FAM_DURING NoChangeFAMILYCommFRNDS, m

***Communicating with family 2*

recode COMM_FAM_DURING (1 3=1 "increased/nochange") (2=0 "decreased") (0=.) (.=.), gen (CommFAMILYSIncreased_NC2)
tab COMM_FAM_DURING CommFAMILYSIncreased_NC2, m

recode COMM_FAM_DURING (2=1 "decreased") (1 3 =0 "increased/nochange") (0=.) (.=.), gen (CommFAMILYdecreased2)
tab COMM_FAM_DURING CommFAMILYdecreased2, m

***Composite - communication with friends and/or family decreased***

* Create composite variable for decreased communication with friends or family
```

```
gen DecreasedCommunication = 0 // Initialize variable with 0
replace DecreasedCommunication = 1 if CommFRNDSdecreased == 1 | CommFAMILYdecreased == 1

* Label the new variable for clarity
label variable DecreasedCommunication "Decreased communication with friends or family during COVID-19"

* Check the distribution of the new composite variable
tab DecreasedCommunication
codebook DecreasedCommunication

recode DecreasedCommunication (1=1 "Decreased") (0=0 "other")(.=.), gen (dumDecreasedComm2)
tab DecreasedCommunication dumDecreasedComm2, m

recode DecreasedCommunication (1=0 "Other") (0=1 "Decreased")(.=.), gen (dumNOTDecreasedComm2)
tab DecreasedCommunication dumNOTDecreasedComm2, m

**Quality of sleep**

recode SLEEP_DURING (1=1 "increased") (2 3 =0 "Other") (0=.) (.=.), gen (QualitySLEEPIncreased)
tab SLEEP_DURING QualitySLEEPIncreased, m

recode SLEEP_DURING (2=1 "decreased") (1 3 =0 "Other") (0=.) (.=.), gen (QualitySLEEPdecreased)
tab SLEEP_DURING QualitySLEEPdecreased, m

recode SLEEP_DURING (3 =1 "No Change") (1 2 =0 "Other") (0=.) (.=.), gen (NoChangeInSLEEP)
tab SLEEP_DURING NoChangeInSLEEP, m

recode SLEEP_DURING (2=1 "Decrease") (1 3 =0 "Increase/NoChange") (0 =.) (.=.), gen (dumSLEEP_DECREASEinCOV1)
tab SLEEP_DURING dumSLEEP_DECREASEinCOV1, m

recode SLEEP_DURING (1 3=1 "Increased/NoChange") (2=0 "Decreased") (0 =.) (.=.), gen (dumSLEEP_INCREASE_noCHangeinCOV)
tab SLEEP_DURING dumSLEEP_INCREASE_noCHangeinCOV, m

*****************************************************************************************
**CHARACTERISTICS**
*****************************************************************************************

```

```
*PARTNER TYPE

recode PARTNER_TYPE (1 3 6 =1 "Wife/GF/CLWif") (2 4 5 7 =0 "Other") (0 =.) (.=.), gen (dumFEMALEPartner)
tab PARTNER_TYPE dumFEMALEPartner, m

recode PARTNER_TYPE (2 4 5 =1 "Husband/BF/CLHud") (1 3 6 7 =0 "Other") (0=.) (.=.), gen (dumMALEPartner)
tab PARTNER_TYPE dumMALEPartner, m

recode PARTNER_TYPE (7 =1 "PrefNotSay") (1/6 =0 "Other") (0=.) (.=.), gen (dumUnknownPartner)
tab PARTNER_TYPE dumUnknownPartner, m

tab PARTNER_TYPE GENDER_PARTNER, m

**AGE****

*Participant*

recode AGE_PART (1 2=1 "18-34") (3/6=0 "Other")(0=.) (.=.), gen (dumAgePARTIC_18_34)
tab AGE_PART dumAgePARTIC_18_34, m

recode AGE_PART (3 4=1 "35-54") (1 2 5 6=0 "Other")(0=.) (.=.), gen (dumAgePARTIC_35_54)
tab AGE_PART dumAgePARTIC_35_54, m

recode AGE_PART (5 6=1 "55+") (1 2 3 4 =0 "Other")(0=.) (.=.), gen (dumAgePARTIC_55Plus)
tab AGE_PART dumAgePARTIC_55Plus, m

*Partner*

recode AGE_PARTNER (1 2=1 "18-34") (3/6=0 "Other")(7=.)(0=.)(.=.), gen (dumAgePARTNER_18_34)
tab AGE_PARTNER dumAgePARTNER_18_34, m

recode AGE_PARTNER (3 4=1 "35-54") (1 2 5 6=0 "Other")(7=.)(0=.) (.=.), gen (dumAgePARTNER_35_54)
tab AGE_PARTNER dumAgePARTNER_35_54, m

recode AGE_PARTNER (5 6=1 "55+") (1 2 3 4=0 "Other")(7=.)(0=.) (.=.), gen (dumAgePARTNER_55Plus)
tab AGE_PARTNER dumAgePARTNER_55Plus, m

**RACE***
```

```
*Participant race shortened*

recode RACE_PARTICIPANT_2 (1 3 4 5 6 7 8 9 13 = 1 "Racialized/Indigenous") (2 10 11 = 0 "other")(0=.) (.=.), gen ( dumRacializedPARTIC)
tab RACE_PARTICIPANT_2 dumRacializedPARTIC, m

recode RACE_PARTICIPANT_2 (2=1 "White/Caucasian") (1 3 4 5 6 7 8 9 10 11 13 =0 "Other") (0=.) (.=.), gen (dumWHITEPARTIC)
tab RACE_PARTICIPANT_2 dumWHITEPARTIC, m

recode RACE_PARTICIPANT_2 (10 11 =1 "PrefNotSay") (1 2 3 4 5 6 7 8 9 13=0 "Other") (0=.) (.=.), gen (dumPrefNotSayRACEPARTIC)
tab RACE_PARTICIPANT_2 dumPrefNotSayRACEPARTIC, m

**Race partner shortened*

recode RACE_PARTNER_2 (1 3 4 5 6 7 8 9 13 =1 "Racialized/Indigenous") (2 10 11 =0 "Other") (0=.) (.=.), gen (dumRacializedPARTNER)
tab RACE_PARTNER_2 dumRacializedPARTNER, m

recode RACE_PARTNER_2 (2=1 "White/Caucasian") (1 3 4 5 6 7 8 9 10 11 13 =0 "Other") (0=.) (.=.), gen (dumWHITEPARTNER)
tab RACE_PARTNER_2 dumWHITEPARTNER, m

recode RACE_PARTNER_2 (10 11 =1 "PrefNotSay") (1 2 3 4 5 6 7 8 9 13=0 "Other") (0=.) (.=.), gen (dumPrefNotSayRACEPARNER)
tab RACE_PARTNER_2 dumPrefNotSayRACEPARNER, m

*Participant race shortened - combine racialized/indigenous/prefer not to say*

recode RACE_PARTICIPANT_2 (1 3 4 5 6 7 8 9 10 11 13 = 1 "Racialized/Indigenous/Pref_not") (2 = 0 "White/Caucasian")(0=.) (.=.), gen (dumRacializedPARTIC2)
tab RACE_PARTICIPANT_2 dumRacializedPARTIC2, m

recode RACE_PARTICIPANT_2 (2=1 "White/Caucasian") (1 3 4 5 6 7 8 9 10 11 13 =0 "Racialized/Indigenous/Pref_not") (0=.) (.=.), gen ( dumWHITEPARTIC2)
tab RACE_PARTICIPANT_2 dumWHITEPARTIC2, m

**Race partner shortened* - combine racialized/indigenous/prefer not to say*

recode RACE_PARTNER_2 (1 3 4 5 6 7 8 9 10 11 13 =1 "Racialized/Indigenous/Pref_not") (2=0 "White") (0=.) (.=.), gen ( dumRacializedPARTNER2)
```

```
tab RACE_PARTNER_2 dumRacializedPARTNER2, m

recode RACE_PARTNER_2 (2=1 "White/Caucasian") (1 3 4 5 6 7 8 9 10 11 13 =0 "Racialized/Indigenous/Pref_not") (0=.) (.=.), gen ( dumWHITEPARTNER2)
tab RACE_PARTNER_2 dumWHITEPARTNER2, m

***CITIZENSHIP STATUS****

*Participant*

*CITIZEN_STATUS
recode CITIZEN_STATUS (1=1 "Canadian Citizen") (2/7=0 "Other")(0=.) (.=.), gen (dumCANADIAN_PARTIC2)
tab CITIZEN_STATUS dumCANADIAN_PARTIC2, m

recode CITIZEN_STATUS (2 3 4 5 6 =1 "Immigrant_PR_Refugee") (1 7=0 "Other")(0=.) (.=.), gen (dumIMMIG_PR_REF_PARTIC2)
tab CITIZEN_STATUS dumIMMIG_PR_REF_PARTIC2, m

recode CITIZEN_STATUS (7=1 "unknown") (1/6=0 "Other")(0=.) (.=.), gen (dumCITIZEN_Uknown_PARTIC2)
tab CITIZEN_STATUS dumCITIZEN_Uknown_PARTIC2, m

*Citizenship Status 3

recode CITIZEN_STATUS (1=1 "Canadian Citizen") (2/6=0 "Other")(7=.)(0 =.) (.=.), gen (dumCANADIAN_PARTIC3)
tab CITIZEN_STATUS dumCANADIAN_PARTIC3, m

recode CITIZEN_STATUS (2 3 4 5 6 =1 "Immigrant_PR_Refugee") (7=.) (1 = 0 "Other")(0=.) (.=.), gen (dumIMMIG_PR_REF_PARTIC3)
tab CITIZEN_STATUS dumIMMIG_PR_REF_PARTIC3, m


*PARTNER STATUS

recode STATUS_PARTNER (1=1 "Canadian Citizen") (2/7=0 "Other")(0=.) (.=.), gen (dumCANADIAN_PARTNER2)
tab STATUS_PARTNER dumCANADIAN_PARTNER2, m

recode STATUS_PARTNER (2 3 4 5 6 =1 "Immigrant_PR_Refugee") (1 7=0 "Other")(0=.) (.=.), gen (dumIMMIG_PR_REF_PARTNER2)
tab STATUS_PARTNER dumIMMIG_PR_REF_PARTNER2, m

recode STATUS_PARTNER (7=1 "unknown") (1/6=0 "Other")(0=.)(.=.), gen ( dumCITIZEN_Uknown_PARTNER2)
tab STATUS_PARTNER dumCITIZEN_Uknown_PARTNER2, m
```

```
*Citizenship Status 3

recode STATUS_PARTNER (1=1 "Canadian Citizen") (2/6=0 "Other")(7=.)(0=.) (.=.), gen (dumCANADIAN_PARTNER3)
tab STATUS_PARTNER dumCANADIAN_PARTNER3, m

recode STATUS_PARTNER (2 3 4 5 6 =1 "Immigrant_PR_Refugee") (1=0 "Other")(7=.)(0=.)(.=.), gen (dumIMMIG_PR_REF_PARTNER3)
tab STATUS_PARTNER dumIMMIG_PR_REF_PARTNER3, m

**EDUCATION**

*Education2_Participant*

recode EDUC_PART (1 2=1 "Primary&Secondary") (3/8=0 "Other")(0=.) (.=.), gen (dumPrimSEcEd_PARTIC2)
tab EDUC_PART dumPrimSEcEd_PARTIC2, m

recode EDUC_PART (3 4 5=1 "Trade/College") (1 2 6 7 8=0 "Other")(0=.) (.=.), gen (dumTrade_DiplomaEd_PARTIC2)
tab EDUC_PART dumTrade_DiplomaEd_PARTIC2, m

recode EDUC_PART (6 7 8=1 "University or higher") (1/5=0 "Other")(0=.) (.=.), gen (dumUniversityEd_PARTIC2)
tab EDUC_PART dumUniversityEd_PARTIC2, m

*Education Partner 2*

recode EDUC_PARTNER (1 2=1 "Primary&Secondary") (3/9=0 "Other")(0=.) (.=.), gen (dumPrimSEcEd_PARTNER2)
tab EDUC_PARTNER dumPrimSEcEd_PARTNER2, m

recode EDUC_PARTNER (3 4 5=1 "Trade/College") (1 2 6 7 8 9=0 "Other")(0=.) (.=.), gen (dumTrade_DiplomaEd_PARTNER2)
tab EDUC_PARTNER dumTrade_DiplomaEd_PARTNER2, m

recode EDUC_PARTNER (6 7 8=1 "University or higher") (1 2 3 4 5 9=0 "Other")(0=.) (.=.), gen (dumUniversityEd_PARTNER2)
tab EDUC_PARTNER dumUniversityEd_PARTNER2, m

recode EDUC_PARTNER (9=1 "UnKnown") (1/8=0 "Other")(0=.) (.=.), gen (dumUnknownED_PARTNER2)
tab EDUC_PARTNER dumUnknownED_PARTNER2, m

*Education Partner 3*

recode EDUC_PARTNER (1 2=1 "Primary&Secondary") (3/8=0 "Other")(9=.)(0
```

```
recode EDUC_PARTNER (1 2=1 "Primary&Secondary") (3/8=0 "Other")(9=.)(0=.) (.=.), gen (dumPrimSEcEd_PARTNER3)
tab EDUC_PARTNER dumPrimSEcEd_PARTNER3, m

recode EDUC_PARTNER (3 4 5=1 "Trade/College") (1 2 6 7 8=0 "Other")(9=.)(0=.) (.=.), gen (dumTrade_DiplomaEd_PARTNER3)
tab EDUC_PARTNER dumTrade_DiplomaEd_PARTNER3, m

recode EDUC_PARTNER (6 7 8=1 "University or higher") (1 2 3 4 5 =0 "Other")(9=.)(0=.) (.=.), gen (dumUniversityEd_PARTNER3)
tab EDUC_PARTNER dumUniversityEd_PARTNER3, m

**EMPLOYMENT**

*Employed during COVID*

*Participant
recode EMPL_PART_DURING (1=1 "Yes") (2 3=0 "Other")(0=.) (.=.), gen (dumEMPLOYEDPARTIC)
tab EMPL_PART_DURING dumEMPLOYEDPARTIC, m

recode EMPL_PART_DURING (2=1 "No") (1 3=0 "Other")(0=.) (.=.), gen (dumUNEMPLOYEDPARTIC)
tab EMPL_PART_DURING dumUNEMPLOYEDPARTIC, m

recode EMPL_PART_DURING (3=1 "Retired") (1 2=0 "Other")(0=.) (.=.), gen (dumRetiredPARTIC)
tab EMPL_PART_DURING dumRetiredPARTIC, m

*Participant Employment2*

recode EMPL_PART_DURING (1=1 "Yes") (2 3=0 "No & Retired")(0=.) (.=.), gen (dumEMPLOYEDPARTIC2)
tab EMPL_PART_DURING dumEMPLOYEDPARTIC2, m

recode EMPL_PART_DURING (2 3=1 "No & Retired") (1=0 "Yes")(0=.) (.=.), gen (dumUNEMPLOYEDPARTIC2)
tab EMPL_PART_DURING dumUNEMPLOYEDPARTIC2, m

*Partner*
recode EMP_PARTNER_DURING (1=1 "Yes") (2 3 4=0 "Other")(0=.) (.=.), gen (dumEMPLOYEDPARTNER)
tab EMP_PARTNER_DURING dumEMPLOYEDPARTNER, m

recode EMP_PARTNER_DURING (2=1 "No") (1 3 4=0 "Other")(0=.) (.=.), gen (dumUNEMPLOYEDPARTNER)
tab EMP_PARTNER_DURING dumUNEMPLOYEDPARTNER, m
```

```
(.=.), gen (dumRetiredPARTNER)
tab EMP_PARTNER_DURING dumRetiredPARTNER, m

recode EMP_PARTNER_DURING (4=1 "Unknown") (1 2 3=0 "Other")(0=.)
(.=.), gen (dumUNKNOWNEMPLOYEDPARTNER)
tab EMP_PARTNER_DURING dumUNKNOWNEMPLOYEDPARTNER, m

*Partner Emp2*

recode EMP_PARTNER_DURING (1=1 "Yes") (2 3=0 "No& Retired")(4=.)(0=.)
(.=.), gen (dumEMPLOYEDPARTNER2)
tab EMP_PARTNER_DURING dumEMPLOYEDPARTNER2, m

recode EMP_PARTNER_DURING (2 3 =1 "No") (1=0 "Other")(4=.)(0=.)(.=.),
gen (dumUNEMPLOYEDPARTNER2)
tab EMP_PARTNER_DURING dumUNEMPLOYEDPARTNER2, m

*Household Income - unknown is missing (7)*

recode HH_INCOME (1 2=1 ">40,000") (3/6=0 "other") (7=.) (0=.) (.=.),
gen (dumHHINCOMEUNDER40)
tab HH_INCOME dumHHINCOMEUNDER40, m

recode HH_INCOME (3 4 =1 "40 to 69,999") (1 2 5 6 =0 "Other") (7=.) (0
=.) (.=.), gen (dumHHINCOMEUNDER40_69)
tab HH_INCOME dumHHINCOMEUNDER40_69, m

recode HH_INCOME (5=1 "70-99,999") (1 2 3 4 6=0 "Other") (7=.) (0=.)
(.=.), gen (dumHHINCOMEUNDER70_99)
tab HH_INCOME dumHHINCOMEUNDER70_99, m

recode HH_INCOME (6=1 "100,000PLUS") (1 2 3 4 5=0 "Other")(7=.)  (0=.)
 (.=.), gen (dumHHINCOMEUNDER100PLUS)
tab HH_INCOME dumHHINCOMEUNDER100PLUS, m

*GEOGRAPHY

recode GEOGRAPHY (1=1 "Eastern Ontario") (2/6=0 "Other")(0=.) (.=.),
gen (dumEASTONT2)
tab GEOGRAPHY dumEASTONT2, m

recode GEOGRAPHY (2=1 "Central Ontario") (1 3 4 5 6 7 8 9=0 "Other")(0
=.) (.=.), gen (dumCENTRALONT2)
tab GEOGRAPHY dumCENTRALONT2, m

recode GEOGRAPHY (3 4=1 "Toronto &GTA") (1 2 5 6=0 "Other")(0=.)
(.=.), gen (dumToronto_GTA2)
tab GEOGRAPHY dumToronto_GTA2, m
```

```
recode GEOGRAPHY (5=1 "Western Ontario") (1 2 3 4 6 =0 "Other")(0=.) (.=.), gen (dumWESTONT2)
tab GEOGRAPHY dumWESTONT2, m

recode GEOGRAPHY (6=1 "Northern Ontario") (1/5=0 "Other")(0=.) (.=.), gen (dumNORTHONT2)
tab GEOGRAPHY dumNORTHONT2, m

*Rural_Urban*

recode RURALURBAN (1=1 "Rural") (2=0 "Urban")(0=.) (.=.), gen ( dumRURAL)
tab RURALURBAN dumRURAL, m

recode RURALURBAN (2=1 "Urban") (1=0 "Rural")(0=.) (.=.), gen ( dumURBAN)
tab RURALURBAN dumURBAN, m


******OTHER FACTORS KNOWN TO IMPACT IPV***********

**DRINK-DRUGS-SOURCE OF STRAIN**

recode WISH_DRINK_COMBINED (3 4 5=1 "Always/Often/Sometimes") (1 2 =0 "Never/Seldom") (0=.) (.=.), gen (dumWishNoDrink)
tab WISH_DRINK_COMBINED dumWishNoDrink, m

recode WISH_DRINK_COMBINED (1 2=1 "Never/Seldom") (3 4 5=0 "Always/Often/Sometimes") (0=.) (.=.), gen (dumWishDrinkNEVER)
tab WISH_DRINK_COMBINED dumWishDrinkNEVER, m

recode STRAIN_DRINK_COMBINED (3 4 5=1 "Always/Often/Sometimes") (1 2 = 0 "Never/Seldom") (0=.) (.=.), gen (dumSourceStrainDrink)
tab STRAIN_DRINK_COMBINED dumSourceStrainDrink, m

recode STRAIN_DRINK_COMBINED (1 2=1 "Never/Seldom") (3 4 5=0 "Always/Often/Sometimes") (0=.) (.=.), gen (dumNO_SourceStrainDrink)
tab STRAIN_DRINK_COMBINED dumNO_SourceStrainDrink, m

recode CON_LEAVE_DRINK_COMBINED (3 4 5=1 "Always/Often/Sometimes") (1 2 =0 "Never/Seldom") (0=.) (.=.), gen (dumConsLeaveDrink)
tab CON_LEAVE_DRINK_COMBINED dumConsLeaveDrink, m

recode CON_LEAVE_DRINK_COMBINED (1 2=1 "Never/Seldom") (3 4 5=0 "Always/Often/Sometimes") (0=.) (.=.), gen (dumNO_ConsLeaveDrink)
tab CON_LEAVE_DRINK_COMBINED dumNO_ConsLeaveDrink, m
```

```
recode WISH_DRUGS_COMBINED (3 4 5=1 "Always/Often/Sometimes") (1 2 =0 "Never/Seldom") (0=.) (.=.), gen (dumWishNoDrugs)
tab WISH_DRUGS_COMBINED dumWishNoDrugs, m

recode WISH_DRUGS_COMBINED (1 2=1 "Never/Seldom") (3 4 5=0 "Always/Often/Sometimes") (0=.) (.=.), gen (dumWishNoDrugsNEVER)
tab WISH_DRUGS_COMBINED dumWishNoDrugsNEVER, m

recode STRAIN_DRUGS_COMBINED (3 4 5=1 "Always/Often/Sometimes") (1 2 = 0 "Never/Seldom") (0=.) (.=.), gen (dumSourceStrainDrugs)
tab STRAIN_DRUGS_COMBINED dumSourceStrainDrugs, m

recode STRAIN_DRUGS_COMBINED (1 2=1 "Never/Seldom") (3 4 5=0 "Always/Often/Sometimes") (0=.) (.=.), gen (dumNO_SourceStrainDrugs)
tab STRAIN_DRUGS_COMBINED dumNO_SourceStrainDrugs, m

recode CON_LEAVE_DRUGS_COMBINED (3 4 5=1 "Always/Often/Sometimes") (1 2 =0 "Never/Seldom") (0=.) (.=.), gen (dumConsLeaveDrugs)
tab CON_LEAVE_DRUGS_COMBINED dumConsLeaveDrugs, m

recode CON_LEAVE_DRUGS_COMBINED (1 2=1 "Never/Seldom") (3 4 5=0 "Always/Often/Sometimes") (0=.) (.=.), gen (dumNO_ConsLeaveDrugs)
tab CON_LEAVE_DRUGS_COMBINED dumNO_ConsLeaveDrugs, m

* Sum the binary variables to create a composite score
gen substanceIssueScore = dumWishNoDrink + dumSourceStrainDrink + dumConsLeaveDrink + dumWishNoDrugs + dumSourceStrainDrugs + dumConsLeaveDrugs

* Define the impact level based on the sum score
gen substanceImpactLevel = "Low Impact" if substanceIssueScore <= 2
replace substanceImpactLevel = "Moderate Impact" if substanceIssueScore >= 3 & substanceIssueScore <= 4
replace substanceImpactLevel = "High Impact" if substanceIssueScore >= 5

* Tabulate the new categorized variable
tabulate substanceImpactLevel
encode substanceImpactLevel, generate(substanceImpactLevel_encoded)
tab substanceImpactLevel_encoded,m

recode substanceImpactLevel_encoded (1=1 "high impact" ) (2/4 =0 "Other")(0=.) (.=.), gen (dumSubstanceHIGHIMPACT)
tab substanceImpactLevel_encoded dumSubstanceHIGHIMPACT, m

recode substanceImpactLevel_encoded (2=1 "Low impact" ) (1 3 4 =0 "Other")(0=.) (.=.), gen (dumSubstanceLOWIMPACT)
tab substanceImpactLevel_encoded dumSubstanceLOWIMPACT, m
```

```
recode substanceImpactLevel_encoded (3=1 "Moderate impact" ) (1 2 4 =0 "Other")(0=.) (.=.), gen (dumSubstanceModerateIMPACT)
tab substanceImpactLevel_encoded dumSubstanceModerateIMPACT, m

***OR - SOURCE OF STRAIN ***
codebook substanceImpactLevel, m
codebook substanceImpactLevel_encoded, m

recode substanceImpactLevel_encoded (1 3 = 1 "moderate/high") (2 = 0 "low")(0=.) (.=.), gen (dumSubstanceModHighIMPACT2)
tab substanceImpactLevel_encoded dumSubstanceModHighIMPACT2, m

recode substanceImpactLevel_encoded (2 = 1 "low") (1 3 = 0 "moderate/high")(0=.) (.=.), gen (dumSubstanceLowIMPACT2)
tab substanceImpactLevel_encoded dumSubstanceLowIMPACT2, m


**************************
*Information_during_COVID
**************************

recode INFORMATION_DURING (1=1 "yes") (2 3=0 "Other") (0=.) (.=.), gen (dumYESINFORMATION)
tab INFORMATION_DURING dumYESINFORMATION, m

recode INFORMATION_DURING (2=1 "No") (1 3=0 "Other") (0=.) (.=.), gen (dumNOINFORMATION)
tab INFORMATION_DURING dumNOINFORMATION, m

recode INFORMATION_DURING (3=1 "I don't know") (1 2=0 "Other") (0=.) (.=.), gen (dumNOSUREInformation)
tab INFORMATION_DURING dumNOSUREInformation, m

*Information 2

recode INFORMATION_DURING (1=1 "yes") (2 3=0 "No_dontknow") (0=.) (.=.), gen (dumYESINFORMATION2)
tab INFORMATION_DURING dumYESINFORMATION2, m

recode INFORMATION_DURING (2 3=1 "No_Dontknow") (1=0 "Yes") (0=.) (.=.), gen (dumNOINFORMATION2)
tab INFORMATION_DURING dumNOINFORMATION2, m

**************************
*Community Violence*
**************************
```

```
recode COMM_VIOL_DURING (1=1 "No") (2 3=0 "Other")(0=.) (.=.), gen (dumNO_COMMVIOLENCE)
tab COMM_VIOL_DURING dumNO_COMMVIOLENCE, m

recode COMM_VIOL_DURING (2=1 "Yes") (1 3=0 "Other")(0=.) (.=.), gen (dumYES_COMMVIOLENCE)
tab COMM_VIOL_DURING dumYES_COMMVIOLENCE, m

recode COMM_VIOL_DURING (3=1 "Not Sure") (1 2=0 "Other")(0=.) (.=.), gen (dumNotSure_COMMVIOLENCE)
tab COMM_VIOL_DURING dumNotSure_COMMVIOLENCE, m

**rolled up no and not sure

recode COMM_VIOL_DURING (1 3=1 "No") (2=0 "Yes")(0=.) (.=.), gen (dumNO_COMMVIOLENCE2)
tab COMM_VIOL_DURING dumNO_COMMVIOLENCE2, m

recode COMM_VIOL_DURING (2=1 "Yes") (1 3=0 "No")(0=.) (.=.), gen (dumYES_COMMVIOLENCE2)
tab COMM_VIOL_DURING dumYES_COMMVIOLENCE2, m


**Caregiving NUM_CHILD

recode NUM_CHILD (1 = 1 "None") (2/6 = 0 "Other") (0=.) (.=.), gen (dumNoChildren)
tab NUM_CHILD dumNoChildren, m

recode NUM_CHILD (2 3 4 = 1 "1 to 3") (1 5 6 = 0 "Other") (0=.) (.=.), gen (dum1_3_Children)
tab NUM_CHILD dum1_3_Children, m

recode NUM_CHILD (5 6 = 1 "4 +") (1 2 3 4 = 0 "Other") (0=.) (.=.), gen (dum4PlusChildren)
tab NUM_CHILD dum4PlusChildren, m

*NUM_CHILD_COMBINED

recode NUM_CHILD (1 = 1 "No_Children") (2/6 = 0 "Yes_Children") (0=.) (.=.), gen (dumNoChildren2)
tab NUM_CHILD dumNoChildren2, m

recode NUM_CHILD (2 3 4 5 6 = 1 "Yes_Children") (1 = 0 "No_Children") (0=.) (.=.), gen (dumYes_Children)
tab NUM_CHILD dumYes_Children, m
```

```
*PRIMARY_CAREGIVER_DURING

codebook PRIMARY_CAREGIVER_DURING
codebook CAREGIVING_nonCHILD

recode PRIMARY_CAREGIVER_DURING (1=1 "yes") (2=0 "No") (0=.) (.=.), 
gen (dumCaregiverChiild)
tab PRIMARY_CAREGIVER_DURING dumCaregiverChiild, m

recode PRIMARY_CAREGIVER_DURING (2=1 "No") (1=0 "Yes") (0=.) (.=.), 
gen (dumNotCaregiverChiild)
tab PRIMARY_CAREGIVER_DURING dumNotCaregiverChiild, m

recode CAREGIVING_nonCHILD (1=1 "yes") (2=0 "No") (0=.) (.=.), gen (
dumCaregiverNonChiild)
tab CAREGIVING_nonCHILD dumCaregiverNonChiild, m

recode CAREGIVING_nonCHILD (2=1 "No") (1=0 "Yes") (0=.) (.=.), gen (
dumNotCaregiverNonChiild)
tab CAREGIVING_nonCHILD dumNotCaregiverNonChiild, m

* Combine into a single caregiver variable
gen dumCaregiver = (dumCaregiverChiild == 1 | dumCaregiverNonChiild ==
 1)
label define caregiverlabel 0 "No" 1 "Yes"
label values dumCaregiver caregiverlabel

tab dumCaregiverChiild  dumCaregiverNonChiild
* Tabulate to check the new variable
tab dumCaregiver,m
codebook dumCaregiver

recode dumCaregiver (1=1 "yes") (0=0 "No") (.=.), gen (dumCaregiverYES
)
tab dumCaregiver dumCaregiverYES, m

recode dumCaregiver (0=1 "No") (1=0 "Yes") (.=.), gen (dumCaregiverNO)
tab dumCaregiver dumCaregiverNO, m

****GENDER ANALYSIS*******

***********************************
*****SOCIODEMOGRAPHIC DATA*********
***********************************

*Gender*

*Participant
```

```
*Participant
codebook GENDER_PARTICIPANT

recode GENDER_PARTICIPANT (1=1 "Woman") (2 3 4 6 5 7 8 =0 "Other") (0 =.) (.=.), gen (dumWoman)
tab GENDER_PARTICIPANT dumWoman, m

recode GENDER_PARTICIPANT (2=1 "Man") (1 3 4 5 6 7 8=0 "Other") (0=.) (.=.), gen (dumMan)
tab GENDER_PARTICIPANT dumMan, m

recode GENDER_PARTICIPANT (3 4 5 6 7 8=1 "Gender Diverse/PreferNotSay" ) (1 2=0 "Other") (0=.) (.=.), gen (dumGenders)
tab GENDER_PARTICIPANT dumGenders, m

*Participant 2 - with out prefer not to say

recode GENDER_PARTICIPANT (1=1 "Woman") (2 3 4 6 5 7 =0 "Other") (8 =.)(0=.) (.=.), gen (dumWoman2)
tab GENDER_PARTICIPANT dumWoman2, m

recode GENDER_PARTICIPANT (2=1 "Man") (1 3 4 5 6 7 =0 "Other")(8=.) (0 =.) (.=.), gen (dumMan2)
tab GENDER_PARTICIPANT dumMan2, m

recode GENDER_PARTICIPANT (3 4 5 6 7 =1 "genderdiverse") (1 2=0 "Other") (8=.)(0=.) (.=.), gen (dumGenders2)
tab GENDER_PARTICIPANT dumGenders2, m

**Participant 3 - women and men

recode GENDER_PARTICIPANT (1=1 "Woman") (2 =0 "Man")(3 =.)(4 =.)(5=.)(6=.)(7=.)(8=.)(0=.)(.=.), gen (dumWoman3)
tab GENDER_PARTICIPANT dumWoman3, m

recode GENDER_PARTICIPANT (2=1 "Man") (1=0 "Woman")(3 =.)(4 =.)(5=.)(6 =.)(7=.)(8=.)(0=.)(.=.), gen (dumMan3)
tab GENDER_PARTICIPANT dumMan3, m

*Partner
recode GENDER_PARTNER (2=1 "Woman") (1 3 4 5 6 7 8 =0 "Other") (0=.) (.=.), gen (dumWomanPartner)
tab GENDER_PARTNER dumWomanPartner, m

recode GENDER_PARTNER (1=1 "Man") (2 3 4 5 6 7 8=0 "Other") (0=.) (.=.), gen (dumManPartner)
tab GENDER_PARTNER dumManPartner, m
```

```
recode GENDER_PARTNER (3 4 5 6 7 8=1 "GenderDiverse/PreferNotSay") (1 2=0 "Other") (0=.) (.=.), gen (dumGendersPartner)
tab GENDER_PARTNER dumGendersPartner, m

*Partner 2 without prefer not to say

recode GENDER_PARTNER (2=1 "Woman") (1 3 4 5 6 7 =0 "Other") (8=.)(0 =.)(.=.), gen (dumWomanPartner2)
tab GENDER_PARTNER dumWomanPartner2, m

recode GENDER_PARTNER (1=1 "Man") (2 3 4 5 6 7 =0 "Other")(8=.)(0 =.)(.=.), gen (dumManPartner2)
tab GENDER_PARTNER dumManPartner2, m

recode GENDER_PARTNER (3 4 5 6 7 =1 "genderdiverse") (1 2=0 "Man/Woman")(8=.)(0=.) (.=.), gen (dumGendersPartner2)
tab GENDER_PARTNER dumGendersPartner2, m

**Partner 3 - just women and men

recode GENDER_PARTNER (2=1 "Woman") (1 =0 "Man")(3 =.)(4 =.)(5=.)(6 =.)(7=.)(8=.)(0=.)(.=.), gen (dumWomanPartner3)
tab GENDER_PARTNER dumWomanPartner3, m

recode GENDER_PARTNER (1=1 "Man") (2=0 "Woman")(3 =.)(4 =.)(5=.)(6=.)(7=.)(8=.)(0=.) (.=.), gen (dumManPartner3)
tab GENDER_PARTNER dumManPartner3, m

***Partner 4 - women and gender diverse

recode GENDER_PARTNER (2 3 4 5 6 7 =1 "Woman_Gender Diverse") (1 =0 "Man") (8=.)(0=.)(.=.), gen (dumWomanGDPartner4)
tab GENDER_PARTNER dumWomanGDPartner4, m

recode GENDER_PARTNER (1=1 "Man") (2 3 4 5 6 7 =0 "Woman_Gender Diverse")(8=.)(0=.)(.=.), gen (dumManPartner4)
tab GENDER_PARTNER dumManPartner4, m

***** GENDER AND RELATIONSHIP TYPE ANALYSIS ****

* Step 1: Create a new variable for participant gender
gen new_genderParticipant = ""
replace new_genderParticipant = "woman" if GENDER_PARTICIPANT == 1
replace new_genderParticipant = "man" if GENDER_PARTICIPANT == 2
replace new_genderParticipant = "gender diverse" if inlist(GENDER_PARTICIPANT, 3, 4, 5, 6, 7)
replace new_genderParticipant = "prefer not to say" if GENDER_PARTICIPANT == 8
```

```
replace new_genderParticipant = "missing" if GENDER_PARTICIPANT == 0 | missing(GENDER_PARTICIPANT)

* Step 2: Create a new variable for partner gender
gen new_genderPartner = ""
replace new_genderPartner = "woman" if GENDER_PARTNER == 2
replace new_genderPartner = "man" if GENDER_PARTNER == 1
replace new_genderPartner = "gender diverse" if inlist(GENDER_PARTNER, 3, 4, 5, 6, 7)
replace new_genderPartner = "prefer not to say" if GENDER_PARTNER == 8
replace new_genderPartner = "missing" if GENDER_PARTNER == 0 | missing(GENDER_PARTNER)

* Step 3: Create a new variable for relationship type
gen relationship_type3 = .

* Step 4: Categorize heterosexual relationships as 1
replace relationship_type3 = 1 if (new_genderParticipant == "man" & new_genderPartner == "woman") | ///
                                 (new_genderParticipant == "woman" & new_genderPartner == "man")

* Step 5: Categorize same-sex relationships as 2
replace relationship_type3 = 2 if (new_genderParticipant == "man" & new_genderPartner == "man") | ///
                                 (new_genderParticipant == "woman" & new_genderPartner == "woman")

* Step 6: Categorize gender-diverse relationships as 3
replace relationship_type3 = 3 if (new_genderParticipant == "gender diverse" | new_genderPartner == "gender diverse")

* Step 7: Categorize "prefer not to say" as 4
replace relationship_type3 = 4 if (new_genderParticipant == "prefer not to say" | new_genderPartner == "prefer not to say")

* Step 8: Handle missing cases (keep as missing in relationship_type3)
replace relationship_type3 = . if (new_genderParticipant == "missing" | new_genderPartner == "missing")

* Step 9: Verify the distribution of relationship types
tabulate relationship_type3

* Step 10: Perform chi-square analysis for relationship type and IPV experience
*tabulate relationship_type3 dumYES_IPV, chi2 row
*tabulate relationship_type3 dumYES_IPV, exact row
```

```
*tabulate relationship_type3 dumYES_IPV, exact row

* Step 11: Perform chi-square analysis for gender and IPV experience
*tabulate new_genderParticipant dumYES_IPV, chi2
*tabulate new_genderParticipant dumYES_IPV, exact

*tabulate relationship_type3 dumYES_IPV, chi2 row
*tabulate relationship_type3 dumYES_IPV, exact row

* Subset the data for "Prefer not to say" (relationship_type3 == 4)
*tabulate dumYES_IPV if relationship_type3 == 4, chi2 row
*tabulate dumYES_IPV if relationship_type3 == 4, exact row

codebook relationship_type3,m

recode relationship_type3 (1 = 1 "heterosexual") (2 3 4 = 0 "other") (.=.) (0=.), gen (dumheterosexual)
tab relationship_type3 dumheterosexual, m

recode relationship_type3 (2 3 = 1 "LGBTQ2+") (1 4 = 0 "other") (.=.) (0=.), gen (dumLGBTQ2)
tab relationship_type3 dumLGBTQ2, m

recode relationship_type3 (4 = 1 "PreferNotSay") (1 2 3 = 0 "other") (.=.) (0=.), gen (dumRelationprefnotsay)
tab relationship_type3 dumRelationprefnotsay, m

ttest dumheterosexual, by (dumYES_IPV)
ttest dumLGBTQ2, by (dumYES_IPV)
ttest dumRelationprefnotsay, by (dumYES_IPV)
ranksum dumRelationprefnotsay, by (dumYES_IPV)
tabulate dumRelationprefnotsay dumYES_IPV, exact

**AD HOC**
* Fisher's Exact Test: Heterosexual vs. Same-Sex
tabulate dumYES_IPV relationship_type3 if relationship_type3 == 1 | relationship_type3 == 2, exact

* Fisher's Exact Test: Heterosexual vs. Gender-Diverse
tabulate dumYES_IPV relationship_type3 if relationship_type3 == 1 | relationship_type3 == 3, exact

* Fisher's Exact Test: Heterosexual vs. Prefer Not to Say
tabulate dumYES_IPV relationship_type3 if relationship_type3 == 1 | relationship_type3 == 4, exact

* Same-Sex vs. Gender-Diverse
tabulate dumYES_IPV relationship_type3 if relationship_type3 == 2 |
```

```
relationship_type3 == 3, exact

* Same-Sex vs. Prefer Not to Say
tabulate dumYES_IPV relationship_type3 if relationship_type3 == 2 | relationship_type3 == 4, exact

* Gender-Diverse vs. Prefer Not to Say
tabulate dumYES_IPV relationship_type3 if relationship_type3 == 3 | relationship_type3 == 4, exact


* Step 12: Create a binary variable for relationship type
gen binary_relationship_type = .

* Categorize Heterosexual relationships as 1
replace binary_relationship_type = 1 if relationship_type3 == 1

* Categorize LGBTQ2+/Unspecified relationships (Same-Sex, Gender-Diverse, and Prefer Not to Say) as 2
replace binary_relationship_type = 2 if inlist(relationship_type3, 2, 3, 4)

* Handle missing cases
replace binary_relationship_type = . if missing(relationship_type3)

* Step 13: Verify the distribution of the binary variable
tabulate binary_relationship_type
codebook binary_relationship_type, m

recode binary_relationship_type (1 = 1 "Heterosexual") (2 = 0 "LGBTQ2") (.=.) (0=.) , gen (dumHeterosexual2)
tab binary_relationship_type dumHeterosexual2, m

recode binary_relationship_type (2 = 1 "LGBTQ2") (1 = 0 "Heterosexual") (.=.) (0=.), gen (dumLGBTQ2b)
tab binary_relationship_type dumLGBTQ2b, m

***********************************
*****DESCRIPTIVES*********
***********************************

*No, a t-test does not work in this case because: Most of your variables are categorical, not continuous. A t-test compares means of continuous variables between two groups, but your variables represent categories (e.g., gender, education, race, employment status, etc.), which are not measured on a numerical scale.
*Age and income are in categories, not as continuous values. If you had continuous age and income, a t-test could work.
```

```
*Since your data presents age and income in groups (e.g., 18–34, 35–54), you should use Chi-square or Fisher's exact test instead of a t-test.
*If you do have raw age and income values elsewhere, you could use the Wilcoxon rank-sum test (Mann-Whitney U test) instead of a t-test because it does not assume normality.
*Correct Statistical Approach
*Chi-square test → For categorical variables (most of your table).
*Fisher's exact test → For categorical variables with small expected counts.
*Wilcoxon rank-sum test (Mann-Whitney U) → If you have continuous age/income.
*Research question*
*How is the experience of intimate partner violence (IPV) during COVID-19 in Ontario associated with barriers to accessing formal and informal supports?*
***because some of the categories had small sample size, we had to test using different methods for significance:
*Chi square
tabulate variable group, chi2
*Fisher's exact
tabulate variable group, exact
*ranksum
ranksum variable, by(group)
*Gender**
codebook GENDER_PARTICIPANT
gen participant_gender = .
replace participant_gender = 1 if inlist(GENDER_PARTICIPANT, 1)  // Woman
replace participant_gender = 2 if inlist(GENDER_PARTICIPANT, 2)  // Man
replace participant_gender = 3 if inlist(GENDER_PARTICIPANT, 3, 4, 5, 6, 7, 8)  // Gender Diverse
label define partgender_labels 1 " Woman" 2 " Man" 3 "Gender Diverse"
label values participant_gender partgender_labels
tab GENDER_PARTICIPANT participant_gender, m
tabulate participant_gender dumYES_IPV, chi2
*Age Participant*
codebook AGE_PART
gen age_group = .
replace age_group = 1 if inlist(AGE_PART, 1, 2)  // 18–34
replace age_group = 2 if inlist(AGE_PART, 3, 4)  // 35–54
replace age_group = 3 if inlist(AGE_PART, 5, 6)  // 55+
```

```
replace age_group = 3 if inlist(AGE_PART, 5, 6)  // 55+
label define age_labels 1 "18-34" 2 "35-54" 3 "55+"
label values age_group age_labels
tab AGE_PART age_group, missing

tabulate age_group dumYES_IPV, chi2

*Relationship Type*
codebook relationship_type3
gen relationship = .
replace relationship = 1 if inlist(relationship_type3, 1)  // Heterosexual
replace relationship = 2 if inlist(relationship_type3, 2, 3)  // LGBTQ2+
replace relationship = 3 if inlist(relationship_type3, 4)  // Prefer not to say
label define relationship_labels 1 "Heterosexual" 2 "LGBTQ2+" 3 "NotDisclosed"
label values relationship relationship_labels
tab relationship_type3 relationship, m

tabulate relationship dumYES_IPV, exact

*Race participant*

codebook RACE_PARTICIPANT_2
gen race_participant = .
replace race_participant = 1 if inlist(RACE_PARTICIPANT_2, 1, 3, 4, 5, 6, 7, 8, 9, 13)  // racialized
replace race_participant = 2 if inlist(RACE_PARTICIPANT_2, 2)  // white
replace race_participant = 3 if inlist(RACE_PARTICIPANT_2, 10, 11) // Prefer not to say
label define raceparticipant_labels 1 "racialized" 2 "white" 3 "NotDisclosed"
label values race_participant raceparticipant_labels
tab RACE_PARTICIPANT_2 race_participant, m
tabulate race_participant dumYES_IPV, chi2

*Immigration Status
codebook CITIZEN_STATUS
gen participant_citizenship = .
replace participant_citizenship = 1 if inlist(CITIZEN_STATUS, 1)  // Canadian
replace participant_citizenship = 2 if inlist(CITIZEN_STATUS, 2, 3, 4, 5, 6)  // Immigrant/Refugee/PR
replace participant_citizenship = 3 if inlist(CITIZEN_STATUS, 7)  // Prefer not to say
```

```
label define citizenshipparticipant_labels 1 "Canadian" 2 "Immigrant/Refugee/PR" 3 "Prefer not to say"
label values participant_citizenship citizenshipparticipant_labels
tab CITIZEN_STATUS participant_citizenship, m

tabulate participant_citizenship dumYES_IPV, exact

codebook CITIZEN_STATUS
gen participant_citizenship2 = .
replace participant_citizenship2 = 1 if inlist(CITIZEN_STATUS, 1)  // Canadian
replace participant_citizenship2 = 2 if inlist(CITIZEN_STATUS, 2, 3, 4, 5, 6)  // Immigrant/Refugee/PR
label define citizenshipparticipant_labels2 1 "Canadian" 2 "Immigrant/Refugee/PR"
label values participant_citizenship2 citizenshipparticipant_labels2
tab CITIZEN_STATUS participant_citizenship2, m

tabulate participant_citizenship2 dumYES_IPV, chi2

**Education participant*

codebook EDUC_PART
gen participant_education = .
replace participant_education = 1 if inlist(EDUC_PART, 1,2)  // Primary/Secondary
replace participant_education = 2 if inlist(EDUC_PART, 3,4,5)  // Trade/College
replace participant_education = 3 if inlist(EDUC_PART, 6,7,8)  // University+
label define participanteduc_labels 1 " Primary/Secondary" 2 " Trade/College" 3 " University+"
label values participant_education participanteduc_labels
tab EDUC_PART participant_education, m

tabulate participant_education dumYES_IPV, chi2

**Employment participant*
codebook EMPL_PART_DURING
gen participant_employment = .
replace participant_employment = 1 if inlist(EMPL_PART_DURING, 1)  // Employed
replace participant_employment = 2 if inlist(EMPL_PART_DURING, 2,3)  // Unemployed
label define participantemp_labels 1 " Employed" 2 " Unemployed"
label values participant_employment participantemp_labels
tab EMPL_PART_DURING participant_employment, m

```

```
tabulate participant_employment dumYES_IPV, chi2

**Partner Gender**
codebook GENDER_PARTNER
gen partner_gender = .
replace partner_gender = 1 if inlist(GENDER_PARTNER, 1)  // Man
replace partner_gender = 2 if inlist(GENDER_PARTNER, 2)  // Woman
replace partner_gender = 3 if inlist(GENDER_PARTNER, 3, 4, 5, 6, 7, 8)  // Gender Diverse
label define partnergender_labels 1 " Man" 2 " Woman" 3 "Gender Diverse"
label values partner_gender partnergender_labels
tab GENDER_PARTNER partner_gender, m

tabulate partner_gender dumYES_IPV, exact

*Age partner*
codebook AGE_PARTNER
gen partner_age_group = .
replace partner_age_group = 1 if inlist(AGE_PARTNER, 1, 2)  // 18-34
replace partner_age_group = 2 if inlist(AGE_PARTNER, 3, 4)  // 35-54
replace partner_age_group = 3 if inlist(AGE_PARTNER, 5, 6)  // 55+
label define partnerage_labels 1 "18-34" 2 "35-54" 3 "55+"
label values partner_age_group partnerage_labels
tab AGE_PARTNER partner_age_group, missing

tabulate partner_age_group dumYES_IPV, chi2

*Partner Race*
codebook RACE_PARTNER_2
gen race_partner = .
replace race_partner = 1 if inlist(RACE_PARTNER_2, 1, 3, 4, 5, 6, 7, 8, 9, 13)  // racialized
replace race_partner = 2 if inlist(RACE_PARTNER_2, 2)  // white
replace race_partner = 3 if inlist(RACE_PARTNER_2, 10, 11)  // Prefer not to say
label define racepartner_labels 1 "racialized" 2 "white" 3 "NotDisclosed"
label values race_partner racepartner_labels
tab RACE_PARTNER_2 race_partner, m

tabulate race_partner dumYES_IPV, chi2

*Partner Education
codebook EDUC_PARTNER
gen partner_education = .
replace partner_education = 1 if inlist(EDUC_PARTNER, 1,2)  // Primary/Secondary
```

```
replace partner_education = 2 if inlist(EDUC_PARTNER, 3,4,5)  // Trade/College
replace partner_education = 3 if inlist(EDUC_PARTNER, 6,7,8)  // University+
label define partnereduc_labels 1 " Primary/Secondary" 2 " Trade/College" 3 " University+"
label values partner_education partnereduc_labels
tab EDUC_PARTNER partner_education, m

tabulate partner_education dumYES_IPV, chi2

*Status partner*

codebook STATUS_PARTNER
gen partner_citizenship = .
replace partner_citizenship = 1 if inlist(STATUS_PARTNER, 1)  // Canadian
replace partner_citizenship = 2 if inlist(STATUS_PARTNER, 2, 3, 4, 5, 6)  // Immigrant/Refugee/PR
replace partner_citizenship = 3 if inlist(STATUS_PARTNER, 7)  // Unknown
label define statuspartner_labels 1 "Canadian" 2 "Immigrant/Refugee/PR" 3 "Unknown"
label values partner_citizenship statuspartner_labels
tab STATUS_PARTNER partner_citizenship, m

tabulate partner_citizenship dumYES_IPV, exact

codebook STATUS_PARTNER
gen partner_citizenship2 = .
replace partner_citizenship2 = 1 if inlist(STATUS_PARTNER, 1)  // Canadian
replace partner_citizenship2 = 2 if inlist(STATUS_PARTNER, 2, 3, 4, 5, 6)  // Immigrant/Refugee/PR
label define statuspartner_labels2 1 "Canadian" 2 "Immigrant/Refugee/PR"
label values partner_citizenship2 statuspartner_labels2
tab STATUS_PARTNER partner_citizenship2, m

tabulate partner_citizenship2 dumYES_IPV, exact

*Partner employment

codebook EMP_PARTNER_DURING
gen partner_employment = .
replace partner_employment = 1 if inlist(EMP_PARTNER_DURING, 1)  // Employed
replace partner_employment = 2 if inlist(EMP_PARTNER_DURING, 2,3)  //
```

```
replace partner_employment = 2 if inlist(EMP_PARTNER_DURING, 2,3)  // Unemployed
label define partneremployment_labels 1 " Employed" 2 " Unemployed"
label values partner_employment partneremployment_labels
tab EMP_PARTNER_DURING partner_employment, m

tabulate partner_employment dumYES_IPV, chi2

*householde income
codebook HH_INCOME
gen hhincome = .
replace hhincome = 1 if inlist(HH_INCOME, 1, 2)  // <40,000
replace hhincome = 2 if inlist(HH_INCOME, 3, 4)  // 40-69,999
replace hhincome = 3 if inlist(HH_INCOME, 5)  // 70,99,999
replace hhincome = 4 if inlist(HH_INCOME, 6)  // >100,000
label define income_labels 1 "<40,000" 2 "40-69,999" 3 "70,99,999" 4 ">100,000"
label values hhincome income_labels
tab HH_INCOME hhincome, m

tabulate hhincome dumYES_IPV, chi2

*Geography
codebook GEOGRAPHY
gen geography = .
replace geography = 1 if inlist(GEOGRAPHY, 1)  // Eastern
replace geography = 2 if inlist(GEOGRAPHY, 2)  // Central
replace geography = 3 if inlist(GEOGRAPHY, 3, 4)  // Toronto & GTA
replace geography = 4 if inlist(GEOGRAPHY, 5)  // Western
replace geography = 5 if inlist(GEOGRAPHY, 6)  // Northern
label define geography_labels 1 "Eastern" 2 "Central" 3 "Toronto/GTA" 4 "Wester" 5 "Northern"
label values geography geography_labels
tab GEOGRAPHY geography, m

tabulate geography dumYES_IPV, chi2

*Rural/Urban
codebook RURALURBAN
gen ruralurban = .
replace ruralurban = 1 if inlist(RURALURBAN, 1)  // Rural
replace ruralurban = 2 if inlist(RURALURBAN, 2)  // Urban
label define ruralurban_labels 1 "Rural" 2 "Urban"
label values ruralurban ruralurban_labels
tab RURALURBAN ruralurban, m
tabulate RURALURBAN dumYES_IPV, chi2

*Community violence
```

```
gen communityviolence = .
replace communityviolence = 1 if inlist(COMM_VIOL_DURING, 1,3)  // No
replace communityviolence = 2 if inlist(COMM_VIOL_DURING, 2)  // Yes
label define communityviolence_labels 1 "No" 2 "Yes"
label values communityviolence communityviolence_labels
tab COMM_VIOL_DURING communityviolence, m
tabulate communityviolence dumYES_IPV, chi2

**Infomration during Covid
codebook INFORMATION_DURING
gen information = .
replace information = 1 if inlist(INFORMATION_DURING, 1)  // yes
replace information = 2 if inlist(INFORMATION_DURING, 2, 3)  // no/not sure
label define information_labels 1 "Yes" 2 "No"
label values information information_labels
tab INFORMATION_DURING information, m
tabulate information dumYES_IPV, chi2

**Impact of substance use
codebook substanceImpactLevel
* Generate a numeric variable
gen substanceImpactNum = .
replace substanceImpactNum = 1 if substanceImpactLevel == "High Impact"
replace substanceImpactNum = 3 if substanceImpactLevel == "Moderate Impact"
replace substanceImpactNum = 2 if substanceImpactLevel == "Low Impact"

gen substanceImpactBinary = .
replace substanceImpactBinary = 1 if inlist(substanceImpactLevel, "High Impact", "Moderate Impact")
replace substanceImpactBinary = 2 if substanceImpactLevel == "Low Impact"

tabulate substanceImpactLevel substanceImpactNum, missing
tabulate substanceImpactLevel substanceImpactBinary, missing

tabulate substanceImpactBinary dumYES_IPV, chi2

*Number of Children
gen has_children = .
replace has_children = 0 if NUM_CHILD == 1  // None (No Children)
replace has_children = 1 if inlist(NUM_CHILD, 2, 3, 4, 5, 6)  // Has at least 1 child

label variable has_children "Has Children (0 = No, 1 = Yes)"
```

```
label variable has_children "Has Children (0 = No, 1 = Yes)"
label define hasChildrenLabel 0 "No Children" 1 "Has Children"
label values has_children hasChildrenLabel
tabulate NUM_CHILD has_children, missing
tabulate has_children dumYES_IPV, chi2

tabulate NUM_CHILD dumYES_IPV, chi2

gen has_children3 = .
replace has_children3 = 0 if NUM_CHILD == 1  // None (No Children)
replace has_children3 = 1 if inlist(NUM_CHILD, 2, 3, 4, 5, 6)  // Has
at least 1 and 2 children
replace has_children3 = 2 if inlist(NUM_CHILD, 4, 5, 6)  // Has at
least 3+ children
label define has_children3label 0 "No" 1 "1-2children" 2 "3+ children"
label values has_children3 has_children3label
tab NUM_CHILD has_children3, m
tabulate has_children3 dumYES_IPV, chi2

tabulate NUM_CHILD dumYES_IPV, chi2


*Informal Caregiver*

tabulate dumCaregiver dumYES_IPV, chi2

* Health

tabulate PHYSICAL_HEALTH_OUTCOME dumYES_IPV, chi2

tabulate MENTAL_HEALTH_OUTCOME dumYES_IPV, chi2

***Accessing services***

tabulate BARR_HEALTHCR_DURING dumYES_IPV, chi2
prtest dumBarrHEALTHCARE, by(dumYES_IPV)
prtest dumNO_BarrHEALTHCARE, by(dumYES_IPV)
prtest dumNO_TRY_HEALTHCARE, by(dumYES_IPV)

prtest dumTried_HEALTHCARE, by(dumYES_IPV)

tabulate BARR_TRANS_DURING dumYES_IPV, chi2
prtest dumBarrTRANSPORT, by(dumYES_IPV)
prtest dumNO_BarrTRANSPORT, by(dumYES_IPV)
prtest dumNO_TRY_TRANSPORT, by(dumYES_IPV)

tabulate dumTried_TRANSPORT dumYES_IPV, chi2
prtest dumTried_TRANSPORT, by(dumYES_IPV)
```

```
prtest dumTried_TRANSPORT, by(dumYES_IPV)

tabulate BARR_MENT_HC_DURING dumYES_IPV, chi2
prtest dumBarrMENTALH, by(dumYES_IPV)
prtest dumNO_BarrMENTALH, by(dumYES_IPV)
prtest dumNO_TRY_MENTALH, by(dumYES_IPV)

tabulate dumTried_MENTALH dumYES_IPV, chi2
prtest dumTried_MENTALH, by(dumYES_IPV)

tabulate BARR_COUNS_DURING dumYES_IPV, chi2
prtest dumBarrCOUNSELING, by(dumYES_IPV)
prtest dumNO_BarrCOUNSELING, by(dumYES_IPV)
prtest dumNO_TRY_COUNSELING, by(dumYES_IPV)

tabulate dumTried_COUNSELING dumYES_IPV, chi2
prtest dumTried_COUNSELING, by(dumYES_IPV)

tabulate BARR_COMMBASE_DURING dumYES_IPV, chi2
prtest dumBarrCOMMBASW, by(dumYES_IPV)
prtest dumNO_BarrCOMMBASW, by(dumYES_IPV)
prtest dumNO_TRY_COMMBASW, by(dumYES_IPV)

tabulate dumTried_COMMBASW dumYES_IPV, chi2
prtest dumTried_COMMBASW, by(dumYES_IPV)

tabulate BARR_HOUSING_DURING dumYES_IPV, chi2
prtest dumBarrHOUSING, by(dumYES_IPV)
prtest dumNO_BarrHOUSING, by(dumYES_IPV)
prtest dumNO_TRY_HOUSING, by(dumYES_IPV)

tabulate dumTried_HOUSING dumYES_IPV, chi2
prtest dumTried_HOUSING, by(dumYES_IPV)

tabulate BARR_CRISISLINE_DURING dumYES_IPV, chi2
prtest dumBarrCRISISLINE, by(dumYES_IPV)
prtest dumNO_BarrCRISISLINE, by(dumYES_IPV)
prtest dumNO_TRY_CRISISLINE, by(dumYES_IPV)

tabulate dumTried_CRISISLINE dumYES_IPV, chi2
prtest dumTried_CRISISLINE, by(dumYES_IPV)

tabulate BARR_LEGAL_DURING dumYES_IPV, chi2
prtest dumBarrTRANSPORT, by(dumYES_IPV)
prtest dumNO_BarrTRANSPORT, by(dumYES_IPV)
prtest dumNO_TRY_TRANSPORT, by(dumYES_IPV)

tabulate dumTried_LEGAL dumYES_IPV, chi2
```

```
prtest dumTried_LEGAL, by(dumYES_IPV)

tabulate BARR_SETT_DURING dumYES_IPV, chi2
prtest dumBarrLEGAL, by(dumYES_IPV)
prtest dumNO_BarrLEGAL, by(dumYES_IPV)
prtest dumNO_TRY_LEGAL, by(dumYES_IPV)

tabulate dumTried_SETTLEMENT dumYES_IPV, chi2
prtest dumTried_SETTLEMENT, by(dumYES_IPV)

tabulate BARR_ADDICT_DURING dumYES_IPV, chi2
prtest dumBarrADDICT, by(dumYES_IPV)
prtest dumNO_BarrADDICT, by(dumYES_IPV)
prtest dumNO_TRY_ADDICT, by(dumYES_IPV)

tabulate dumTried_ADDICT dumYES_IPV, chi2
prtest dumTried_ADDICT, by(dumYES_IPV)

tabulate BARR_EMER_SHELT_DURING dumYES_IPV, chi2
prtest dumBarrEMERSHELTER, by(dumYES_IPV)
prtest dumNO_BarrEMERSHELTER, by(dumYES_IPV)
prtest dumNO_TRY_EMERSHELTER, by(dumYES_IPV)

tabulate dumTried_EMERSHELTER dumYES_IPV, chi2
prtest dumTried_EMERSHELTER, by(dumYES_IPV)

tabulate BARR_CHILDPROT_DURING dumYES_IPV, chi2
prtest dumBarrCHILDPROTECT, by(dumYES_IPV)
prtest dumNO_BarrCHILDPROTECT, by(dumYES_IPV)
prtest dumNO_TRY_CHILDPROTECT, by(dumYES_IPV)

tabulate dumTried_CHILDPROTECT dumYES_IPV, chi2
prtest dumTried_CHILDPROTECT, by(dumYES_IPV)


tabulate COMM_FAM_DURING dumYES_IPV, chi2
prtest CommFAMILYSIncreased, by(dumYES_IPV)
prtest CommFAMILYdecreased, by(dumYES_IPV)
prtest NoChangeFAMILYCommFRNDS, by(dumYES_IPV)

tabulate COMM_FRND_DURING dumYES_IPV, chi2
prtest CommFRNDSIncreased, by(dumYES_IPV)
prtest CommFRNDSdecreased, by(dumYES_IPV)
prtest NoChangeCommFRNDS, by(dumYES_IPV)

tabulate DecreasedCommunication dumYES_IPV, chi2
prtest dumDecreasedComm2, by(dumYES_IPV)
prtest dumNOTDecreasedComm2, by(dumYES_IPV)
```

```

**

**Diff between tried not tried by IPV non IPV_EXP

codebook dumTried_HEALTHCARE
tabulate dumYES_IPV dumTried_HEALTHCARE, chi2
prtest dumTried_HEALTHCARE, by(dumYES_IPV)

*********************
*LOGISTIC REGRESSION*
*********************

**Research Questions****


*RQ#1 *What is the assocation between experience of IPV and facing
barriers to formal support services during COVID-19 lockdowns?

*Check for multicollinearity*

reg dumYES_IPV dumWoman dumGenders dumLGBTQ2 dumAgePARTIC_18_34
dumAgePARTIC_35_54 dumRacializedPARTIC2 dumIMMIG_PR_REF_PARTIC3
dumTrade_DiplomaEd_PARTIC2 dumUniversityEd_PARTIC2
dumUNEMPLOYEDPARTIC2 dumWomanPartner dumGendersPartner
dumAgePARTNER_18_34 dumAgePARTNER_35_54 dumRacializedPARTNER2
dumIMMIG_PR_REF_PARTNER3 dumTrade_DiplomaEd_PARTNER3
dumUniversityEd_PARTNER3 dumUNEMPLOYEDPARTNER2 dumHHINCOMEUNDER40
dumHHINCOMEUNDER40_69 dumHHINCOMEUNDER70_99 dumEASTONT2 dumCENTRALONT2
 dumWESTONT2 dumNORTHONT2 dumYES_COMMVIOLENCE2 dumNOINFORMATION2
dumSubstanceModHighIMPACT2 dumYes_Children dumCaregiverYES
BadPhysHealth BadMentHealth dumDecreasedComm2
vif

logistic dumBarriersAccess75 dumYES_IPV dumWoman dumGenders dumLGBTQ2
dumAgePARTIC_18_34 dumAgePARTIC_35_54 dumRacializedPARTIC2
dumIMMIG_PR_REF_PARTIC3 dumTrade_DiplomaEd_PARTIC2
dumUniversityEd_PARTIC2 dumUNEMPLOYEDPARTIC2 dumWomanPartner
dumGendersPartner dumAgePARTNER_18_34 dumAgePARTNER_35_54
dumRacializedPARTNER2 dumIMMIG_PR_REF_PARTNER3
dumTrade_DiplomaEd_PARTNER3 dumUniversityEd_PARTNER3
dumUNEMPLOYEDPARTNER2 dumHHINCOMEUNDER40 dumHHINCOMEUNDER40_69
dumHHINCOMEUNDER70_99 dumEASTONT2 dumCENTRALONT2 dumWESTONT2
dumNORTHONT2 dumYES_COMMVIOLENCE2 dumNOINFORMATION2
dumSubstanceModHighIMPACT2 dumYes_Children dumCaregiverYES
BadPhysHealth BadMentHealth dumDecreasedComm2

outreg2 using FormalBarriersFeb25.xls,  alpha (0.001, 0.01, 0.05)
```

```
symbol(***, **, *) append sideway stats(coef se ci pval) eform


*RQ#2 *What is the assocation between experience of IPV and reduced informal supports (i.e., reduced communication with family and friends) during COVID-19 lockdowns?

*Check for multicollinearity*

reg dumYES_IPV dumWoman dumGenders dumLGBTQ2 dumAgePARTIC_18_34 dumAgePARTIC_35_54 dumRacializedPARTIC2 dumIMMIG_PR_REF_PARTIC3 dumTrade_DiplomaEd_PARTIC2 dumUniversityEd_PARTIC2 dumUNEMPLOYEDPARTIC2 dumWomanPartner dumGendersPartner dumAgePARTNER_18_34 dumAgePARTNER_35_54 dumRacializedPARTNER2 dumIMMIG_PR_REF_PARTNER3 dumTrade_DiplomaEd_PARTNER3 dumUniversityEd_PARTNER3 dumUNEMPLOYEDPARTNER2 dumHHINCOMEUNDER40 dumHHINCOMEUNDER40_69 dumHHINCOMEUNDER70_99 dumEASTONT2 dumCENTRALONT2  dumWESTONT2 dumNORTHONT2 dumYES_COMMVIOLENCE2 dumNOINFORMATION2 dumSubstanceModHighIMPACT2 dumYes_Children dumCaregiverYES BadPhysHealth BadMentHealth dumBarriersAccess75
vif

logistic dumDecreasedComm2 dumYES_IPV dumWoman dumGenders dumLGBTQ2 dumAgePARTIC_18_34 dumAgePARTIC_35_54 dumRacializedPARTIC2 dumIMMIG_PR_REF_PARTIC3 dumTrade_DiplomaEd_PARTIC2 dumUniversityEd_PARTIC2 dumUNEMPLOYEDPARTIC2 dumWomanPartner dumGendersPartner dumAgePARTNER_18_34 dumAgePARTNER_35_54 dumRacializedPARTNER2 dumIMMIG_PR_REF_PARTNER3 dumTrade_DiplomaEd_PARTNER3 dumUniversityEd_PARTNER3 dumUNEMPLOYEDPARTNER2 dumHHINCOMEUNDER40 dumHHINCOMEUNDER40_69 dumHHINCOMEUNDER70_99 dumEASTONT2 dumCENTRALONT2 dumWESTONT2 dumNORTHONT2 dumYES_COMMVIOLENCE2 dumNOINFORMATION2 dumSubstanceModHighIMPACT2 dumYes_Children dumCaregiverYES BadPhysHealth BadMentHealth dumBarriersAccess75

outreg2 using FormalBarriersFeb25.xls,  alpha (0.001, 0.01, 0.05) symbol(***, **, *) append sideway stats(coef se ci pval) eform

**********************************************
*SENSITIVITY ANALYSIS** LOOKING AT MEDIATION*
**********************************************

**Research Questions****

*STEP 3*Direct Effect (IPV and Mental Health Predicting Accessing Support)

*RQ#1 *What is the assocation between experience of IPV and facing
```

```
barriers to formal support services during COVID-19 lockdowns?

*Check for multicollinearity*

reg dumYES_IPV dumWoman dumGenders dumLGBTQ2 dumAgePARTIC_18_34
dumAgePARTIC_35_54 dumRacializedPARTIC2 dumIMMIG_PR_REF_PARTIC3
dumTrade_DiplomaEd_PARTIC2 dumUniversityEd_PARTIC2
dumUNEMPLOYEDPARTIC2 dumWomanPartner dumGendersPartner
dumAgePARTNER_18_34 dumAgePARTNER_35_54 dumRacializedPARTNER2
dumIMMIG_PR_REF_PARTNER3 dumTrade_DiplomaEd_PARTNER3
dumUniversityEd_PARTNER3 dumUNEMPLOYEDPARTNER2 dumHHINCOMEUNDER40
dumHHINCOMEUNDER40_69 dumHHINCOMEUNDER70_99 dumEASTONT2 dumCENTRALONT2
 dumWESTONT2 dumNORTHONT2 dumYES_COMMVIOLENCE2 dumNOINFORMATION2
dumSubstanceModHighIMPACT2 dumYes_Children dumCaregiverYES
BadPhysHealth BadMentHealth dumDecreasedComm2
vif

logistic dumBarriersAccess75 dumYES_IPV BadMentHealth dumWoman
dumGenders dumLGBTQ2 dumAgePARTIC_18_34 dumAgePARTIC_35_54
dumRacializedPARTIC2 dumIMMIG_PR_REF_PARTIC3
dumTrade_DiplomaEd_PARTIC2 dumUniversityEd_PARTIC2
dumUNEMPLOYEDPARTIC2 dumWomanPartner dumGendersPartner
dumAgePARTNER_18_34 dumAgePARTNER_35_54 dumRacializedPARTNER2
dumIMMIG_PR_REF_PARTNER3 dumTrade_DiplomaEd_PARTNER3
dumUniversityEd_PARTNER3 dumUNEMPLOYEDPARTNER2 dumHHINCOMEUNDER40
dumHHINCOMEUNDER40_69 dumHHINCOMEUNDER70_99 dumEASTONT2 dumCENTRALONT2
 dumWESTONT2 dumNORTHONT2 dumYES_COMMVIOLENCE2 dumNOINFORMATION2
dumSubstanceModHighIMPACT2 dumYes_Children dumCaregiverYES
BadPhysHealth dumDecreasedComm2

outreg2 using FormalBarriersFeb25.xls,  alpha (0.001, 0.01, 0.05)
symbol(***, **, *) append sideway stats(coef se ci pval) eform


*RQ#2 *What is the assocation between experience of IPV and reduced
informal supports (i.e., reduced communication with family and
friends) during COVID-19 lockdowns?

*Check for multicollinearity*

reg dumYES_IPV dumWoman dumGenders dumLGBTQ2 dumAgePARTIC_18_34
dumAgePARTIC_35_54 dumRacializedPARTIC2 dumIMMIG_PR_REF_PARTIC3
dumTrade_DiplomaEd_PARTIC2 dumUniversityEd_PARTIC2
dumUNEMPLOYEDPARTIC2 dumWomanPartner dumGendersPartner
dumAgePARTNER_18_34 dumAgePARTNER_35_54 dumRacializedPARTNER2
dumIMMIG_PR_REF_PARTNER3 dumTrade_DiplomaEd_PARTNER3
dumUniversityEd_PARTNER3 dumUNEMPLOYEDPARTNER2 dumHHINCOMEUNDER40
dumHHINCOMEUNDER40_69 dumHHINCOMEUNDER70_99 dumEASTONT2 dumCENTRALONT2
```

```
dumHHINCOMEUNDER40_69 dumHHINCOMEUNDER70_99 dumEASTONT2 dumCENTRALONT2
 dumWESTONT2 dumNORTHONT2 dumYES_COMMVIOLENCE2 dumNOINFORMATION2
dumSubstanceModHighIMPACT2 dumYes_Children dumCaregiverYES
BadPhysHealth BadMentHealth dumBarriersAccess75
vif

logistic dumDecreasedComm2 dumYES_IPV dumWoman dumGenders dumLGBTQ2
dumAgePARTIC_18_34 dumAgePARTIC_35_54 dumRacializedPARTIC2
dumIMMIG_PR_REF_PARTIC3 dumTrade_DiplomaEd_PARTIC2
dumUniversityEd_PARTIC2 dumUNEMPLOYEDPARTIC2 dumWomanPartner
dumGendersPartner dumAgePARTNER_18_34 dumAgePARTNER_35_54
dumRacializedPARTNER2 dumIMMIG_PR_REF_PARTNER3
dumTrade_DiplomaEd_PARTNER3 dumUniversityEd_PARTNER3
dumUNEMPLOYEDPARTNER2 dumHHINCOMEUNDER40 dumHHINCOMEUNDER40_69
dumHHINCOMEUNDER70_99 dumEASTONT2 dumCENTRALONT2 dumWESTONT2
dumNORTHONT2 dumYES_COMMVIOLENCE2 dumNOINFORMATION2
dumSubstanceModHighIMPACT2 dumYes_Children dumCaregiverYES
BadPhysHealth BadMentHealth dumBarriersAccess75

outreg2 using FormalBarriersFeb25.xls,  alpha (0.001, 0.01, 0.05)
symbol(***, **, *) append sideway stats(coef se ci pval) eform


*Checking to see if health is a mediator for FORMAL*

*STEP 1* : Total Effect of IPV on Accessing Support (Without Mediator)

*RQ#1 *What is the assocation between experience of IPV and facing
barriers to formal support services during COVID-19 lockdowns?

*Check for multicollinearity*

reg dumYES_IPV dumWoman dumGenders dumLGBTQ2 dumAgePARTIC_18_34
dumAgePARTIC_35_54 dumRacializedPARTIC2 dumIMMIG_PR_REF_PARTIC3
dumTrade_DiplomaEd_PARTIC2 dumUniversityEd_PARTIC2
dumUNEMPLOYEDPARTIC2 dumWomanPartner dumGendersPartner
dumAgePARTNER_18_34 dumAgePARTNER_35_54 dumRacializedPARTNER2
dumIMMIG_PR_REF_PARTNER3 dumTrade_DiplomaEd_PARTNER3
dumUniversityEd_PARTNER3 dumUNEMPLOYEDPARTNER2 dumHHINCOMEUNDER40
dumHHINCOMEUNDER40_69 dumHHINCOMEUNDER70_99 dumEASTONT2 dumCENTRALONT2
 dumWESTONT2 dumNORTHONT2 dumYES_COMMVIOLENCE2 dumNOINFORMATION2
dumSubstanceModHighIMPACT2 dumYes_Children dumCaregiverYES
dumDecreasedComm2
vif

logistic dumBarriersAccess75 dumYES_IPV dumWoman dumGenders dumLGBTQ2
dumAgePARTIC_18_34 dumAgePARTIC_35_54 dumRacializedPARTIC2
dumIMMIG_PR_REF_PARTIC3 dumTrade_DiplomaEd_PARTIC2
```

```
dumUniversityEd_PARTIC2 dumUNEMPLOYEDPARTIC2 dumWomanPartner dumGendersPartner dumAgePARTNER_18_34 dumAgePARTNER_35_54 dumRacializedPARTNER2 dumIMMIG_PR_REF_PARTNER3 dumTrade_DiplomaEd_PARTNER3 dumUniversityEd_PARTNER3 dumUNEMPLOYEDPARTNER2 dumHHINCOMEUNDER40 dumHHINCOMEUNDER40_69 dumHHINCOMEUNDER70_99 dumEASTONT2 dumCENTRALONT2 dumWESTONT2 dumNORTHONT2 dumYES_COMMVIOLENCE2 dumNOINFORMATION2 dumSubstanceModHighIMPACT2 dumYes_Children dumCaregiverYES dumDecreasedComm2

*Step 2: IPV Predicting Mental Health (Mediator Path)

*Check for multicollinearity*
*Mental Health*
reg dumYES_IPV dumWoman dumGenders dumLGBTQ2 dumAgePARTIC_18_34 dumAgePARTIC_35_54 dumRacializedPARTIC2 dumIMMIG_PR_REF_PARTIC3 dumTrade_DiplomaEd_PARTIC2 dumUniversityEd_PARTIC2 dumUNEMPLOYEDPARTIC2 dumWomanPartner dumGendersPartner dumAgePARTNER_18_34 dumAgePARTNER_35_54 dumRacializedPARTNER2 dumIMMIG_PR_REF_PARTNER3 dumTrade_DiplomaEd_PARTNER3 dumUniversityEd_PARTNER3 dumUNEMPLOYEDPARTNER2 dumHHINCOMEUNDER40 dumHHINCOMEUNDER40_69 dumHHINCOMEUNDER70_99 dumEASTONT2 dumCENTRALONT2  dumWESTONT2 dumNORTHONT2 dumYES_COMMVIOLENCE2 dumNOINFORMATION2 dumSubstanceModHighIMPACT2 dumYes_Children dumCaregiverYES dumDecreasedComm2 BadPhysHealth
vif

logistic BadMentHealth dumYES_IPV dumWoman dumGenders dumLGBTQ2 dumAgePARTIC_18_34 dumAgePARTIC_35_54 dumRacializedPARTIC2 dumIMMIG_PR_REF_PARTIC3 dumTrade_DiplomaEd_PARTIC2 dumUniversityEd_PARTIC2 dumUNEMPLOYEDPARTIC2 dumWomanPartner dumGendersPartner dumAgePARTNER_18_34 dumAgePARTNER_35_54 dumRacializedPARTNER2 dumIMMIG_PR_REF_PARTNER3 dumTrade_DiplomaEd_PARTNER3 dumUniversityEd_PARTNER3 dumUNEMPLOYEDPARTNER2 dumHHINCOMEUNDER40 dumHHINCOMEUNDER40_69 dumHHINCOMEUNDER70_99 dumEASTONT2 dumCENTRALONT2 dumWESTONT2 dumNORTHONT2 dumYES_COMMVIOLENCE2 dumNOINFORMATION2 dumSubstanceModHighIMPACT2 dumYes_Children dumCaregiverYES dumDecreasedComm2 BadPhysHealth

*Physical Health*

reg dumYES_IPV dumWoman dumGenders dumLGBTQ2 dumAgePARTIC_18_34 dumAgePARTIC_35_54 dumRacializedPARTIC2 dumIMMIG_PR_REF_PARTIC3 dumTrade_DiplomaEd_PARTIC2 dumUniversityEd_PARTIC2 dumUNEMPLOYEDPARTIC2 dumWomanPartner dumGendersPartner dumAgePARTNER_18_34 dumAgePARTNER_35_54 dumRacializedPARTNER2 dumIMMIG_PR_REF_PARTNER3 dumTrade_DiplomaEd_PARTNER3
```

```
dumUniversityEd_PARTNER3 dumUNEMPLOYEDPARTNER2 dumHHINCOMEUNDER40
dumHHINCOMEUNDER40_69 dumHHINCOMEUNDER70_99 dumEASTONT2 dumCENTRALONT2
 dumWESTONT2 dumNORTHONT2 dumYES_COMMVIOLENCE2 dumNOINFORMATION2
dumSubstanceModHighIMPACT2 dumYes_Children dumCaregiverYES
dumDecreasedComm2 BadMentHealth
vif

logistic BadPhysHealth dumYES_IPV dumWoman dumGenders dumLGBTQ2
dumAgePARTIC_18_34 dumAgePARTIC_35_54 dumRacializedPARTIC2
dumIMMIG_PR_REF_PARTIC3 dumTrade_DiplomaEd_PARTIC2
dumUniversityEd_PARTIC2 dumUNEMPLOYEDPARTIC2 dumWomanPartner
dumGendersPartner dumAgePARTNER_18_34 dumAgePARTNER_35_54
dumRacializedPARTNER2 dumIMMIG_PR_REF_PARTNER3
dumTrade_DiplomaEd_PARTNER3 dumUniversityEd_PARTNER3
dumUNEMPLOYEDPARTNER2 dumHHINCOMEUNDER40 dumHHINCOMEUNDER40_69
dumHHINCOMEUNDER70_99 dumEASTONT2 dumCENTRALONT2 dumWESTONT2
dumNORTHONT2 dumYES_COMMVIOLENCE2 dumNOINFORMATION2
dumSubstanceModHighIMPACT2 dumYes_Children dumCaregiverYES
dumDecreasedComm2 BadMentHealth


*FOR INFORMAL*

*STEP 1
*RQ#2 *What is the assocation between experience of IPV and reduced
informal supports (i.e., reduced communication with family and
friends) during COVID-19 lockdowns?

*Check for multicollinearity*

reg dumYES_IPV dumWoman dumGenders dumLGBTQ2 dumAgePARTIC_18_34
dumAgePARTIC_35_54 dumRacializedPARTIC2 dumIMMIG_PR_REF_PARTIC3
dumTrade_DiplomaEd_PARTIC2 dumUniversityEd_PARTIC2
dumUNEMPLOYEDPARTIC2 dumWomanPartner dumGendersPartner
dumAgePARTNER_18_34 dumAgePARTNER_35_54 dumRacializedPARTNER2
dumIMMIG_PR_REF_PARTNER3 dumTrade_DiplomaEd_PARTNER3
dumUniversityEd_PARTNER3 dumUNEMPLOYEDPARTNER2 dumHHINCOMEUNDER40
dumHHINCOMEUNDER40_69 dumHHINCOMEUNDER70_99 dumEASTONT2 dumCENTRALONT2
 dumWESTONT2 dumNORTHONT2 dumYES_COMMVIOLENCE2 dumNOINFORMATION2
dumSubstanceModHighIMPACT2 dumYes_Children dumCaregiverYES
BadPhysHealth BadMentHealth dumBarriersAccess75
vif

logistic dumDecreasedComm2 dumYES_IPV dumWoman dumGenders dumLGBTQ2
dumAgePARTIC_18_34 dumAgePARTIC_35_54 dumRacializedPARTIC2
dumIMMIG_PR_REF_PARTIC3 dumTrade_DiplomaEd_PARTIC2
dumUniversityEd_PARTIC2 dumUNEMPLOYEDPARTIC2 dumWomanPartner
dumGendersPartner dumAgePARTNER_18_34 dumAgePARTNER_35_54
```

```
dumRacializedPARTNER2 dumIMMIG_PR_REF_PARTNER3
dumTrade_DiplomaEd_PARTNER3 dumUniversityEd_PARTNER3
dumUNEMPLOYEDPARTNER2 dumHHINCOMEUNDER40 dumHHINCOMEUNDER40_69
dumHHINCOMEUNDER70_99 dumEASTONT2 dumCENTRALONT2 dumWESTONT2
dumNORTHONT2 dumYES_COMMVIOLENCE2 dumNOINFORMATION2
dumSubstanceModHighIMPACT2 dumYes_Children dumCaregiverYES
BadPhysHealth BadMentHealth dumBarriersAccess75

*STEP 2

*RQ#2 *What is the assocation between experience of IPV and reduced
informal supports (i.e., reduced communication with family and
friends) during COVID-19 lockdowns?

*Check for multicollinearity*

reg dumYES_IPV dumWoman dumGenders dumLGBTQ2 dumAgePARTIC_18_34
dumAgePARTIC_35_54 dumRacializedPARTIC2 dumIMMIG_PR_REF_PARTIC3
dumTrade_DiplomaEd_PARTIC2 dumUniversityEd_PARTIC2
dumUNEMPLOYEDPARTIC2 dumWomanPartner dumGendersPartner
dumAgePARTNER_18_34 dumAgePARTNER_35_54 dumRacializedPARTNER2
dumIMMIG_PR_REF_PARTNER3 dumTrade_DiplomaEd_PARTNER3
dumUniversityEd_PARTNER3 dumUNEMPLOYEDPARTNER2 dumHHINCOMEUNDER40
dumHHINCOMEUNDER40_69 dumHHINCOMEUNDER70_99 dumEASTONT2 dumCENTRALONT2
 dumWESTONT2 dumNORTHONT2 dumYES_COMMVIOLENCE2 dumNOINFORMATION2
dumSubstanceModHighIMPACT2 dumYes_Children dumCaregiverYES
dumBarriersAccess75
vif

logistic dumDecreasedComm2 dumYES_IPV dumWoman dumGenders dumLGBTQ2
dumAgePARTIC_18_34 dumAgePARTIC_35_54 dumRacializedPARTIC2
dumIMMIG_PR_REF_PARTIC3 dumTrade_DiplomaEd_PARTIC2
dumUniversityEd_PARTIC2 dumUNEMPLOYEDPARTIC2 dumWomanPartner
dumGendersPartner dumAgePARTNER_18_34 dumAgePARTNER_35_54
dumRacializedPARTNER2 dumIMMIG_PR_REF_PARTNER3
dumTrade_DiplomaEd_PARTNER3 dumUniversityEd_PARTNER3
dumUNEMPLOYEDPARTNER2 dumHHINCOMEUNDER40 dumHHINCOMEUNDER40_69
dumHHINCOMEUNDER70_99 dumEASTONT2 dumCENTRALONT2 dumWESTONT2
dumNORTHONT2 dumYES_COMMVIOLENCE2 dumNOINFORMATION2
dumSubstanceModHighIMPACT2 dumYes_Children dumCaregiverYES
dumBarriersAccess75

*STEP 3

*RQ#2 *What is the assocation between experience of IPV and reduced
informal supports (i.e., reduced communication with family and
friends) during COVID-19 lockdowns?
```

```
*Check for multicollinearity*

reg dumYES_IPV dumWoman dumGenders dumLGBTQ2 dumAgePARTIC_18_34 dumAgePARTIC_35_54 dumRacializedPARTIC2 dumIMMIG_PR_REF_PARTIC3 dumTrade_DiplomaEd_PARTIC2 dumUniversityEd_PARTIC2 dumUNEMPLOYEDPARTIC2 dumWomanPartner dumGendersPartner dumAgePARTNER_18_34 dumAgePARTNER_35_54 dumRacializedPARTNER2 dumIMMIG_PR_REF_PARTNER3 dumTrade_DiplomaEd_PARTNER3 dumUniversityEd_PARTNER3 dumUNEMPLOYEDPARTNER2 dumHHINCOMEUNDER40 dumHHINCOMEUNDER40_69 dumHHINCOMEUNDER70_99 dumEASTONT2 dumCENTRALONT2 dumWESTONT2 dumNORTHONT2 dumYES_COMMVIOLENCE2 dumNOINFORMATION2 dumSubstanceModHighIMPACT2 dumYes_Children dumCaregiverYES BadPhysHealth BadMentHealth dumBarriersAccess75
vif

logistic dumDecreasedComm2 dumYES_IPV dumWoman dumGenders dumLGBTQ2 dumAgePARTIC_18_34 dumAgePARTIC_35_54 dumRacializedPARTIC2 dumIMMIG_PR_REF_PARTIC3 dumTrade_DiplomaEd_PARTIC2 dumUniversityEd_PARTIC2 dumUNEMPLOYEDPARTIC2 dumWomanPartner dumGendersPartner dumAgePARTNER_18_34 dumAgePARTNER_35_54 dumRacializedPARTNER2 dumIMMIG_PR_REF_PARTNER3 dumTrade_DiplomaEd_PARTNER3 dumUniversityEd_PARTNER3 dumUNEMPLOYEDPARTNER2 dumHHINCOMEUNDER40 dumHHINCOMEUNDER40_69 dumHHINCOMEUNDER70_99 dumEASTONT2 dumCENTRALONT2 dumWESTONT2 dumNORTHONT2 dumYES_COMMVIOLENCE2 dumNOINFORMATION2 dumSubstanceModHighIMPACT2 dumYes_Children dumCaregiverYES BadPhysHealth BadMentHealth dumBarriersAccess75

***Supplement 2****
***Family separately

logistic CommFAMILYdecreased2 dumYES_IPV dumWoman dumGenders dumLGBTQ2 dumAgePARTIC_18_34 dumAgePARTIC_35_54 dumRacializedPARTIC2 dumIMMIG_PR_REF_PARTIC3 dumTrade_DiplomaEd_PARTIC2 dumUniversityEd_PARTIC2 dumUNEMPLOYEDPARTIC2 dumWomanPartner dumGendersPartner dumAgePARTNER_18_34 dumAgePARTNER_35_54 dumRacializedPARTNER2 dumIMMIG_PR_REF_PARTNER3 dumTrade_DiplomaEd_PARTNER3 dumUniversityEd_PARTNER3 dumUNEMPLOYEDPARTNER2 dumHHINCOMEUNDER40 dumHHINCOMEUNDER40_69 dumHHINCOMEUNDER70_99 dumEASTONT2 dumCENTRALONT2 dumWESTONT2 dumNORTHONT2 dumYES_COMMVIOLENCE2 dumNOINFORMATION2 dumSubstanceModHighIMPACT2 dumYes_Children dumCaregiverYES BadPhysHealth BadMentHealth dumBarriersAccess75

***Friends separately

logistic CommFRNDSdecreased2 dumYES_IPV dumWoman dumGenders dumLGBTQ2 dumAgePARTIC_18_34 dumAgePARTIC_35_54 dumRacializedPARTIC2
```
