# Supplementary material for: Locked down, locked out: a cross-sectional study on experiences of intimate partner violence (IPV) and barriers to formal and informal support during COVID-19 lockdowns in Ontario
Source: BMC Public Health. 2025 Nov 17;25:3962. doi: 10.1186/s12889-025-25124-7 (PMC12621413; doi:10.1186/s12889-025-25124-7)
Supplement: Supplementary file 3 — Supplementary Material 3: Additional file 3: Additional analysis (sub-analysis and exploratory mediation analysis). [file 12889_2025_25124_MOESM3_ESM.docx]

**Additional File 3**

**Sub-analysis: Decreased Communication with Family and Friends**

This supplementary appendix presents a sub-analysis to further investigate decreased communication with informal supports, specifically examining whether the effect varies when analyzing communication with family and friends separately. Given the importance of social networks in providing support for IPV survivors, we explored whether specific factors influenced decreased communication differently depending on the type of informal support.

**Methods** To assess whether predictors of decreased communication differed by type of informal support, we conducted separate logistic regression models for decreased communication with family and decreased communication with friends. We compared these results to the composite measure of decreased communication with informal supports used in the primary analysis.

**Results**

**Step 1: Total Effect (IPV → Decreased Communication with Informal Supports)**

| **Predictor** | **Odds Ratio (OR)** | **Std. Error** | **z-value** | **p-value** | **95% CI** |
| --- | --- | --- | --- | --- | --- |
| IPV | 1.57 | 0.31 | 2.25 | 0.025 | (1.06, 2.31) |

**Step 2: Gender Diverse/Non-Disclosure and Decreased Communication with Informal Supports**

- In the combined model, participants with gender diverse or non-disclosed partners were 5.35 times more likely than those with male partners to report decreased communication with family and friends (OR = 5.35, p = 0.031, [95% CI: 1.17, 24.55)).
- When examining communication with family separately, this effect was even stronger: gender diverse or non-disclosed partners were 7.51 times more likely than those with male partners to report decreased communication (OR = 7.51, p = 0.009 [95% CI: 1.66, 33.89]).
- However, when examining communication with friends separately, the association was no longer statistically significant (OR = 1.82, p = 0.398[ 95% CI: 0.45, 7.26]).

**Interpretation** These findings suggest that the effect of gender diverse or non-disclosed partners on decreased communication is primarily driven by reduced communication with family rather than with friends. This could reflect unique challenges in maintaining family connections for individuals with gender diverse or non-disclosed partners, potentially due to stigma, estrangement, or difficulties in navigating family relationships during IPV experiences. Note the confidence intervals are quite wide possibly due to small sample size in the category however the association remains strong.

**Conclusion** This sub-analysis highlights that the relationship between IPV and decreased communication with informal supports among marginalized groups may vary depending on the type of support network.

**Mediation analysis to see if poor mental/physical health mediate the relationship between IPV and barriers to accessing formal supports**

**Overview of Mediation Analysis**

This supplementary appendix presents a mediation analysis testing whether poor mental/physical health mediates the relationship between IPV (intimate partner violence) and barriers to accessing formal support.

To test for mediation, we conducted three logistic regression models:

1. Total Effect Model: Examining the association between IPV and barriers to accessing formal support.
2. Mediator Model: Examining the association between (i) IPV and poor mental health & (ii) IPV and poor Physical health
3. Direct and Indirect Effects Model: Including both IPV and poor mental/physical health in the model predicting barriers to accessing formal support.

If mediation exists, we expect the effect of IPV on accessing support to decrease significantly after including poor mental/physical health in the model. If the effect remains largely unchanged and perceived mental/physical health does not significantly predict access to support, this suggests no mediation.

**Results**

**Step 1: Total Effect (IPV → Accessing Formal Support)**

| **Predictor** | **Odds Ratio (OR)** | **Std. Error** | **z-value** | **p-value** | **95% CI** |
| --- | --- | --- | --- | --- | --- |
| IPV | **3.67** | 0.85 | 5.62 | <0.001 | (2.33, 5.77) |

Interpretation: IPV survivors had 3.67 times higher odds of experiencing barriers to accessing formal support compared to non-IPV individuals (p < 0.001). This indicates a strong total effect of IPV on access to formal support.

**Step 2: IPV Predicting Poor Mental & Physical Health (IV → Mediator)**

| **Predictor** | **Odds Ratio (OR)** | **Std. Error** | **z-value** | **p-value** | **95% CI** |
| --- | --- | --- | --- | --- | --- |
| IPV on Mental Health | **1.88** | 0.43 | 2.75 | 0.006 | (1.199, 2.959) |
| IPV on Physical Health | 1.42 | 0.32 | 1.57 | 0.117 | (0.586,1.515) |

Interpretation: IPV survivors had 1.88 times higher odds of reporting poor mental health compared to non-IPV individuals (p = 0.016). This suggests that IPV significantly affects poor mental health, supporting its potential role as a mediator. There was no significant effects on poor physical health.

**Step 3: Direct Effect (IPV and Mental Health → Accessing Support)**

| **Predictor** | **Odds Ratio (OR)** | **Std. Error** | **z-value** | **p-value** | **95% CI** |
| --- | --- | --- | --- | --- | --- |
| IPV | **3.41** | 0.79 | 5.27 | <0.001 | (2.16, 5.38) |
| Poor Mental Health | **1.22** | 0.29 | 0.82 | 0.410 | (0.76, 1.95) |
| Poor Physical Health | **1.65** | 0.30 | 02.18 | 0.039 | (1.05,2.60) |

**Interpretation:**

- The effect of IPV on barriers to accessing formal support remains strong (OR = 3.41, p < 0.001), with only a slight reduction from Step 1 (OR = 3.67).
- Poor mental health does not significantly predict barriers to accessing formal support (p = 0.41), indicating no evidence of mediation through mental health.
- Poor physical health significantly predicts barriers to accessing formal support (p = 0.039), suggesting some mediation through worse physical health.

**Conclusion**:

This analysis provides partial evidence of mediation in the relationship between IPV and barriers to accessing formal support.

- Poor mental health does not mediate the relationship (p = 0.41), as it does not significantly predict access barriers.
- Poor physical health partially mediates the relationship (p = 0.039), indicating that IPV survivors who experience worse physical health may face additional challenges in accessing formal support. This makes sense during COVID-19 when everyone faced barriers to accessing formal services.
- However, IPV remains a strong and direct predictor of access barriers.

**Mediation analysis to see if poor mental/physical health mediate the relationship between IPV and decreased communication with informal supports**

**Overview of Mediation Analysis**

This supplementary appendix presents a mediation analysis testing whether poor mental/physical health mediates the relationship between IPV and decreased communication with informal supports.

To test for mediation, we conducted three logistic regression models:

1. Total Effect Model: Examining the association between IPV and decreased communication with informal support (no mental or physical health).
2. Mediator Model: Examining the association between (i) IPV and poor mental health & (ii) IPV and poor physical health
3. Direct and Indirect Effects Model: Including both IPV and worse mental/physical health in the model predicting decreased communication with informal supports.

If mediation exists, we expect the effect of IPV on decreased communication with informal supports to decrease significantly after including poor mental/physical health. If the effect remains largely unchanged and poor mental or physical health do not significantly predict access to support, this suggests no mediation.

**Results**

**Step 1: Total Effect (IPV → Decreased Communication with Informal Support)**

| **Predictor** | **Odds Ratio (OR)** | **Std. Error** | **z-value** | **p-value** | **95% CI** |
| --- | --- | --- | --- | --- | --- |
| IPV | **1.77** | 0.34 | 2.92 | 0.003 | (1.21, 2.59) |

Interpretation: IPV survivors had 1.77 times higher odds of reporting decreased communication with informal support compared to non-IPV individuals (p = 0.003). This indicates a strong total effect of IPV on decreased communication.

**Step 2: IPV Predicting Poor Mental & Physical Health (IV → Mediator)**

| **Predictor** | **Odds Ratio (OR)** | **Std. Error** | **z-value** | **p-value** | **95% CI** |
| --- | --- | --- | --- | --- | --- |
| IPV on Poor Mental Health | **1.88** | 0.43 | 2.75 | 0.006 | (1.199, 2.959) |
| IPV on Poor Physical Health | 1.42 | 0.32 | 1.57 | 0.117 | (0.586,1.515) |

Interpretation: IPV survivors had 1.88 times higher odds of reporting poor mental health compared to non-IPV individuals (p = 0.016). This suggests that IPV significantly affects mental health, supporting its potential role as a mediator. There was no significant effects on physical health.

**Step 3: Direct Effect (IPV, Mental Health, and Physical Health → Decreased Communication)**

| Predictor | Odds Ratio (OR) | Std. Error | z-value | p-value | 95% CI |
| --- | --- | --- | --- | --- | --- |
| IPV | **1.57** | 0.31 | 2.25 | 0.025 | (1.06, 2.31) |
| Poor Mental Health | **1.76** | 0.27 | 3.67 | <0.001 | (1.30, 2.37) |
| Poor Physical Health | **1.96** | 0.32 | 4.17 | <0.001 | (1.43, 2.70) |

**Interpretation:** When poor mental and physical health are added to the model:

- The effect of IPV on decreased communication remains significant (OR = 1.57, p = 0.025), though slightly reduced from Step 1 (OR = 1.77).
- Both poor mental health (p < 0.001) and poor physical health (p < 0.001) significantly predict decreased communication, suggesting partial mediation.

**Conclusion**

The mediation analysis provides strong evidence that poor mental health and poor physical health partially mediate the relationship between IPV and decreased communication with informal support. While IPV significantly increases the likelihood of decreased communication with informal supports, poor mental and physical health further explain this association, though IPV remains a significant predictor.
